# Probing tumor microenvironment in patients with newly diagnosed glioblastoma during chemoradiation and adjuvant temozolomide with functional MRI

**DOI:** 10.1038/s41598-018-34820-x

**Published:** 2018-11-20

**Authors:** K. Ina Ly, Bella Vakulenko-Lagun, Kyrre E. Emblem, Yangming Ou, Xiao Da, Rebecca A. Betensky, Jayashree Kalpathy-Cramer, Dan G. Duda, Rakesh K. Jain, Andrew S. Chi, Scott R. Plotkin, Tracy T. Batchelor, Gregory Sorensen, Bruce R. Rosen, Elizabeth R. Gerstner

**Affiliations:** 1 Stephen E. and Catherine Pappas Center for Neuro-Oncology, Department of Neurology, Massachusetts General Hospital, Boston, MA USA; 2 Department of Biostatistics, Harvard T.H. Chan School of Public Health, Boston, MA USA; 3 Department of Diagnostic Physics, Division of Radiology and Nuclear Medicine, Oslo University Hospital, Oslo, Norway; 4 Department of Pediatrics and Radiology, Boston Children’s Hospital, Boston, MA USA; 5 Functional Neuroimaging Laboratory, Brigham and Women’s Hospital, Boston, MA USA; 6 Athinoula A. Martinos Center for Biomedical Imaging, Department of Radiology, Massachusetts General Hospital, Charlestown, MA USA; 7 Edwin L. Steele Laboratories, Department of Radiation Oncology, Massachusetts General Hospital, Boston, MA USA; 8 Laura and Isaac Perlmutter Cancer Center, New York University Langone Health, New York, NY USA; 9 IMRIS, Deerfield Imaging, Minnetonka, MN USA

**Keywords:** Cancer imaging, Cancer microenvironment, Head and neck cancer

## Abstract

Functional MRI may identify critical windows of opportunity for drug delivery and distinguish between early treatment responders and non-responders. Using diffusion-weighted, dynamic contrast-enhanced, and dynamic susceptibility contrast MRI, as well as pro-angiogenic and pro-inflammatory blood markers, we prospectively studied the physiologic tumor-related changes in fourteen newly diagnosed glioblastoma patients during standard therapy. 153 MRI scans and blood collection were performed before chemoradiation (baseline), weekly during chemoradiation (week 1–6), monthly before each cycle of adjuvant temozolomide (pre-C1-C6), and after cycle 6. The apparent diffusion coefficient, volume transfer coefficient (K^trans^), and relative cerebral blood volume (rCBV) and flow (rCBF) were calculated within the tumor and edema regions and compared to baseline. Cox regression analysis was used to assess the effect of clinical variables, imaging, and blood markers on progression-free (PFS) and overall survival (OS). After controlling for additional covariates, high baseline rCBV and rCBF within the edema region were associated with worse PFS (microvessel rCBF: HR = 7.849, *p* = 0.044; panvessel rCBV: HR = 3.763, *p* = 0.032; panvessel rCBF: HR = 3.984; *p* = 0.049). The same applied to high week 5 and pre-C1 K^trans^ within the tumor region (week 5 K^trans^: HR = 1.038, *p* = 0.003; pre-C1 K^trans^: HR = 1.029, *p* = 0.004). Elevated week 6 VEGF levels were associated with worse OS (HR = 1.034; *p* = 0.004). Our findings suggest a role for rCBV and rCBF at baseline and K^trans^ and VEGF levels during treatment as markers of response. Functional imaging changes can differ substantially between tumor and edema regions, highlighting the variable biologic and vascular state of tumor microenvironment during therapy.

## Introduction

Glioblastoma (GBM) is an aggressive primary tumor of the central nervous system (CNS) that carries a median overall survival (OS) of less than 15 months with standard therapy^1^. Anatomical T1-weighted post-contrast and T2-weighted fluid-attenuated inversion recovery (FLAIR) sequences on magnetic resonance imaging (MRI) form the basis of the Response Assessment in Neuro-Oncology (RANO) criteria^2^ but do not always reflect underlying tumor biology. This is illustrated by the similar radiographic appearance of tumor progression and treatment-related inflammation in some cases (“pseudoprogression”), as well as the decrease of contrast enhancement and FLAIR hyperintensity that occurs in the setting of anti-angiogenic therapy and creates the deceptive impression of improved tumor burden (“pseudoresponse”)^3^. The occurrence of pseudoprogression also precludes the use of T1-weighted post-contrast and FLAIR sequences to assess a patient’s response to therapy early during the course of concurrent radiation and chemotherapy as inflammation peaks during this time and can persist for three months or more after completion of radiation^3^. Thus, other non-invasive biomarkers are needed to distinguish between early responders versus non-responders.

Diffusion- and perfusion-weighted MRI measure water diffusion in tissue compartments and blood volume and blood flow, respectively, and are non-invasive techniques to probe tumor physiology. In addition, dynamic contrast-enhanced (DCE) MRI, a type of perfusion-weighted imaging, has the ability to measure vessel permeability and may represent a potential tool to identify time points when the blood-brain barrier (BBB) is particularly permeable and permissive to drug delivery.

In this prospective single-center study, we studied the functional MRI changes in patients with newly diagnosed GBM treated with six weeks of concurrent fractionated radiation (RT) and daily temozolomide (“chemoradiation” (CRT)) and adjuvant monthly temozolomide (TMZ; collectively referred to as “standard therapy”) at multiple time points. In addition, we evaluated the changes in known pro-angiogenic and pro-inflammatory peripheral blood markers in response to treatment. The goal of this exploratory study was to obtain a detailed understanding of the impact of treatment on tumor physiology and vascular state and identify time points during which treatment could be optimized.

## Materials and Methods

### Study Design

**Figure 1:**
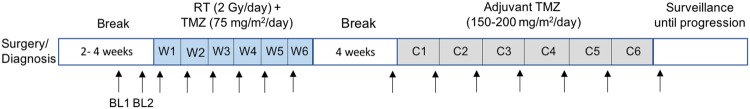
Timeline of clinical study. Arrows indicate time points of MRI acquisition and peripheral blood collection. BL1, BL2 = baseline 1, baseline 2; W1–6 = weeks 1–6 of chemoradiation; C1-C6: cycles 1–6 of adjuvant temozolomide.

Following Institutional Review Board (IRB) approval by the Office for Human Research Studies of the Dana Farber/Harvard Cancer Center, patients with histologically confirmed GBM were prospectively enrolled in this study (NCT00756106). All participants provided informed consent and signed an IRB-approved consent form. All experiments were performed in accordance with the guidelines and regulations of the IRB and were conducted in compliance with the Health Insurance Portability and Accountability Act. Patient inclusion and exclusion criteria are shown in Supplementary Figure 1. Patients underwent MR imaging and collection of peripheral blood at the following time points: 3–4 days before starting CRT (“baseline 1”), 1 day before starting CRT (“baseline 2”), at the beginning of each week of CRT (“week 1–6”), and monthly before each cycle of adjuvant TMZ until disease progression or until completion of six cycles of adjuvant TMZ (whichever one occurred first; “pre-C1-C6”; Fig. 1). The last scan occurred four weeks after completion of adjuvant TMZ (post-C6). Patients had to be on a stable dose of corticosteroids for five days before each MRI. Overall survival (OS) was defined as the time from histologic diagnosis to death or time of last follow-up. Progression-free survival (PFS) was defined as the time from histologic diagnosis to progression, with progression defined based on the Response Assessment in Neuro-Oncology (RANO) criteria for high-grade gliomas^2^.

### MRI Sequence Acquisition

MR data were acquired with a 3.0 T magnetic resonance scanner (TIM Trio, Siemens Healthcare, Erlangen, Germany) using a 32-channel phased-array head coil. MRI sequences included scout, pre- and post-contrast T1-weighted images, T2-weighted FLAIR images, dynamic susceptibility contrast (DSC) imaging, DCE imaging, and diffusion tensor imaging (DTI) as previously described^4^. Scout images were acquired and scan-to-scan reproducibility improved by using the “AutoAlign” method^5,6^. Axial 2D FLAIR images were obtained with a TR = 10,000 ms, TE = 70 ms, 5-mm slice thickness, 1-mm inter slice gap, 0.43-mm in-plane resolution, 23 slices, and a 512 × 512 matrix. Pre-contrast axial, spin-echo T1 images were obtained prior to contrast injection, using TR = 600 ms, TE = 12 ms, 5-mm slice thickness, 1-mm interslice gap, 0.43-mm in-plane resolution, 23 slices, and a 512 × 512 matrix. DCE data were acquired by using a fast-gradient echo sequence with a TR = 6.8 ms, TE = 2.73 ms, 2.11-mm slice thickness, 0-mm interslice gap, 20 slices, 1.8-mm in-plane resolution, 128 × 128 matrix, and FOV 230 × 230 mm^2^ and flip angle 10°. 60 frames with these parameters were acquired over 6 minutes. A fixed tissue T1-value of 1000 ms was used to compute DCE maps. After 52 seconds, a bolus of 0.1 mMol/kg of GD-DTPA (gadopentetic acid) was injected. DSC imaging was then acquired, consisting of a combined gradient-echo and spin-echo EPI sequence, using TR = 1480 ms, TE1/TE2 = 32/93 ms, 5-mm slice thickness, 1.5-mm interslice gap, 12 slices, 1.2-mm in-plane resolution, matrix 160 × 160, FOV 768 × 768 mm^2^. A total of 120 frames for both gradient-echo and spin-echo sequences with these parameters were collected up to 2.5 minutes. After 80 seconds, a bolus of 0.1 mMol/kg of GD-DTPA was injected. The acquisition parameters for post-contrast T1-weighted images were identical to those for pre-contrast T1-weighted images. DTI images were acquired with a spin-echo EPI sequence, using b-values = 0/700 s/mm^2^, 42 directions, TR = 7980 ms, TE = 84 ms, 64 slices, and 1.9 mm isotropic images.

### MRI Analysis

Using Slicer software, the contrast-enhancing (CE) lesion on the post-contrast T1-weighted sequence was outlined using a previously described volumetric approach^7^ and defined as the *CE region of interest (ROI)*, which typically represents the tumor ROI (Supplementary Figure 2). Hemorrhagic, cystic, and necrotic areas were excluded from the tumor ROI. The hyperintense lesion on the FLAIR sequence was outlined and defined as the *total FLAIR ROI*. To obtain the *corrected FLAIR ROI* (hereafter referred to as the *FLAIR ROI*), the CE ROI was subtracted from the total FLAIR ROI on the FLAIR sequence. The FLAIR ROI primarily reflects areas of vasogenic edema but also contains non-enhancing infiltrating tumor. Perfusion and diffusion maps were generated within both the tumor and edema ROI as follows. Blood perfusion maps were generated in nordicICE using DSC data. Post-processing T1- and T2-leakage correction was performed, following initial minimization of T1-shortening effects by using the contrast agent pre-dose from DCE to saturate leaky tissue from BBB breakdown or resection^8^. Normalization of tumor DSC values to contralateral normal-appearing gray and white matter was done to minimize patient-specific variations^9^. rCBV and rCBF were calculated on both spin-echo and gradient-echo sequences. Gradient-echo sequences are sensitive to the magnetic susceptibility effect in both small (radius <10 µm) and large (radius >10 µm) vessel calibers whereas spin-echo sequences selectively detect small-caliber vessels^10^. Thus, the respective rCBV and rCBF parameters will be referred to as “panvascular rCBV and rCBF” (derived from gradient-echo sequences) and “microvascular rCBV and rCBF” (derived from spin-echo sequences). DCE data were processed using an in-house MatLab code to produce K^trans^ maps^11^, based on the 2-parameter Tofts-Kermode model^12^ and a population-based arterial input function as described by Parker *et al*.^13^. The 2-parameter Tofts-Kermode model permits calculation of K^trans^ (the transfer constant for contrast agent transport from the blood plasma into the extravascular extracellular space) and v_e_ (the volume fraction of the extravascular extracellular space). DTI data were processed to calculate ADC values and generate corresponding ADC maps, using DTIFit from the FSL Diffusion Toolbox^14^. For each map, the median value across all image voxels within the ROI was calculated.

In addition, vessel architectural imaging (VAI) analysis was performed on the SE and GE DSC data with an in-house MatLab script as previously described^4,15^ to generate parametric vortex areas and vessel caliber within the tumor and contralateral normal reference tissue (combining both gray and white matter). Using these data, the normalized ratio of tumor-to-reference tissue vortex area and vessel size (“vessel size index”) were calculated. The vortex area is a composite parameter reflecting the voxel-wise relative difference between arteriole-to-venule dominance and their oxygen saturation levels^16^.

### Blood Marker Collection

Peripheral blood samples were collected in EDTA-containing tubes at above specified time points. Plasma samples were separated by centrifugation, aliquoted, and stored at −80 °C until used for ELISA measurements. Circulating levels of VEGF, placental growth factor (PlGF), soluble VEGFR1 (sVEGFR1/FLT-1), and basic fibroblast growth factor (bFGF) were measured using the Human Angiogenesis Panel 1 Kit from Meso-Scale Discovery^17^. sTie-2, stromal cell-derived factor 1α (SDF-1α), carbonic anhydrase IX (CAIX), and angiopoietin2 (Ang-2) were measured using ELISA kits from R&D Systems. Plasma samples were run in duplicate.

### Testing for Molecular Markers

Testing for the isocitrate dehydrogenase (*IDH*) mutation was performed using formalin-fixed paraffin-embedded (FFPE) tissue and the IDH1 R132H antibody, which detects the most common *IDH* mutation (*IDH1* variant R132H)^18^. Tumor genotyping, including for non-canonical *IDH* mutations, was performed using the SNaPshot methodology^19^, version 3, a multiplexed allele-specific assay to detect somatic mutations in tumor DNA extracted from FFPE samples. O^6^-methylguanine DNA methyltransferase (*MGMT*) promoter methylation status was tested using methylation-specific PCR based on a standardized protocol at the MGH Department of Pathology.

### Statistical Analysis

All statistical analyses were performed in R, version 3.4.2, using the package “survival” (v2.38). To adjust for intrinsic variability in measurements, the two baseline values for each marker were used to calculate the mean baseline value. At each time point, the percent change of the current value of the marker of interest, compared to the mean baseline value, was calculated.

To analyze the correlation between individual biomarkers and clinical outcome, we applied the landmark approach, which fits a separate Cox regression model for each landmark time point. The points for landmarking corresponded to the time points when patients underwent imaging and peripheral blood collection (weeks 1–6 CRT, pre-C1-C6, and post-C6). The rationale for the landmark approach was based on our intention to fit a simple but flexible model to a large set of time-dependent covariates (i.e. we hypothesized that the effects of covariates on clinical outcome would not remain the same over time). As such, the landmark approach estimates the risk of subsequent disease progression/death from the time of landmark, given that the values of time-dependent covariates are fixed to their values at the landmark time point. Since a separate Cox model is fitted for each landmark time point and the effects of covariates may not remain the same, the landmark approach is more robust to deviations from the proportional hazards assumption than a traditional Cox regression model with time-dependent covariates. The landmark approach only includes patients who were still at risk for disease progression/death at any given landmark time point.

To test whether a marker was a predictor for future progression or death, we included as covariates the current percent change from baseline and a summary of previous (historical) values of the percent change in the same marker. The summary statistic for the historical value was chosen so that it would represent the history of possibly poor response up to the current time point (landmark). At each further landmark the history was updated. For ADC and sVEGFR1, the minimum percent change observed in previous measurements was defined as a “poor response” variable, given that lower ADC values typically reflect increased tumor cellularity^20^ and the role of sVEGFR1 in blocking VEGF and PlGF and contributing to vascular normalization^21^. For all other markers, the maximum percent change in previous measurements was considered a “poor response” variable. We considered models without historical change as well but the models that included historical summary seemed more biologically compelling by reflecting the dynamic evolution of tumor over time. The hazard ratio (HR) represents the factor by which the instantaneous risk of disease progression/death changes with every 1% increase in the percent change of the marker compared to its baseline. Cox regression models were used (function “coxph”) to assess the association of imaging markers, blood markers, and clinical variables (age, *MGMT* promoter methylation status, *IDH* status, and Karnofsky performance status (KPS)) with survival endpoints (PFS and OS). For each imaging marker, four Cox regression models (one each for the MRI parameter of interest within the CE and FLAIR volume and PFS and OS) were fitted. For each blood marker, two Cox regression models (one each for PFS and OS) were fitted. We estimated hazard ratios for three covariates: (1) age at diagnosis, (2) current percent change in a marker, and (3) historical percent change in a marker. In addition, two Cox regression models (one each for PFS and OS) were fitted for mean baseline values of each imaging and blood marker. To assess any correlations between blood and imaging markers, Spearman’s rho correlation coefficient was calculated. In order to account for the large number of models we fitted to the data (13 landmark analyses for each outcome and each marker), we applied the Bonferroni adjustment and used 0.05/13 = 0.004 as the significance level. We considered findings with p-values between 0.004 and 0.05 to be suggestive of an association and worthy of further exploration.

## Results

### Clinical Characteristics

Fourteen patients with histologically confirmed GBM were enrolled between November 2008 and August 2011. Some data from this cohort were previously included in a study comparing MRI and blood marker changes to patients treated with cediranib^4^ and evaluating treatment-related structural and volumetric brain changes^22^. Patient characteristics are summarized in Table 1. Of ten patients with sufficient tissue for testing, *MGMT* promoter methylation was found in two cases. Eight patients had available *IDH1* testing and were all found to be *IDH1*-wild-type (wt) by genetic sequencing (n = 7) or immunohistochemistry (n = 1). In the remaining six patients, *IDH* testing could not be performed due to lack of available tissue or because testing was not yet routinely performed at our institution prior to the discovery of the prognostic significance of the *IDH* mutation. Determination of an association between *MGMT* and *IDH* status and PFS or OS did not yield conclusive results, given the lack of variability in these markers across patients. Median KPS was 90 (range 60–100). No association between KPS and PFS (HR = 1.003, 95% CI 0.939–1.071, *p* = 0.931) or OS (HR = 0.972, 95% CI 0.913–1.036, *p* = 0.38) was detected.

**Table 1:** Patient characteristics.

Patient	Gender	Age	KPS	Surgery	*MGMT* methylation status	*IDH* status*	Steroid requirement during CRT	Post-CRT treatment**	PFS (months)	Salvage treatment	OS (months)
1	M	67	90	STR	Unmethylated	N/A	None	TMZ (4C^)	7.5	REGAL; lomustine; BEV + irinotecan	16.4
2	M	53	100	STR	Unmethylated	wt	Tapered	TMZ (11 C)	14.7	XL184; BEV	25.7
3	M	56	90	STR	Methylated	N/A	Tapered	TMZ + BEV	33.2	Vaccine trial, BEV, ddTMZ; etoposide + BEV, SRS	38.1
4	F	65	90	STR	Unmethylated	N/A	Increased	TMZ (1 C)	4.3	None	5.3
5	F	55	90	Bx	N/A	N/A	Tapered	TMZ (12 C) + VEGF-TRAP	19.4	Cediranib, cilengitide; BEV + TMZ; BEV + CCNU	28.3
6	F	62	100	STR	Unmethylated	wt	Tapered	ddTMZ (5 C)	9.3	None	16.4
7	F	78	60	STR	Methylated	N/A	Stable dose	TMZ (2 days of 1 C)	3.4	None	4.3
8	F	55	100	Bx	Unmethylated	wt	Tapered	TMZ (3 C)	7.1	BEV + ddTMZ; BEV + standard-schedule TMZ; BEV alone	30.3
9	M	58	100	Bx	N/A	N/A	Increased	TMZ (1 C)	3.8	BEV + TMZ	8.7
10	F	69	90	Bx	N/A	wt	Increased	TMZ (5 C)	8.4	None	8.5
11	M	40	90	STR	Unmethylated	wt	Tapered	TMZ (2 C)	5.1	Cediranib, cilengitide; ddTMZ; ddTMZ + BEV	12.2
12	M	35	100	STR	N/A	wt	None	TMZ (7 C)	9.8	Bosutinib; Zactima, sirolimus	15.1
13	F	70	90	STR	Unmethylated	wt	None	TMZ (1 C)	8.8	BEV	14.6
14	F	62	70	STR	Unmethylated	wt	Increased	TMZ (10 C)	12.7	BEV	15.6

*IDH testing was not performed in some patients (“N/A”) either due to lack of sufficient tissue or because it was not incorporated in routine testing at the time. **After completion of concurrent radiation therapy and TMZ. ^Number in brackets denotes the number of cycles of adjuvant temozolomide. Abbreviations: M = male, F = female, STR = subtotal resection, Bx = biopsy, N/A = not available, wt = wild-type, ddTMZ = dose-dense temozolomide, REGAL trial = randomized, phase III trial of cediranib, either as monotherapy or with lomustine, vs. lomustine monotherapy, BEV = bevacizumab, SRS = stereotactic radiosurgery.

All patients completed six weeks of concurrent involved-field radiation therapy (RT) (60 Gy) and TMZ except for Patient 7 and 13 who stopped concurrent TMZ 14 and 2 days before completion of CRT, respectively, due to grade 3 thrombocytopenia. Except for one individual (Patient 6) who received dose-dense TMZ (21 days on/7 days off), all patients received standard-schedule TMZ (5 days on/23 days off). Two patients who started concurrent VEGF inhibitors – bevacizumab (Patient 3) and aflibercept (Patient 5) – following CRT were excluded from the analysis in the post-CRT setting, given the known modulating effects of VEGF inhibitors on MR imaging^3^ and on the levels of blood markers of angiogenesis^4^. Corticosteroid requirements varied during CRT: six patients were tapered off corticosteroids, four required higher doses, and three did not require any at all.

Median PFS was 9.4 months (95% CI 7.7–21.1 months). All patients experienced disease progression based on RANO criteria. Treatment modalities at progression are summarized in Table 1. Median OS was 16.7 months (95% CI 13.3–32.9 months). All patients had died at the time of manuscript submission.

### Imaging Data

A total of 153 MRI exams were performed. The number of patients who underwent MR imaging was mostly consistent up to the pre-C2 time point (Supplementary Table 1). Thereafter, patients began dropping out of the study due to disease progression. The results of the statistical models we fitted can be found in the supplementary materials (Supplementary Tables 2–5), with the most informative associations between imaging markers and survival endpoints shown in Tables 2 and 3.

**Table 2:** Cox regression analysis for progression-free (PFS) and overall survival (OS) using age and baseline values of imaging markers within the edema (FLAIR) volume as co-variates. Baseline values of ADC and K^trans^ were similar between patients so the HR could not be estimated for these markers.

Co-variate	PFS				OS			
	No. events	HR	95% CI	P value	No. events	HR	95% CI	P value
Age	14	1.043	0.966–1.110	0.343	14	1.061	0.965–1.167	0.221
FLAIR volume	14	1.000	1.000–1.000	0.844	14	1.000	1.000-1.000	0.602
Age	14	1.076	0.999–1.159	0.052	14	1.049	0.976–1.128	0.190
Micro rCBV	14	4.366	0.962–19.818	0.056	14	2.067	0.539–7.920	0.290
Age	14	1.091	1.006–1.184	**0.035**	14	1.054	0.975–1.138	0.185
Micro rCBF	14	7.849	1.054–58.473	**0.044**	14	1.869	0.304–11.493	0.500
Age	14	1.093	1.009–1.185	**0.030**	14	1.055	0.982–1.133	0.140
Pan rCBV	14	3.763	1.117–12.674	**0.032**	14	1.965	0.671–5.756	0.218
Age	14	1.096	1.006–1.193	**0.036**	14	1.058	0.983–1.140	0.135
Pan rCBF	14	3.984	1.005–15.803	**0.049**	14	1.993	0.583–6.819	0.272

*p*-values suggestive of an association (<0.05) are bolded although cannot be treated as statistically significant in light of the number of fitted models. No. events = number of events. Micro = microvascular. Pan = panvascular.

**Table 3:** Cox regression analysis using age, current percent change in imaging marker, and historical percent change in imaging marker as co-variates and their correlation with progression-free survival (PFS) and overall survival (OS).

Co-variate	Time point	HR	95% CI	P value	
**Correlation with PFS**					
***Within edema (FLAIR) ROI***					
Age	Pre-C1	1.110	1.009–1.221		**0.032**
Current ADC		1.079	1.011–1.152		**0.022**
Historical ADC		0.971	0.939–1.004		0.081
Age	Pre-C3	1.135	0.994–1.297		0.061
Current ADC		1.257	1.017–1.554		**0.034**
Historical ADC		0.850	0.724–0.997		**0.046**
***Within tumor (CE) ROI***					
Age	Week 2 CRT	0.946	0.866–1.034		0.224
Current K^trans^		1.101	1.014–1.195		**0.021**
Historical K^trans^		0.899	0.821–0.986		0.024
Age	Week 3 CRT	1.047	0.948–1.156		0.365
Current micro rCBF		0.953	0.908–1.000		0.050
Historical micro rCBF		1.078	1.001–1.161		**0.046**
Age	Week 5 CRT	1.060	0.976–1.150		0.165
Current K^trans^		1.038	1.013–1.064		0.003*
Historical K^trans^		0.945	0.909–0.983		**0.005**
Age	Pre-C1	1.144	1.037–1.263		**0.008**
Current K^trans^		1.029	1.009–1.050		0.004*
Historical K^trans^		0.991	0.981–1.000		0.054
**Correlation with OS**					
***Within edema (FLAIR) ROI***					
Age	Week 3 CRT	1.012	0.936–1.094		0.765
Current micro rCBV		0.940	0.892–0.989		**0.018**
Historical micro rCBV		1.044	1.006–1.084		**0.024**
***Within tumor (CE) ROI***					
Age	Week 2 CRT	0.994	0.924–1.070		0.874
Current pan rCBV		0.945	0.886–1.007		0.082
Historical pan rCBV		1.115	1.006–1.235		**0.038**
Age	Week 2 CRT	0.979	0.904–1.059		0.592
Current pan rCBF		0.957	0.909–1.007		0.089
Historical pan rCBF		1.076	1.003–1.156		**0.042**
Age	Week 4 CRT	1.059	0.970–1.157		0.200
Current K^trans^		0.975	0.951–1.000		**0.049**
Historical K^trans^		1.040	0.999–1.083		0.059
Age	Pre-C1	1.079	1.002–1.163		**0.045**
Current K^trans^		1.015	1.002–1.028		**0.027**
Historical K^trans^		0.992	0.981–1.003		0.146
Age	Pre-C1	1.087	0.997–1.186		0.059
Current pan rCBV		0.956	0.917–0.997		**0.037**
Historical pan rCBV		1.048	0.999–1.098		0.055
Age	Pre-C1	1.079	0.988–1.178		0.089
Current pan rCBF		0.962	0.930–0.996		**0.028**
Historical pan rCBF		1.037	1.002–1.072		**0.035**

For clarity, only time points with *p*-values < 0.05 are shown. *p*-values suggestive of an association (>0.004 and <0.05) are bolded. *p*-values compared to the Bonferroni-adjusted significance level (0.05/13 = 0.004) are bolded and marked with an asterisk. For age, the change in HR corresponds to each 1-year increase in age. For imaging markers, the change in HR corresponds to each 1% change in imaging marker. Micro = microvascular. Pan = panvascular.

**Figure 2:**
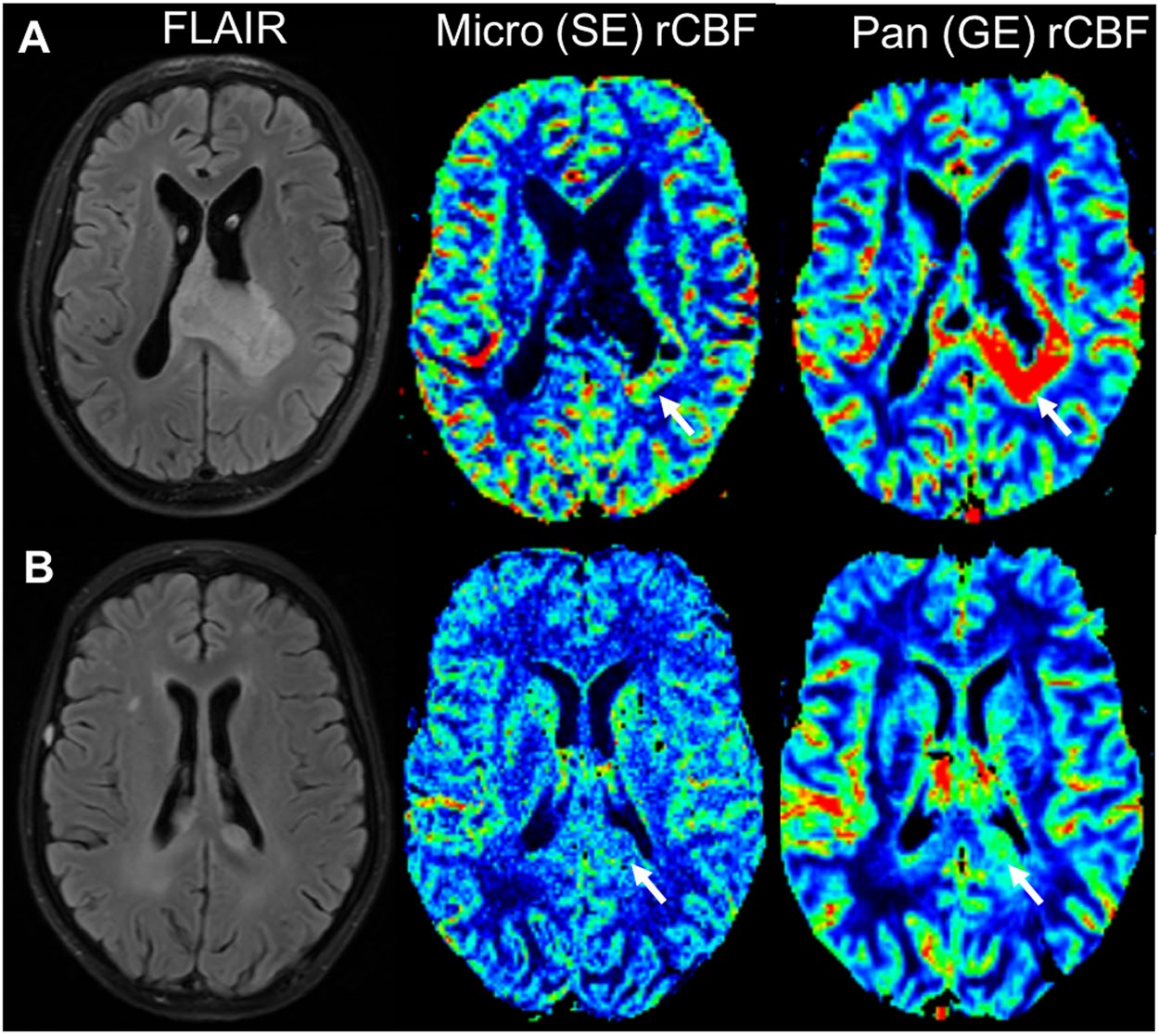
FLAIR and DSC-derived perfusion maps in a patient with below-median (**A**) and above-median (**B**) PFS and OS at baseline. Patient 9 (**A**) had a large FLAIR-hyperintense tumor involving the splenium of the corpus callosum with significantly elevated concomitant rCBF, especially on GE sequences (white arrows). PFS was 3.8 months and the patient died 8.7 months after diagnosis. The tumor in Patient 8 (**B**), on the other hand, only demonstrated minimally elevated rCBF at baseline. PFS and OS were 7.1 and 30.3 months, respectively.

**Figure 3:**
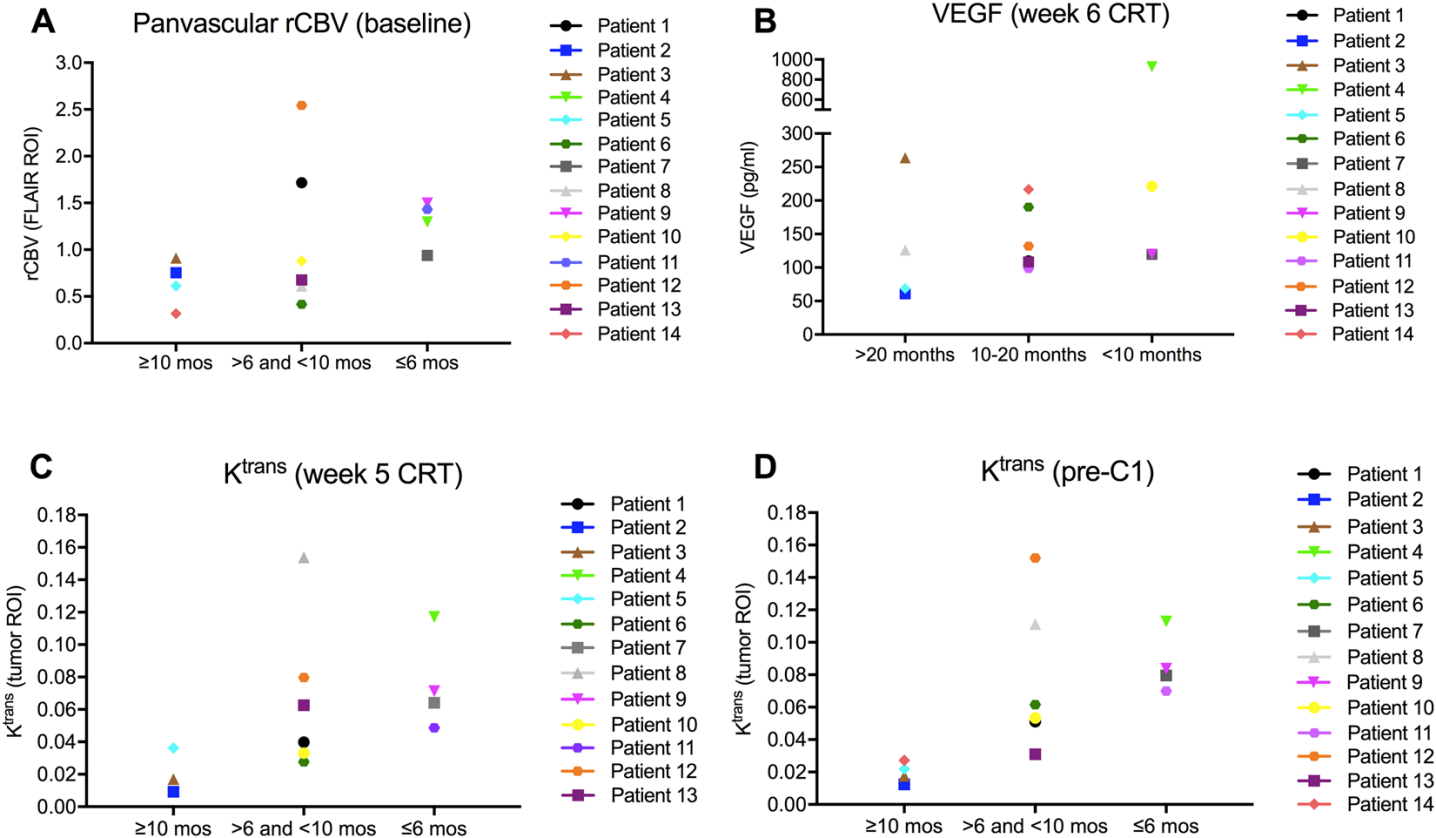
Distribution of baseline panvascular rCBV (derived from gradient-echo sequences) within the edema (FLAIR) ROI (A), VEGF levels at week 6 CRT (B), and K^trans^ at week 5 CRT and pre-C1 (C, D) within the tumor ROI for all patients. For clarity, patients are divided into groups based on duration of PFS (≥10 months, 6–10 months, or ≤6 months) and OS (>20 months, 10–20 months, <10 months). Patients with a PFS ≥10 months generally had low rCBV and K^trans^ values whereas most patients with a PFS ≤6 months had higher rCBV and K^trans^ values. The same applies to low VEGF (seen in patients with an OS >20 months) and high VEGF levels (seen in patients with an OS <10 months).

After controlling for age, we found suggestive associations between higher baseline microvascular rCBF and panvascular rCBV and rCBF within the edema ROI and worse PFS (Table 2; Figs 2 and 3A) although this did not reach statistical significance in light of the number of fitted models. There were no associations between baseline imaging markers and OS.

**Figure 4:**
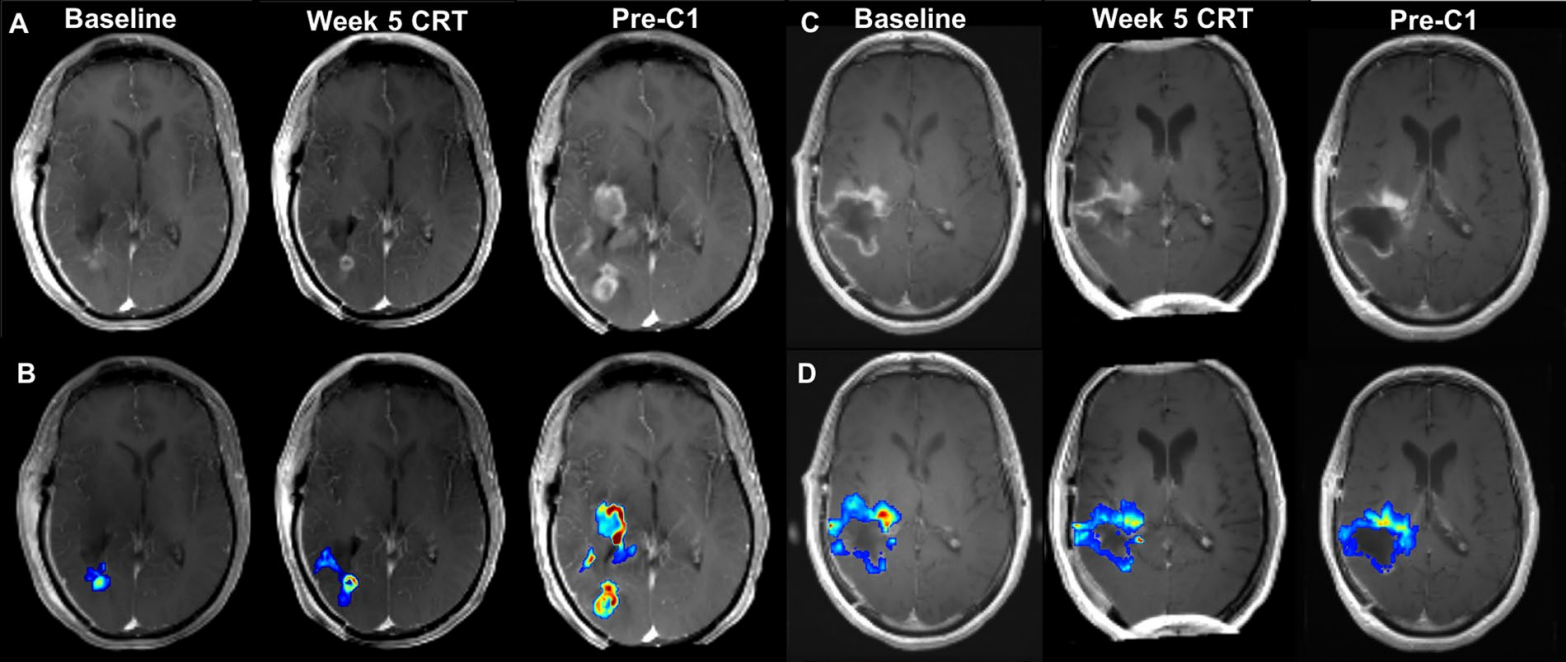
T1 post-contrast sequences and corresponding K^trans^ maps of a patient with below-median (**A,B**) and above-median (**C,D**) PFS and OS at baseline, week 5 of chemoradiation (CRT), and before cycle 1 of TMZ (pre-C1). In Patient 11, there was gradual increase in contrast-enhancing tumor in the right occipital lobe which progressed to involve the right temporal lobe (**A**). The K^trans^ maps demonstrated progressive concomitant increase in permeability (**B**). PFS and OS were 5.1 and 12.2 months, respectively. By contrast, Patient 3 displayed progressive decrease in contrast-enhancing tumor burden in the right frontotemporal lobe during the course of treatment (**C**). K^trans^ maps showed gradual decrease in permeability (**D**). PFS and OS were 33.2 and 38.1 months, respectively.

Within the tumor ROI, high K^trans^ at week 2 (HR = 1.101, *p* = 0.021), week 5 (HR = 1.038, *p* = 0.003), and pre-C1 (HR = 1.029, *p* = 0.004) was associated with an increased risk of later progression (Table 3; Figs 3C,D, and 4; Supplementary Figure 3). After adjusting the significance level for multiple comparisons, the *p*-values remained significant for the week 5 and pre-C1 K^trans^ values. In addition, suggestive associations between multiple imaging markers within the edema and tumor ROIs and clinical outcome were found. These are summarized in Table 3. There were no statistically significant associations between current percent change in levels of the VAI-based parameters (vortex area or vessel size index) and survival endpoints.

In addition to suggestive associations between *current* percent changes and survival endpoints, there were suggestive associations between *historical* percent changes and PFS and/or OS (Table 3). For instance, high historical changes in pre-C3 ADC within the edema ROI (HR = 0.850, *p* = 0.046) and week 5 K^trans^ within the tumor ROI (HR = 0.945, *p* = 0.005) were associated with improved PFS.

### Blood Marker Data

We observed suggestive associations between multiple blood markers and survival. During the first half of CRT (weeks 2 and 3), an increase in the current percent change in SDF-1α (HR = 0.882, *p* = 0.025), IL-8 (HR = 0.965, *p* = 0.025), and VEGF (HR = 0.986, *p* = 0.043) was associated with improved OS (Table 4). During the second half of CRT, the opposite was observed for SDF-1α (at weeks 5 and 6) and VEGF (at week 6): an increase in these markers was associated with worse PFS and OS (Table 4; Fig. 3B; Supplementary Figure 4). This association remained significant for plasma VEGF at week 6 after adjusting for multiple comparisons (Fig. 3B). However, a higher historical percent change in week 6 plasma SDF-1α and VEGF was associated with improved PFS and OS. Similar associations between current and historical percent changes and OS were observed for week 5 plasma Ang-2 levels. In addition, higher week 5 plasma CAIX was associated with improved PFS (HR = 0.973, *p* = 0.012) while higher week 5 plasma sTie-2 predicted worse OS (HR = 1.068, *p* = 0.049). High pre-C2 plasma TNF-α levels were associated with a lower risk of later disease progression (HR = 0.946, *p* = 0.029). A summary of significant associations is shown in Table 4.

**Table 4:** Cox regression analysis using age, current percent change in blood marker, and historical percent change in blood marker as co-variates and their correlation with progression-free survival (PFS) and overall survival (OS).

Co-variate	Time point	HR	95% CI	P value
**Correlation with PFS**				
Age	Week 5 CRT	1.032	0.969–1.099	0.331
Current CAIX		0.973	0.953–0.994	**0.012**
Historical CAIX		1.015	0.999–1.030	0.060
Age	Week 6 CRT	1.052	0.982–1.126	0.152
Current SDF-1α		1.078	1.014–1.147	**0.017**
Historical SDF-1α		0.908	0.838–0.983	**0.017**
Age	Week 6 CRT	1.049	0.978–1.127	0.183
Current VEGF		1.018	1.002–1.035	**0.030**
Historical VEGF		0.978	0.958–0.998	**0.030**
Age	Pre-C2	1.025	0.960–1.094	0.455
Current SDF-1α		0.958	0.916–1.003	0.066
Historical SDF-1α		1.030	1.003–1.058	**0.029**
Age	Pre-C2	0.967	0.895–1.045	0.402
Current TNF-α		0.946	0.900–0.994	**0.029**
Historical TNF-α		1.014	1.003–1.026	**0.016**
Age	Pre-C3	0.888	0.759–1.038	0.135
Current SDF-1α		0.907	0.823–1.000	0.051
Historical SDF-1α		1.039	1.001–1.079	**0.044**
**Correlation with OS**				
Age	Week 2 CRT	1.025	0.945–1.110	0.555
Current Ang-2		1.047	0.999–1.097	0.055
Historical Ang-2		0.907	0.830–0.990	**0.029**
Age	Week 2 CRT	0.970	0.905–1.040	0.394
Current SDF-1α		0.882	0.791–0.984	**0.025**
Historical SDF-1α		1.034	0.952–1.122	0.427
Age	Week 3 CRT	1.019	0.952–1.090	0.592
Current IL-8		0.965	0.935–0.995	**0.025**
Historical IL-8		1.019	0.998–1.039	0.073
Age	Week 3 CRT	1.030	0.961–1.103	0.401
Current VEGF		0.986	0.972–1.000	**0.043**
Historical VEGF		1.004	0.988–1.021	0.599
Age	Week 4 CRT	0.996	0.925–1.072	0.914
Current CAIX		1.021	0.994–1.049	0.131
Historical CAIX		0.969	0.941–0.997	**0.033**
Age	Week 5 CRT	1.045	0.959–1.140	0.314
Current Ang-2		1.066	1.016–1.117	**0.009**
Historical Ang-2		0.941	0.900–0.983	**0.007**
Age	Week 5 CRT	1.030	0.950–1.117	0.476
Current hTie-2		1.068	1.000–1.140	**0.049**
Historical hTie-2		0.897	0.803–1.002	0.054
Age	Week 5 CRT	1.007	0.939–1.079	0.849
Current SDF-1α		1.050	1.005–1.098	**0.030**
Historical SDF-1α		0.896	0.813–0.987	**0.026**
Age	Week 5 CRT	1.038	0.962–1.120	0.336
Current VEGF		1.009	0.995–1.022	0.216
Historical VEGF		0.985	0.971–1.000	**0.044**
Age	Week 6 CRT	1.049	0.979–1.125	0.177
Current SDF-1α		1.074	1.014–1.137	**0.014**
Historical SDF-1α		0.890	0.818–0.968	**0.006**
Age	Week 6 CRT	1.070	0.987–1.160	0.099
Current VEGF		1.034	1.011–1.058	0.004*
Historical VEGF		0.961	0.935–0.989	**0.006**
Age	Pre-C1	1.041	0.967–1.121	0.284
Current CAIX		1.007	0.995–1.019	0.275
Historical CAIX		0.983	0.969–0.997	**0.016**
Age	Pre-C1	1.073	0.983–1.171	0.114
Current SDF-1α		1.040	0.991–1.092	0.113
Historical SDF-1α		0.965	0.932–0.999	**0.046**

For clarity, only time points with *p*-values < 0.05 are shown. *p*-values suggestive of an association (>0.004 and <0.05) are bolded. *p*-values compared to the Bonferroni-adjusted significance level (0.05/13 = 0.004) are bolded and marked with an asterisk. For age, the change in HR corresponds to each 1-year increase in age. For blood marker, the change in HR corresponds to each 1% change in blood marker.

### Correlation between imaging and blood markers

We evaluated whether imaging markers that demonstrated a significant association with clinical outcome at any given time also correlated with blood markers at the same time point. A correlation coefficient, ρ, >0.7 was considered to indicate a high correlation. We did not find any significant correlations between imaging and blood markers (Supplementary Table 6).

## Discussion

In this prospective trial evaluating 153 MRI scans in newly diagnosed GBM patients treated with standard CRT and adjuvant TMZ, we performed an in-depth analysis to determine if certain MR imaging and peripheral blood markers at different treatment time points predicted clinical outcome. Our study revealed 1) a suggestive association between high baseline/pre-treatment microvascular rCBF and panvascular rCBV and rCBF within the edema ROI and an increased risk of disease progression; 2) a statistically significant association between high week 5 and pre-C1 K^trans^ values in the tumor ROI and worse PFS; and 3) a statistically significant association between high week 6 VEGF levels and worse OS. Given that these markers are measures of blood perfusion and angiogenesis, our findings suggest a significant impact of chemoradiation on the vascular state in both the tumor and edema regions, and an important modulatory effect of vascular physiology on treatment response.

There is a known association between elevated rCBV and the degree of tumor malignancy and therapy response, given the increase in capillary density in the setting of tumor angiogenesis^23^ Our finding that pre-treatment micro- and panvascular rCBV and rCBF within the edema ROI were associated with worse PFS is in line with previous reports. Multiple studies have demonstrated worse OS and PFS in glioma patients with elevated pre-surgical rCBV in the enhancing tumor ROI^24–33^. Higher baseline rCBV in the non-enhancing edema ROI has also been linked to worse survival^34^, presumably because this region contains infiltrating tumor cells necessitating a richer blood supply and eventually transforming into a more aggressive phenotype^26^. A major challenge with DSC MRI is the current lack of standardization of acquisition and post-processing techniques, which may particularly affect measurement of rCBF. Our finding that DSC MRI helps probe vascular state may therefore provide an additional incentive for standardization in future brain tumor studies.

While there is consensus that high pre-treatment rCBV and rCBF values are associated with worse outcome, the impact of perfusion on survival *during* and *after* treatment is less clear. This may be related to the heterogeneity of patients (anaplastic gliomas, GBMs, and both), treatment modalities (anti-angiogenic treatment versus no anti-angiogenic treatment), and time points of measurement (newly diagnosed versus recurrent setting) in previous studies^4,35–38^. Some data in GBM patients have suggested that elevated panvascular rCBV one month after CRT is associated with worse survival^37^. By contrast, others have found that elevated microvascular rCBV within 3 months of completing standard therapy predicted improved PFS^38^. In the setting of anti-angiogenic therapy, our group has shown that improvement (i.e. an increase) of sub-normal perfusion after cediranib treatment predicted improved OS in the newly diagnosed^4^ and recurrent setting^8^. This was likely a reflection of cediranib-induced vascular normalization, improvement in drug and oxygen delivery, and a reduction in the immunosuppressive effects of hypoxia and acidosis on the tumor microenvironment^39^. Others have shown the opposite effects with other types of anti-angiogenic agents; higher rCBV 3–8.5 weeks after bevacizumab in recurrent high-grade glioma patients was associated with worse OS^35^. Interestingly, lower rCBV at week 2 and 16 after bevacizumab appeared to predict better OS^36^. Collectively, these data suggest that the pre-therapy levels and subsequent time points at which perfusion are measured are important, given the highly complex and dynamic behavior of tumor angiogenesis. There may also be differing effects on vascular physiology of antibodies like bevacizumab and tyrosine kinase inhibitors (such as cediranib). Although it did not reach the Bonferroni-adjusted significance level, our results suggest that elevated week 3 microvascular rCBV in the edema ROI may be associated with improved OS, supporting the hypothesis that a functional vasculature favorably modulates treatment response. We also observed a positive correlation between elevated post-radiation (pre-C1) panvascular rCBV and rCBF and OS, which may be explained by some residual beneficial effect of radiation on the tumor vasculature, reversal of hypoxia, and improved drug delivery that persisted after completion of RT. These findings should be investigated in larger studies using standardized DSC protocols.

K^trans^, the volume transfer constant, is derived from DCE MRI. In the setting of low vascular permeability (i.e. a near-intact BBB), K^trans^ reflects permeability. Under conditions of high permeability (i.e. a disrupted BBB as seen in GBMs), K^trans^ also reflects blood flow^40^. In theory, perfusion could be high while permeability is low, and vice versa. In this study, we observed opposite directional changes after chemoradiation (pre-C1): high K^trans^ was associated with *worse* OS whereas high panvascular rCBV and rCBF were associated with *improved* OS. Notably, in the setting of highly vascularized tissue such as GBM, the use of an extended 3-parameter model^40^ that provides information on v_p_ (the total blood plasma volume) – in addition to the parameters K^trans^ and v_e_ – may facilitate assessment of the impact of blood flow on the interpretation of K^trans^ in terms of flow and permeability weighting. In this study, we chose to use the 2-parameter model after finding that the 3-parameter model was less repeatable than the 2-parameter model at a temporal resolution of 6 seconds (manuscript in preparation). Thus, since the 2-parameter model regards the capillary plasma volume as negligible (i.e. assumes weakly vascularized tissue) and trans-endothelial leakage as low, it is difficult to draw conclusions on the kinetics and quantification of contrast agent accumulation within the tumor from this data set.

The intuitive assumption is that high K^trans^ is associated with worse outcome, given that neovascularization and higher vascular permeability are hallmarks of high-grade tumors. This has been corroborated by some reports, which suggested a relationship between high pre-surgical K^trans^ and worse PFS/OS^41–43^. However, others did not find such an association^26,44,45^. As with rCBV and rCBF, these divergent findings may be due to the inclusion of a heterogeneous patient population, different imaging acquisition protocols and post-processing techniques, and the above-mentioned complexity in distinguishing blood flow from permeability when K^trans^ is calculated.

Only a handful of prospective studies have addressed the significance of K^trans^ changes *during* treatment. While one study did not find an association between K^trans^ values during treatment and clinical outcome^46^, another study demonstrated an association between a decrease in K^trans^ after week 3 of CRT and treatment response^47^. Our data suggest a probable association between higher K^trans^ during week 4 of CRT and improved OS, possibly due to improved blood flow and resultant oxygenation and drug delivery to the tumor. As mentioned above, application of the 3-parameter extended Tofts model may help shed light on this. Although this association did not reach the Bonferroni-corrected significance level, it underscores the notion of a potential “critical window of opportunity” during which increased permeability is beneficial.

We found that higher levels of VEGF - a marker of angiogenesis - during week 6 of CRT were associated with worse OS. VEGF has been explored extensively as a biomarker of response, primarily in the setting of anti-angiogenic therapy. Most studies have not shown a correlation between VEGF levels and clinical outcome^4,48^. Given that angiogenesis and its reversal is a highly dynamic process on both a temporal and spatial level, the timing of VEGF measurements is likely critical. Our findings of an association between high week 6 VEGF levels and worse survival will require further investigation in a larger and ideally randomized patient cohort but suggest that ongoing angiogenesis reflects a more aggressive tumor microenvironment.

We also observed suggestive associations between increased Ang-2, sTie-2, and SDF-1α levels during late radiation (week 5/6) and worse outcome. To date, no study has established a prognostic role for Ang-2 and sTie-2 on survival^17,49^. SDF-1α, a pro-inflammatory chemo-attractant cytokine for macrophages, has been shown to play a role in the restoration of tumor vasculature after irradiation of GBM cell lines and to promote radiation-induced cell invasion^50^. In line with this, SDF-1α suppression by siRNA^51^ and SDF-1α inhibitors^52^ resulted in decreased tumor invasiveness and prolonged survival. Interestingly, higher plasma VEGF, IL-8, and SDF-1α levels early during radiation (week 2/3) were associated with improved survival. One possible explanation is that radiation might initially create a tumor immune microenvironment that promotes anti-tumor immune responses, perhaps via accumulation of macrophages and dendritic cells that facilitate lymphocyte activation. The lack of durability of this response might be related to the high sensitivity of lymphocytes to irradiation and predominance of immunosuppressive factors after prolonged treatment. Further studies in larger patient cohorts may help confirm these hypotheses, and could have important implications for the use of immunotherapy in GBM patients.

Our study has some limitations. First, our sample size was small and therefore limits our ability to derive generalized conclusions. In addition, it was not possible from a statistical standpoint to include *MGMT* promoter methylation and *IDH* status in our Cox regression model or impute missing values, given that most or all patients with available *MGMT* and *IDH* status belonged to one group only (i.e. were *MGMT* promoter-unmethylated (8 of 10 patients) or *IDH*-wild-type (8 of 8 patients)). Of the six patients without available *IDH* status, all were age ≥55 years at the time of diagnosis. Recent reports suggest that <7% of patients age ≥55 years have *IDH* mutations on IHC^53,54^ and an even smaller proportion (<1%) of patients in this age group will have a non-canonical mutation on sequencing after negative IHC^54^. Given the age criterion, there is a high likelihood that the patients who did not undergo *IDH* testing in our cohort were *IDH*-wild-type, rather than *IDH*-mutant. Second, for each time point, we assessed the percent change in the marker of interest compared to baseline, rather than the change in absolute value. Consequently, some results may appear statistically significant even if the absolute change may not be clinically meaningful or reflect an actual change in tumor biology. In fact, some of the significant HRs we observed were in the single-digit range. Third, we fitted a large number of models to our data which can generate significant *p* values simply by chance. To account for this possibility, we adjusted our significance level to 0.004 after which only week 5 and pre-C1 K^trans^ values and week 6 VEGF levels remained significant. Lastly, we used ROI-based median values to assess percent changes over time which may not adequately capture the spatial heterogeneity of GBMs, particularly with respect to regional perfusion. One way to assess regional changes is by means of parametric response maps^55^, which are based on voxel-by-voxel comparisons of perfusion or diffusion maps over time^55,56^. However, these studies require highly precise image registration and typically exclude voxels that are not present in both the baseline and intra-treatment tumor volumes^55^. Both these factors thus make it difficult to implement this technique if large changes in tumor volume occur between serial MRIs as can happen during CRT.

Despite these limitations, our study also has clear methodological strengths. Patients were enrolled prospectively and received the same type of treatment during CRT. Notwithstanding our small cohort size, we were able to obtain MRI and blood marker data for almost all patients up to the pre-C2 time point, making this the first study to acquire these data on a weekly basis during CRT. Furthermore, unlike earlier studies which focused primarily on the enhancing tumor ROI^46,47^, we also analyzed the non-enhancing FLAIR ROI. This is important since it is well established that the enhancing tumor ROI does not capture the full extent of infiltrating tumor volume. Lastly, although only a few markers remained significant after correcting for multiple comparisons, we also found multiple suggestive associations between imaging and blood markers and clinical outcome (*p*-values > 0.004 and <0.05). These results may be hypothesis-generating and could be further explored in future studies.

In summary, our study demonstrates that multiple vascular imaging and blood markers, including K^trans^, rCBV, rCBF, and VEGF, undergo significant changes during chemoradiation, highlighting their value as potential markers of response at different time points during the treatment course. Validation of these findings in a larger, multi-institutional patient cohort and with the use of standardized cross-institutional acquisition and post-processing protocols for DSC and DCE MRI will be required. Eventually, these perfusion-weighted imaging techniques could be implemented into standard clinical practice to identify early treatment responders and to study the impact of novel drugs on tumor physiology.

## Electronic supplementary material

Supplementary figures and tables

Dataset 1_clinical data

Dataset 2_imaging and blood data

Dataset 3_VAI data

## Data Availability

The data analyzed during this study are included in this published article and its Supplementary Information files.
