# Publisher Correction: Probing tumor microenvironment in patients with newly diagnosed glioblastoma during chemoradiation and adjuvant temozolomide with functional MRI

**DOI:** 10.1038/s41598-019-44365-2

**Published:** 2019-06-14

**Authors:** K. Ina Ly, Bella Vakulenko-Lagun, Kyrre E. Emblem, Yangming Ou, Xiao Da, Rebecca A. Betensky, Jayashree Kalpathy-Cramer, Dan G. Duda, Rakesh K. Jain, Andrew S. Chi, Scott R. Plotkin, Tracy T. Batchelor, Gregory Sorensen, Bruce R. Rosen, Elizabeth R. Gerstner

**Affiliations:** 1 Stephen E. and Catherine Pappas Center for Neuro-Oncology, Department of Neurology, Massachusetts General Hospital, Boston, MA USA; 2 Department of Biostatistics, Harvard T.H. Chan School of Public Health, Boston, MA USA; 3 Department of Diagnostic Physics, Division of Radiology and Nuclear Medicine, Oslo University Hospital, Oslo, Norway; 4 Department of Pediatrics and Radiology, Boston Children’s Hospital, Boston, MA USA; 5 Functional Neuroimaging Laboratory, Brigham and Women’s Hospital, Boston, MA USA; 6 Athinoula A. Martinos Center for Biomedical Imaging, Department of Radiology, Massachusetts General Hospital, Charlestown, MA USA; 7 Edwin L. Steele Laboratories, Department of Radiation Oncology, Massachusetts General Hospital, Boston, MA USA; 8 Laura and Isaac Perlmutter Cancer Center, New York University Langone Health, New York, NY USA; 9 IMRIS, Deerfield Imaging, Minnetonka, MN USA

Correction to: *Scientific Reports* 10.1038/s41598-018-34820-x, published online 20 November 2018

In the original version of this Article, the author K. Ina Ly was incorrectly indexed. This error has now been corrected.

