# Supplementary material for: Probing tumor microenvironment in patients with newly diagnosed glioblastoma during chemoradiation and adjuvant temozolomide with functional MRI
Source: Sci Rep. 2018 Nov 20;8:17062. doi: 10.1038/s41598-018-34820-x (PMC6244161; doi:10.1038/s41598-018-34820-x)
Supplement: Supplementary file 1 — Supplementary figures and tables [file 41598_2018_34820_MOESM1_ESM.pdf]

**Supplementary data:**

# Probing tumor microenvironment in patients with newly diagnosed glioblastoma during chemoradiation and adjuvant temozolomide with functional MRI

K. Ina Ly, M.D., Bella Vakulenko-Lagun, Ph.D., Kyrre E. Emblem, Ph.D., Yangming Ou, Ph.D., Xiao Da, M.S., Rebecca A. Betensky, Ph.D., Jayashree Kalpathy-Cramer, Ph.D., Dan G. Duda, D.M.D., Ph.D., Rakesh K. Jain, Ph.D., Andrew S. Chi, M.D., Ph.D., Scott R. Plotkin, M.D., Ph.D., Tracy T. Batchelor, M.D., M.P.H., Gregory Sorensen, M.D., Bruce R. Rosen, M.D., Ph.D., Elizabeth R. Gerstner, M.D.

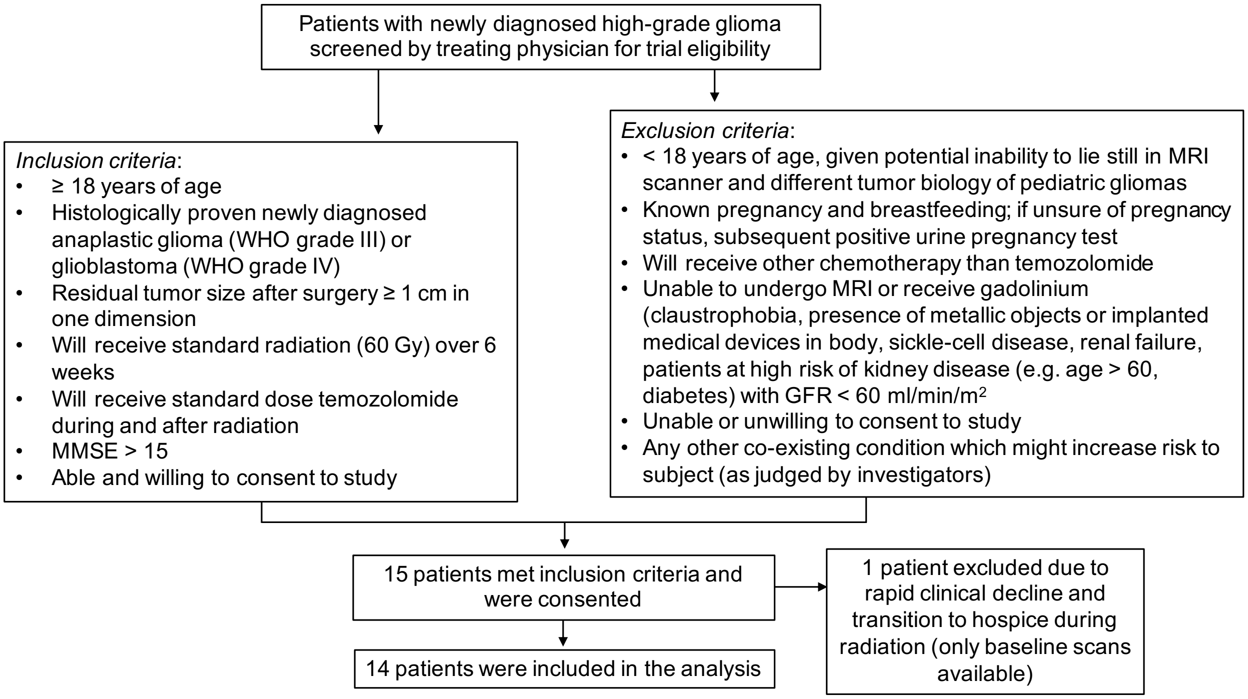


**Supplementary Figure 1**. Overview of patient enrollment and inclusion and exclusion criteria.

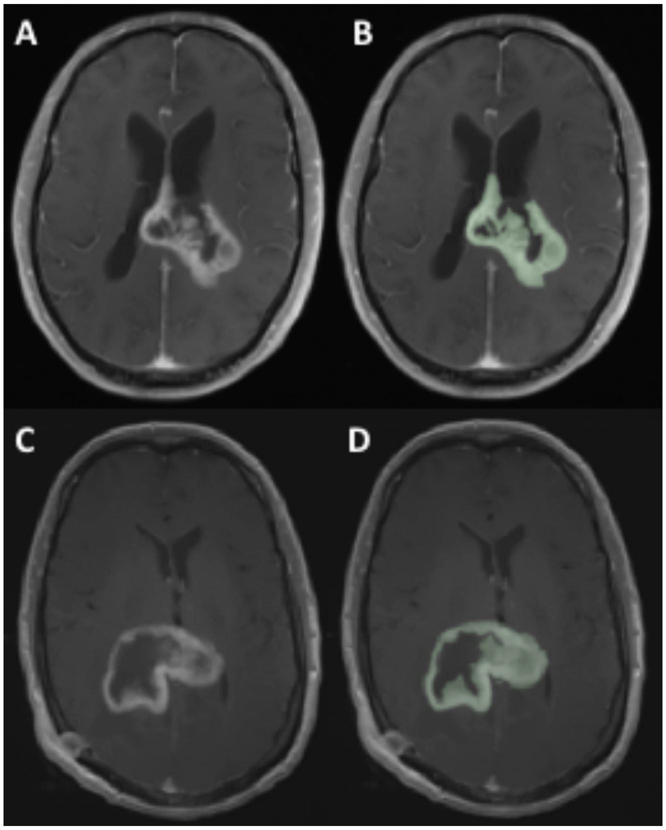


**Supplementary Figure 2**. Examples of tumor regions of interest (ROIs) from Patient 9 (A, B) and Patient 5 (C, D). The contrast-enhancing regions on the T1-weighted post-contrast sequences (A, C) were used to define the tumor ROI, excluding hemorrhagic, cystic, and necrotic areas. The green-shaded masks (B, D) were used for imaging parameter analysis.

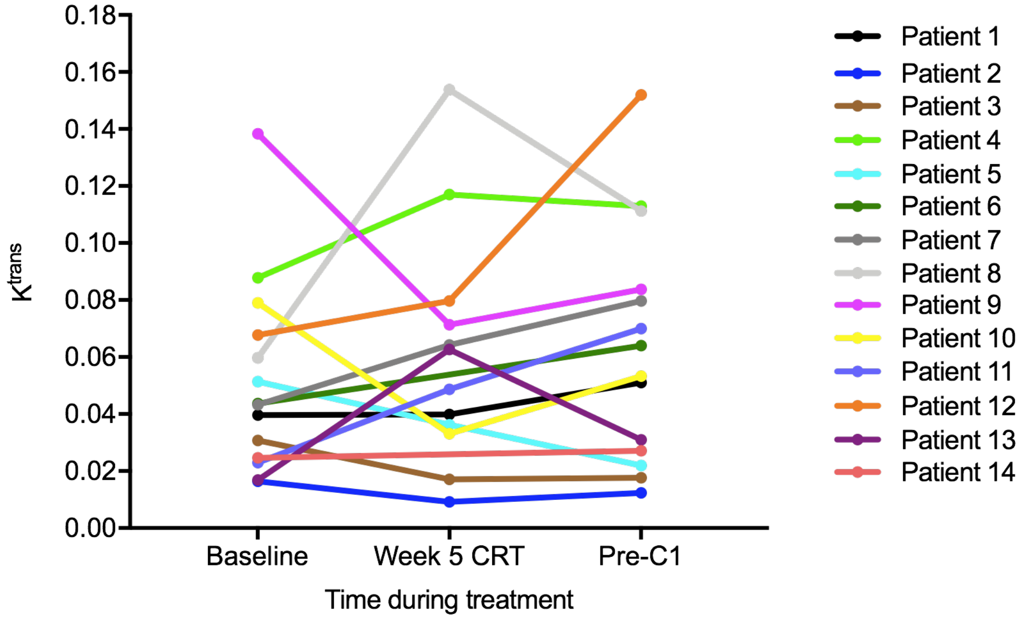


**Supplementary Figure 3**. Changes in $K^{trans}$ within the tumor ROI for all patients during the course of treatment. Only time points at which significant associations between $K^{trans}$ and clinical outcome were found are shown (week 5 chemoradiation and pre-cycle 1 TMZ). For clarity, individual patient data points are connected by solid lines but do not necessarily imply a linear change between the time points. Patients with short PFS (≤ 6 months; e.g. Patients 4, 7, and 11) had a clear increase in $K^{trans}$ at week 5 and pre-C1 compared to baseline. By contrast, 3 of 4 patients with long PFS (≥ 10 months; Patients 2, 3 and 5) had lower $K^{trans}$ values at week 5 and pre-C1 compared to pre-treatment.

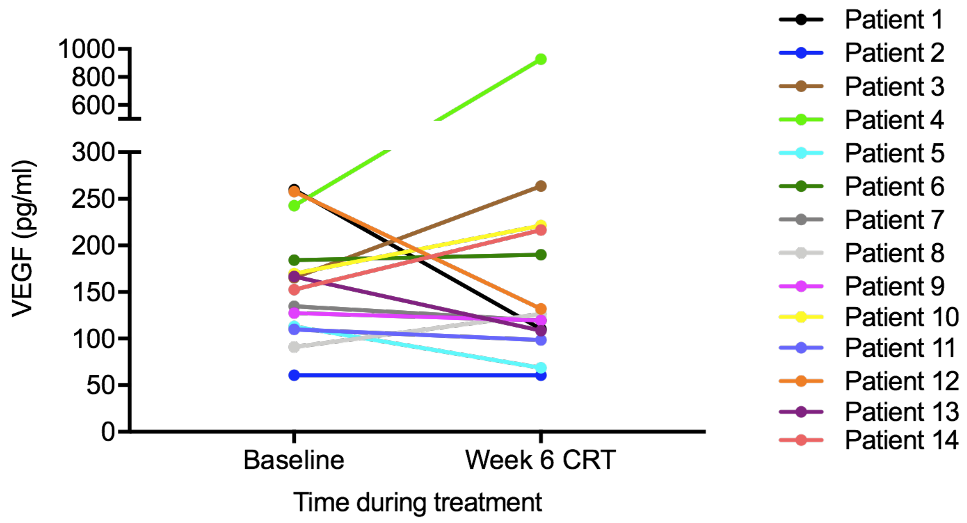


**Supplementary Figure 4**. Changes in plasma VEGF levels for all patients during the course of treatment. Only time points at which significant associations between VEGF and OS were found are shown (week 6 chemoradiation). For clarity, individual patient data points are connected by solid lines but do not necessarily imply a linear change between the time points. For example, Patients 4 and 10 had a significant increase in VEGF at week 6 CRT compared to baseline and only survived for 5.3 and 8.5 months, respectively. By contrast, Patients 1 and 5 demonstrated a decrease in VEGF and had longer survival times of 16.4 and 28.3 months, respectively.

| Study time point | Number of patients with MR imaging | Peripheral blood collection |
|---|---|---|
| BL1 | 14 | 12 |
| BL2 | 13 | 9 |
| Week 1 CRT | 13 | 13 |
| Week 2 CRT | 12 | 12 |
| Week 3 CRT | 13 | 14 |
| Week 4 CRT | 12 | 12 |
| Week 5 CRT | 13 | 14 |
| Week 6 CRT | 11 | 14 |
| Pre-C1 TMZ | 14 | 14 |
| Pre-C2 TMZ | 9 | 9 |
| Pre-C3 TMZ | 9 | 8 |
| Pre-C4 TMZ | 7 | 6 |
| Pre-C5 TMZ | 6 | 5 |
| Pre-C6 TMZ | 4 | 4 |
| Post-C6 | 3 | 3 |

**Supplementary Table 1.** Number of patients who underwent MR imaging and peripheral blood collection at each study time point.

**Supplementary Table 2**. Cox regression analysis using age, current percent change in imaging marker, and historical percent change in imaging marker **within the edema (FLAIR) ROI** as co-variates and their correlation with progression-free (PFS) and overall survival (OS) at each time point during and after treatment. Lack of values and "Inf" (infinity) reflect a lack of convergence of the "coxph" function, which can occur when there are a small number of observations with similar values for a given co-variate. In these cases, p-values cannot be reported ("N/A"). "Micro" = microvascular; derived from spin-echo sequences. "Pan" = panvascular; derived from gradient-echo sequences.

| | | PFS | | | OS | | |
|---|---|---|---|---|---|---|---|
| Co-variate | Timepoint | HR | 95% CI | P-value | HR | 95% CI | P-value |
| Age | Week 2 CRT | 1.017 | 0.946-1.093 | 0.646 | 0.995 | 0.918-1.078 | 0.905 |
| Current ADC | | 1.002 | 0.962-1.044 | 0.911 | 0.971 | 0.927-1.018 | 0.222 |
| Historical ADC | | 1.005 | 0.961-1.051 | 0.826 | 1.010 | 0.964-1.058 | 0.689 |
| Age | Week 3 CRT | 1.068 | 0.955-1.194 | 0.249 | 1.043 | 0.919-1.184 | 0.512 |
| Current ADC | | 1.014 | 0.974-1.057 | 0.492 | 1.004 | 0.967-1.043 | 0.835 |
| Historical ADC | | 0.985 | 0.923-1.052 | 0.661 | 0.970 | 0.908-1.036 | 0.367 |
| Age | Week 4 CRT | 1.032 | 0.942-1.132 | 0.497 | 1.027 | 0.921-1.146 | 0.633 |
| Current ADC | | 1.001 | 0.942-1.063 | 0.985 | 0.983 | 0.927-1.042 | 0.561 |
| Historical ADC | | 1.000 | 0.963-1.037 | 0.984 | 1.001 | 0.965-1.038 | 0.954 |
| Age | Week 5 CRT | 1.012 | 0.919-1.113 | 0.811 | 0.995 | 0.903-1.097 | 0.921 |
| Current ADC | | 0.946 | 0.848-1.056 | 0.324 | 0.935 | 0.841-1.039 | 0.213 |
| Historical ADC | | 1.011 | 0.981-1.041 | 0.476 | 1.005 | 0.977-1.035 | 0.713 |
| Age | Week 6 CRT | 1.034 | 0.953-1.123 | 0.422 | 1.013 | 0.933-1.100 | 0.754 |
| Current ADC | | 1.022 | 0.983-1.064 | 0.276 | 1.032 | 0.979-1.088 | 0.241 |
| Historical ADC | | 0.995 | 0.971-1.020 | 0.693 | 0.986 | 0.961-1.011 | 0.271 |
| Age | Pre-C1 | 1.110 | 1.009-1.221 | 0.032 | 1.109 | 0.988-1.245 | 0.080 |
| Current ADC | | 1.079 | 1.011-1.152 | 0.022 | 1.070 | 0.995-1.150 | 0.069 |
| Historical ADC | | 0.971 | 0.939-1.004 | 0.081 | 0.971 | 0.936-1.007 | 0.109 |
| Age | Pre-C2 | 0.947 | 0.830-1.081 | 0.420 | 0.988 | 0.876-1.114 | 0.846 |
| Current ADC | | 1.121 | 0.989-1.271 | 0.075 | 1.065 | 0.991-1.114 | 0.088 |
| Historical ADC | | 0.951 | 0.893-1.013 | 0.117 | 0.958 | 0.910-1.009 | 0.102 |
| Age | Pre-C3 | 1.135 | 0.994-1.297 | 0.061 | 0.993 | 0.894-1.103 | 0.894 |
| Current ADC | | 1.257 | 1.017-1.554 | 0.034 | 1.095 | 0.938-1.277 | 0.250 |
| Historical ADC | | 0.850 | 0.724-0.997 | 0.046 | 0.909 | 0.796-1.039 | 0.161 |
| Age | Pre-C4 | Inf | 0-Inf | N/A | Inf | 0-Inf | N/A |
| Current ADC | | Inf | 0-Inf | N/A | Inf | 0-Inf | N/A |
| Historical ADC | | Inf | 0-Inf | N/A | Inf | 0=Inf | N/A |
| Age | Pre-C5 | Inf | 0-Inf | N/A | 0.993 | 0.897-1.099 | 0.892 |
| Current ADC | | Inf | 0-Inf | N/A | 1.046 | 0.927-1.180 | 0.465 |
| Historical ADC | | Inf | 0-Inf | N/A | 0.907 | 0.716-1.149 | 0.420 |
| Age | Pre-C6 | Inf | 0-Inf | N/A | Inf | 0-Inf | N/A |
| Current ADC | | Inf | 0-Inf | N/A | Inf | 0-Inf | N/A |
| Historical ADC | | | | | | | |
| Age | Post-C6 | Inf | 0-Inf | N/A | Inf | 0-Inf | N/A |
| Current ADC | | | | | | | |
| Historical ADC | | | | | | | |

| | | PFS | | | OS | | |
|---|---|---|---|---|---|---|---|
| Co-variate | Timepoint | HR | 95% CI | P value | HR | 95% CI | P value |
| Age | Week 2 CRT | 1.012 | 0.944-1.085 | 0.728 | 1.014 | 0.943-1.091 | 0.700 |
| Current micro rCBV | | 1.002 | 0.977-1.028 | 0.861 | 1.007 | 0.944-1.075 | 0.829 |
| Historical micro rCBV | | 0.984 | 0.936-1.043 | 0.524 | 1.000 | 0.926-1.080 | 1.000 |
| Age | Week 3 CRT | 1.050 | 0.969-1.138 | 0.235 | 1.050 | 0.937-1.177 | 0.397 |
| Current micro rCBV | | 0.967 | 0.927-1.008 | 0.111 | 0.983 | 0.957-1.009 | 0.204 |
| Historical micro rCBV | | 1.015 | 0.987-1.043 | 0.297 | 1.030 | 0.990-1.072 | 0.148 |
| Age | Week 4 CRT | 1.018 | 0.919-1.126 | 0.736 | 0.996 | 0.906-1.094 | 0.927 |
| Current micro rCBV | | 0.996 | 0.956-1.038 | 0.853 | 0.995 | 0.955-1.038 | 0.826 |
| Historical micro rCBV | | 0.999 | 0.981-1.017 | 0.887 | 1.014 | 0.967-1.062 | 0.569 |
| Age | Week 5 CRT | 1.045 | 0.965-1.131 | 0.281 | 1.012 | 0.932-1.099 | 0.770 |
| Current micro rCBV | | 0.981 | 0.931-1.033 | 0.468 | 0.991 | 0.946-1.039 | 0.710 |
| Historical micro rCBV | | 0.995 | 0.959-1.031 | 0.771 | 1.014 | 0.969-1.062 | 0.550 |
| Age | Week 6 CRT | 1.040 | 0.963-1.122 | 0.319 | 1.009 | 0.943-1.080 | 0.800 |
| Current micro rCBV | | 0.998 | 0.961-1.037 | 0.93 | 1.003 | 0.968-1.039 | 0.870 |
| Historical micro rCBV | | 0.994 | 0.968-1.021 | 0.646 | 1.009 | 0.978-1.040 | 0.583 |
| Age | Pre-C1 | 1.024 | 0.956-1.097 | 0.494 | 1.039 | 0.957-1.128 | 0.367 |
| Current micro rCBV | | 0.993 | 0.958-1.030 | 0.700 | 0.982 | 0.958-1.006 | 0.135 |
| Historical micro rCBV | | 0.998 | 0.977-1.020 | 0.891 | 1.014 | 0.992-1.036 | 0.222 |
| Age | Pre-C2 | 0.953 | 0.844-1.076 | 0.436 | 0.953 | 0.848-1.072 | 0.423 |
| Current micro rCBV | | 1.067 | 0.986-1.156 | 0.109 | 1.089 | 0.979-1.211 | 0.118 |
| Historical micro rCBV | | 0.956 | 0.909-1.006 | 0.084 | 0.973 | 0.931-1.016 | 0.217 |
| Age | Pre-C3 | 1.014 | 0.936-1.098 | 0.737 | 1.018 | 0.922-1.123 | 0.725 |
| Current micro rCBV | | 1.005 | 0.953-1.059 | 0.868 | 0.978 | 0.885-1.081 | 0.667 |
| Historical micro rCBV | | 0.992 | 0.958-1.028 | 0.657 | 1.012 | 0.975-1.050 | 0.533 |
| Age | Pre-C4 | 1.087 | 0.896-1.317 | 0.397 | 1.148 | 0.786-1.676 | 0.476 |
| Current micro rCBV | | 0.994 | 0.945-1.046 | 0.818 | 0.981 | 0.862-1.117 | 0.775 |
| Historical micro rCBV | | 1.001 | 0.971-1.032 | 0.936 | 0.980 | 0.832-1.154 | 0.811 |
| Age | Pre-C5 | Inf | 0-Inf | N/A | 0.955 | 0.735-1.240 | 0.730 |
| Current micro rCBV | | Inf | 0-Inf | N/A | 1.015 | 0.970-1.063 | 0.517 |
| Historical micro rCBV | | Inf | 0-Inf | N/A | 0.970 | 0.792-1.187 | 0.766 |
| Age | Pre-C6 | Inf | 0-Inf | N/A | Inf | 0-Inf | N/A |
| Current micro rCBV | | Inf | 0-Inf | N/A | Inf | 0-Inf | N/A |
| Historical micro rCBV | | | | | Inf | 0-Inf | N/A |
| Age | Post-C6 | Inf | 0-Inf | N/A | Inf | 0-Inf | N/A |
| Current micro rCBV | | | | | | | |
| Historical micro rCBV | | | | | | | |

| | | PFS | | | OS | | |
|---|---|---|---|---|---|---|---|
| Co-variate | Timepoint | HR | 95% CI | P value | HR | 95% CI | P value |
| Age | Week 2 CRT | 1.021 | 0.952-1.095 | 0.555 | 1.022 | 0.941-1.110 | 0.607 |
| Current micro rCBF | | 0.994 | 0.975-1.013 | 0.531 | 0.997 | 0.977-1.018 | 0.781 |
| Historical micro rCBF | | 1.003 | 0.959-1.050 | 0.882 | 1.006 | 0.958-1.056 | 0.807 |
| Age | Week 3 CRT | 1.051 | 0.969-1.139 | 0.230 | 1.052 | 0.952-1.161 | 0.321 |
| Current micro rCBF | | 0.969 | 0.935-1.003 | 0.075 | 0.975 | 0.940-1.011 | 0.175 |
| Historical micro rCBF | | 1.016 | 0.991-1.043 | 0.215 | 1.020 | 0.991-1.049 | 0.176 |
| Age | Week 4 CRT | 1.044 | 0.954-1.142 | 0.354 | 1.075 | 0.949-1.217 | 0.254 |
| Current micro rCBF | | 0.974 | 0.932-1.017 | 0.229 | 0.959 | 0.916-1.003 | 0.068 |
| Historical micro rCBF | | 1.011 | 0.988-1.035 | 0.352 | 1.020 | 0.997-1.045 | 0.092 |
| Age | Week 5 CRT | 1.031 | 0.957-1.112 | 0.418 | 1.032 | 0.954-1.117 | 0.433 |
| Current micro rCBF | | 0.995 | 0.970-1.021 | 0.698 | 0.984 | 0.954-1.015 | 0.309 |
| Historical micro rCBF | | 0.995 | 0.955-1.037 | 0.804 | 1.010 | 0.972-1.050 | 0.610 |
| Age | Week 6 CRT | 1.039 | 0.966-1.117 | 0.305 | 1.018 | 0.950-1.091 | 0.613 |
| Current micro rCBF | | 0.985 | 0.950-1.020 | 0.394 | 0.977 | 0.939-1.016 | 0.240 |
| Historical micro rCBF | | 1.005 | 0.979-1.032 | 0.704 | 1.015 | 0.986-1.044 | 0.315 |
| Age | Pre-C1 | 1.046 | 0.976-1.121 | 0.201 | 1.037 | 0.964-1.114 | 0.329 |
| Current micro rCBF | | 0.967 | 0.923-1.012 | 0.147 | 0.962 | 0.907-1.021 | 0.208 |
| Historical micro rCBF | | 1.009 | 0.986-1.032 | 0.444 | 1.017 | 0.987-1.048 | 0.279 |
| Age | Pre-C2 | 0.933 | 0.816-1.066 | 0.309 | 1.030 | 0.918-1.157 | 0.613 |
| Current micro rCBF | | 1.068 | 0.981-1.164 | 0.130 | 1.188 | 0.987-1.429 | 0.069 |
| Historical micro rCBF | | 0.953 | 0.901-1.008 | 0.095 | 0.893 | 0.789-1.011 | 0.073 |
| Age | Pre-C3 | 1.031 | 0.947-1.122 | 0.484 | 1.003 | 0.922-1.090 | 0.951 |
| Current micro rCBF | | 0.987 | 0.932-1.045 | 0.649 | 1.004 | 0.937-1.076 | 0.912 |
| Historical micro rCBF | | 1.001 | 0.966-1.038 | 0.947 | 0.997 | 0.952-1.046 | 0.916 |
| Age | Pre-C4 | 1.102 | 0.903-1.345 | 0.340 | Inf | 0-Inf | N/A |
| Current micro rCBF | | 0.977 | 0.921-1.037 | 0.444 | Inf | 0-Inf | N/A |
| Historical micro rCBF | | 1.009 | 0.975-1.043 | 0.619 | Inf | 0-Inf | N/A |
| Age | Pre-C5 | Inf | 0-Inf | N/A | 1.003 | 0.911-1.105 | 0.945 |
| Current micro rCBF | | Inf | 0-Inf | N/A | 0.982 | 0.924-1.044 | 0.554 |
| Historical micro rCBF | | Inf | 0-Inf | N/A | 1.012 | 0.973-1.052 | 0.561 |
| Age | Pre-C6 | Inf | 0-Inf | N/A | Inf | 0-Inf | N/A |
| Current micro rCBF | | Inf | 0-Inf | N/A | Inf | 0-Inf | N/A |
| Historical micro rCBF | | | | | | | |
| Age | Post-C6 | Inf | 0-Inf | N/A | Inf | 0-Inf | N/A |
| Current micro rCBF | | | | | | | |
| Historical micro rCBF | | | | | | | |

| | | PFS | | | OS | | |
|---|---|---|---|---|---|---|---|
| Co-variate | Timepoint | HR | 95% CI | P value | HR | 95% CI | P value |
| Age | Week 2 CRT | 1.017 | 0.949-1.090 | 0.638 | 1.004 | 0.925-1.090 | 0.925 |
| Current pan rCBV | | 0.999 | 0.978-1.021 | 0.950 | 1.002 | 0.976-1.028 | 0.904 |
| Historical pan rCBV | | 0.996 | 0.957-1.037 | 0.839 | 1.000 | 0.953-1.049 | 0.990 |
| Age | Week 3 CRT | 1.056 | 0.969-1.152 | 0.216 | 1.053 | 0.958-1.158 | 0.285 |
| Current pan rCBV | | 0.988 | 0.963-1.013 | 0.346 | 0.977 | 0.950-1.005 | 0.108 |
| Historical pan rCBV | | 1.008 | 0.985-1.032 | 0.485 | 1.023 | 0.997-1.049 | 0.084 |
| Age | Week 4 CRT | 1.027 | 0.932-1.131 | 0.596 | 1.078 | 0.973-1.194 | 0.150 |
| Current pan rCBV | | 1.013 | 0.968-1.059 | 0.588 | 0.978 | 0.934-1.023 | 0.328 |
| Historical pan rCBV | | 0.987 | 0.952-1.023 | 0.482 | 1.017 | 0.980-1.055 | 0.375 |
| Age | Week 5 CRT | 1.053 | 0.976-1.136 | 0.179 | 1.070 | 0.993-1.153 | 0.077 |
| Current pan rCBV | | 0.987 | 0.941-1.035 | 0.585 | 0.954 | 0.907-1.003 | 0.065 |
| Historical pan rCBV | | 1.000 | 0.975-1.027 | 0.983 | 1.020 | 0.993-1.049 | 0.146 |
| Age | Week 6 CRT | 1.036 | 0.962-1.115 | 0.351 | 1.015 | 0.947-1.088 | 0.670 |
| Current pan rCBV | | 0.999 | 0.979-1.019 | 0.893 | 0.995 | 0.975-1.016 | 0.639 |
| Historical pan rCBV | | 0.997 | 0.978-1.016 | 0.740 | 1.003 | 0.984-1.023 | 0.738 |
| Age | Pre-C1 | 1.054 | 0.980-1.134 | 0.155 | 1.054 | 0.977-1.137 | 0.175 |
| Current pan rCBV | | 0.992 | 0.973-1.012 | 0.428 | 0.988 | 0.967-1.009 | 0.249 |
| Historical pan rCBV | | 1.001 | 0.988-1.014 | 0.869 | 1.007 | 0.993-1.021 | 0.317 |
| Age | Pre-C2 | 0.972 | 0.874-1.080 | 0.596 | 1.043 | 0.934-1.165 | 0.455 |
| Current pan rCBV | | 1.053 | 0.991-1.119 | 0.094 | 0.995 | 0.957-1.033 | 0.778 |
| Historical pan rCBV | | 0.956 | 0.911-1.003 | 0.064 | 1.003 | 0.978-1.030 | 0.800 |
| Age | Pre-C3 | 1.014 | 0.934-1.100 | 0.744 | 1.005 | 0.934-1.081 | 0.897 |
| Current pan rCBV | | 1.016 | 0.967-1.067 | 0.529 | 0.978 | 0.926-1.033 | 0.429 |
| Historical pan rCBV | | 0.983 | 0.953-1.015 | 0.293 | 1.014 | 0.978-1.051 | 0.451 |
| Age | Pre-C4 | 1.071 | 0.882-1.299 | 0.490 | | | |
| Current pan rCBV | | 0.998 | 0.933-1.068 | 0.957 | | | |
| Historical pan rCBV | | 0.997 | 0.963-1.032 | 0.855 | | | |
| Age | Pre-C5 | 1.082 | 0.885-1.322 | 0.444 | Inf | 0-Inf | N/A |
| Current pan rCBV | | 0.993 | 0.883-1.116 | 0.903 | Inf | 0-Inf | N/A |
| Historical pan rCBV | | 0.995 | 0.937-1.057 | 0.870 | Inf | 0-Inf | N/A |
| Age | Pre-C6 | Inf | 0-Inf | N/A | Inf | 0-Inf | N/A |
| Current pan rCBV | | Inf | 0-Inf | N/A | Inf | 0-Inf | N/A |
| Historical pan rCBV | | Inf | 0-Inf | N/A | Inf | 0-Inf | N/A |
| Age | Post-C6 | Inf | 0-Inf | N/A | Inf | 0-Inf | N/A |
| Current pan rCBV | | | | | | | |
| Historical pan rCBV | | | | | | | |

| | | PFS | | | OS | | |
|---|---|---|---|---|---|---|---|
| Co-variate | Timepoint | HR | 95% CI | P value | HR | 95% CI | P value |
| Age | Week 2 CRT | 1.017 | 0.949-1.090 | 0.638 | 1.003 | 0.924-1.089 | 0.939 |
| Current pan rCBF | | 1.000 | 0.982-1.018 | 0.962 | 1.003 | 0.981-1.027 | 0.778 |
| Historical pan rCBF | | 0.993 | 0.957-1.031 | 0.714 | 0.996 | 0.951-1.044 | 0.881 |
| Age | Week 3 CRT | 1.059 | 0.969-1.156 | 0.205 | 1.054 | 0.958-1.160 | 0.279 |
| Current pan rCBF | | 0.987 | 0.962-1.014 | 0.344 | 0.979 | 0.951-1.007 | 0.140 |
| Historical pan rCBF | | 1.008 | 0.985-1.031 | 0.512 | 1.021 | 0.996-1.047 | 0.099 |
| Age | Week 4 CRT | 1.007 | 0.908-1.116 | 0.900 | 1.042 | 0.932-1.165 | 0.468 |
| Current pan rCBF | | 1.018 | 0.987-1.049 | 0.258 | 1.004 | 0.976-1.034 | 0.766 |
| Historical pan rCBF | | 0.984 | 0.960-1.007 | 0.177 | 0.996 | 0.974-1.018 | 0.730 |
| Age | Week 5 CRT | 1.038 | 0.956-1.127 | 0.379 | 1.065 | 0.980-1.157 | 0.138 |
| Current pan rCBF | | 1.004 | 0.959-1.051 | 0.866 | 0.971 | 0.922-1.023 | 0.267 |
| Historical pan rCBF | | 0.999 | 0.973-1.025 | 0.917 | 1.019 | 0.990-1.048 | 0.207 |
| Age | Week 6 CRT | 1.038 | 0.964-1.117 | 0.327 | 1.016 | 0.948-1.088 | 0.651 |
| Current pan rCBF | | 0.996 | 0.978-1.015 | 0.699 | 0.994 | 0.975-1.014 | 0.556 |
| Historical pan rCBF | | 0.998 | 0.980-1.017 | 0.853 | 1.004 | 0.985-1.023 | 0.668 |
| Age | Pre-C1 | 1.055 | 0.978-1.138 | 0.169 | 1.053 | 0.975-1.139 | 0.189 |
| Current pan rCBF | | 0.992 | 0.973-1.012 | 0.437 | 0.989 | 0.969-1.009 | 0.292 |
| Historical pan rCBF | | 1.002 | 0.989-1.014 | 0.772 | 1.008 | 0.994-1.021 | 0.276 |
| Age | Pre-C2 | 0.966 | 0.866-1.078 | 0.536 | 1.044 | 0.935-1.166 | 0.443 |
| Current pan rCBF | | 1.051 | 0.991-1.115 | 0.099 | 0.994 | 0.959-1.031 | 0.762 |
| Historical pan rCBF | | 0.952 | 0.903-1.003 | 0.066 | 1.004 | 0.978-1.030 | 0.791 |
| Age | Pre-C3 | 1.018 | 0.933-1.110 | 0.691 | 1.005 | 0.933-1.083 | 0.886 |
| Current pan rCBF | | 1.015 | 0.961-1.072 | 0.592 | 0.980 | 0.925-1.037 | 0.479 |
| Historical pan rCBF | | 0.982 | 0.946-1.018 | 0.322 | 1.014 | 0.974-1.057 | 0.495 |
| Age | Pre-C4 | 1.063 | 0.873-1.294 | 0.545 | Inf | 0-Inf | N/A |
| Current pan rCBF | | 1.002 | 0.935-1.074 | 0.954 | Inf | 0-Inf | N/A |
| Historical pan rCBF | | 0.994 | 0.957-1.032 | 0.741 | Inf | 0-Inf | N/A |
| Age | Pre-C5 | 1.040 | 0.851-1.272 | 0.701 | Inf | 0-Inf | N/A |
| Current pan rCBF | | 1.028 | 0.913-1.159 | 0.646 | Inf | 0-Inf | N/A |
| Historical pan rCBF | | 0.973 | 0.904-1.046 | 0.453 | Inf | 0-Inf | N/A |
| Age | Pre-C6 | Inf | 0-Inf | N/A | Inf | 0-Inf | N/A |
| Current pan rCBF | | Inf | 0-Inf | N/A | Inf | 0-Inf | N/A |
| Historical pan rCBF | | | | | | | |
| Age | Post-C6 | Inf | 0-Inf | N/A | Inf | 0-Inf | N/A |
| Current pan rCBF | | | | | | | |
| Historical pan rCBF | | | | | | | |

**Supplementary Table 3**. Cox regression analysis using age, current percent change in imaging marker, and historical percent change in imaging marker **within the tumor (contrast-enhancing) ROI** as co-variates and their correlation with progression-free (PFS) and overall survival (OS) at each time point during and after treatment. Absence of values and "Inf" (infinity) reflect a lack of convergence of the "coxph" function, which can occur when there are a small number of observations with similar values for a given co-variate. In these cases, p-values cannot be reported ("N/A"). "Micro" = panvascular; derived from spin-echo sequences. "Pan" = panvascular; derived from gradient-echo sequences.

| | | PFS | | | OS | | |
|---|---|---|---|---|---|---|---|
| Co-variate | Timepoint | HR | 95% CI | P value | HR | 95% CI | P value |
| Age | Week 2 CRT | 1.012 | 0.944-1.086 | 0.729 | 1.008 | 0.922-1.101 | 0.863 |
| Current ADC | | 1.006 | 0.944-1.071 | 0.855 | 0.993 | 0.930-1.060 | 0.834 |
| Historical ADC | | 0.986 | 0.926-1.050 | 0.664 | 0.991 | 0.935-1.050 | 0.760 |
| Age | Week 3 CRT | 1.050 | 0.949-1.162 | 0.346 | 1.056 | 0.940-1.185 | 0.358 |
| Current ADC | | 1.003 | 0.972-1.035 | 0.843 | 0.994 | 0.956-1.032 | 0.745 |
| Historical ADC | | 0.992 | 0.912-1.079 | 0.849 | 1.011 | 0.920-1.111 | 0.820 |
| Age | Week 4 CRT | 1.036 | 0.947-1.133 | 0.443 | 1.040 | 0.936-1.155 | 0.465 |
| Current ADC | | 1.013 | 0.957-1.072 | 0.657 | 0.980 | 0.938-1.024 | 0.376 |
| Historical ADC | | 0.993 | 0.962-1.025 | 0.668 | 1.007 | 0.978-1.038 | 0.625 |
| Age | Week 5 CRT | 1.063 | 0.967-1.168 | 0.208 | 1.037 | 0.947-1.135 | 0.433 |
| Current ADC | | 1.020 | 0.965-1.078 | 0.491 | 0.984 | 0.943-1.027 | 0.469 |
| Historical ADC | | 0.996 | 0.971-1.023 | 0.789 | 1.005 | 0.980-1.031 | 0.696 |
| Age | Week 6 CRT | 1.017 | 0.937-1.105 | 0.679 | 1.016 | 0.929-1.110 | 0.731 |
| Current ADC | | 0.998 | 0.949-1.049 | 0.927 | 1.019 | 0.969-1.072 | 0.466 |
| Historical ADC | | 0.997 | 0.972-1.023 | 0.847 | 0.986 | 0.960-1.014 | 0.323 |
| Age | Pre-C1 | 1.051 | 0.958-1.152 | 0.294 | 1.073 | 0.958-1.201 | 0.224 |
| Current ADC | | 1.010 | 0.980-1.040 | 0.521 | 1.007 | 0.975-1.039 | 0.688 |
| Historical ADC | | 0.995 | 0.967-1.023 | 0.701 | 0.999 | 0.970-1.030 | 0.970 |
| Age | Pre-C2 | 0.963 | 0.863-1.075 | 0.505 | 1.037 | 0.902-1.191 | 0.611 |
| Current ADC | | 0.965 | 0.901-1.032 | 0.296 | 0.996 | 0.927-1.070 | 0.913 |
| Historical ADC | | 1.020 | 0.976-1.067 | 0.383 | 1.001 | 0.953-1.051 | 0.974 |
| Age | Pre-C3 | 1.000 | 0.923-1.084 | 0.997 | 0.949 | 0.855-1.052 | 0.320 |
| Current ADC | | 1.008 | 0.957-1.062 | 0.759 | 0.956 | 0.896-1.019 | 0.167 |
| Historical ADC | | 0.987 | 0.938-1.040 | 0.630 | 1.023 | 0.966-1.084 | 0.438 |
| Age | Pre-C4 | 1.090 | 0.364-3.268 | 0.878 | Inf | 0-Inf | N/A |
| Current ADC | | 1.018 | 0.809-1.279 | 0.882 | Inf | 0-Inf | N/A |
| Historical ADC | | 0.878 | 0.650-1.185 | 0.394 | Inf | 0-Inf | N/A |
| Age | Pre-C5 | 0.896 | 0.652-1.229 | 0.495 | Inf | 0-Inf | N/A |
| Current ADC | | 1.728 | 0.454-6.572 | 0.422 | Inf | 0-Inf | N/A |
| Historical ADC | | Inf | 0-Inf | 0.416 | Inf | 0-Inf | N/A |
| Age | Pre-C6 | Inf | 0-Inf | N/A | Inf | 0-Inf | N/A |
| Current ADC | | Inf | 0-Inf | N/A | Inf | 0-Inf | N/A |
| Historical ADC | | | | | | | |
| Age | Post-C6 | Inf | 0-Inf | N/A | Inf | 0-Inf | N/A |
| Current ADC | | | | | | | |
| Historical ADC | | | | | | | |

| | | PFS | | | OS | | |
|---|---|---|---|---|---|---|---|
| Co-variate | Timepoint | HR | 95% CI | P value | HR | 95% CI | P value |
| Age | Week 2 CRT | 0.946 | 0.866-1.034 | 0.224 | 0.992 | 0.923-1.066 | 0.820 |
| Current $K^{trans}$ | | 1.101 | 1.014-1.195 | 0.021 | 1.034 | 0.987-1.084 | 0.158 |
| Historical $K^{trans}$ | | 0.899 | 0.821-0.986 | 0.024 | 0.973 | 0.922-1.027 | 0.317 |
| Age | Week 3 CRT | 1.044 | 0.952-1.144 | 0.365 | 1.046 | 0.938-1.166 | 0.421 |
| Current $K^{trans}$ | | 1.003 | 0.984-1.023 | 0.754 | 1.001 | 0.980-1.023 | 0.930 |
| Historical $K^{trans}$ | | 1.000 | 0.973-1.027 | 0.996 | 1.006 | 0.975-1.038 | 0.731 |
| Age | Week 4 CRT | 1.026 | 0.958-1.099 | 0.466 | 1.059 | 0.970-1.157 | 0.200 |
| Current $K^{trans}$ | | 0.990 | 0.970-1.010 | 0.319 | 0.975 | 0.951-1.000 | 0.049 |
| Historical $K^{trans}$ | | 1.018 | 0.986-1.051 | 0.264 | 1.040 | 0.999-1.083 | 0.059 |
| Age | Week 5 CRT | 1.060 | 0.976-1.150 | 0.165 | 1.047 | 0.965-1.136 | 0.272 |
| Current $K^{trans}$ | | 1.038 | 1.013-1.064 | 0.003 | 1.006 | 0.994-1.018 | 0.316 |
| Historical $K^{trans}$ | | 0.945 | 0.909-0.983 | 0.005 | 0.988 | 0.965-1.011 | 0.314 |
| Age | Week 6 CRT | 1.014 | 0.943-1.090 | 0.712 | 0.997 | 0.927-1.072 | 0.933 |
| Current $K^{trans}$ | | 1.008 | 0.986-1.030 | 0.488 | 1.011 | 0.988-1.034 | 0.361 |
| Historical $K^{trans}$ | | 0.993 | 0.969-1.018 | 0.568 | 0.991 | 0.966-1.017 | 0.502 |
| Age | Pre-C1 | 1.144 | 1.037-1.263 | 0.008 | 1.079 | 1.002-1.163 | 0.045 |
| Current $K^{trans}$ | | 1.029 | 1.009-1.050 | 0.004 | 1.015 | 1.002-1.028 | 0.027 |
| Historical $K^{trans}$ | | 0.991 | 0.981-1.000 | 0.054 | 0.992 | 0.981-1.003 | 0.146 |
| Age | Pre-C2 | 0.995 | 0.903-1.096 | 0.921 | 1.042 | 0.928-1.170 | 0.486 |
| Current $K^{trans}$ | | 0.990 | 0.974-1.006 | 0.203 | 1.001 | 0.988-1.015 | 0.837 |
| Historical $K^{trans}$ | | 1.009 | 0.996-1.021 | 0.178 | 0.999 | 0.990-1.008 | 0.836 |
| Age | Pre-C3 | 1.053 | 0.956-1.160 | 0.291 | 1.016 | 0.933-1.107 | 0.711 |
| Current $K^{trans}$ | | 0.976 | 0.941-1.011 | 0.179 | 0.993 | 0.982-1.003 | 0.183 |
| Historical $K^{trans}$ | | 1.027 | 0.984-1.072 | 0.226 | 1.013 | 0.998-1.029 | 0.085 |
| Age | Pre-C4 | 1.177 | 0.795-1.742 | 0.416 | Inf | 0-Inf | N/A |
| Current $K^{trans}$ | | 0.999 | 0.945-1.055 | 0.962 | Inf | 0-Inf | N/A |
| Historical $K^{trans}$ | | 1.012 | 0.957-1.070 | 0.678 | Inf | 0-Inf | N/A |
| Age | Pre-C5 | Inf | 0-Inf | N/A | 0.944 | 0.814-1.094 | 0.444 |
| Current $K^{trans}$ | | Inf | 0-Inf | N/A | 1.014 | 0.988-1.041 | 0.296 |
| Historical $K^{trans}$ | | Inf | 0-Inf | N/A | 0.975 | 0.930-1.022 | 0.298 |
| Age | Pre-C6 | Inf | 0-Inf | N/A | Inf | 0-Inf | N/A |
| Current $K^{trans}$ | | Inf | 0-Inf | N/A | Inf | 0-Inf | N/A |
| Historical $K^{trans}$ | | | | | | | |
| Age | Post-C6 | Inf | 0-Inf | N/A | Inf | 0-Inf | N/A |
| Current $K^{trans}$ | | | | | | | |
| Historical $K^{trans}$ | | | | | | | |

| | | PFS | | | OS | | |
|---|---|---|---|---|---|---|---|
| Co-variate | Timepoint | HR | 95% CI | P value | HR | 95% CI | P value |
| Age | Week 2 CRT | 1.021 | 0.958-1.088 | 0.519 | 1.014 | 0.943-1.091 | 0.700 |
| Current micro rCBV | | 1.035 | 0.966-1.108 | 0.328 | 1.007 | 0.944-1.075 | 0.829 |
| Historical micro rCBV | | 0.956 | 0.877-1.041 | 0.301 | 1.000 | 0.926-1.080 | 1.000 |
| Age | Week 3 CRT | 1.051 | 0.954-1.157 | 0.316 | 1.050 | 0.937-1.177 | 0.397 |
| Current micro rCBV | | 0.998 | 0.965-1.032 | 0.903 | 0.983 | 0.957-1.009 | 0.204 |
| Historical micro rCBV | | 1.002 | 0.952-1.055 | 0.936 | 1.030 | 0.990-1.072 | 0.148 |
| Age | Week 4 CRT | 0.994 | 0.920-1.074 | 0.882 | 0.996 | 0.906-1.094 | 0.927 |
| Current micro rCBV | | 1.018 | 0.975-1.062 | 0.426 | 0.995 | 0.955-1.038 | 0.826 |
| Historical micro rCBV | | 0.997 | 0.957-1.039 | 0.892 | 1.014 | 0.967-1.062 | 0.569 |
| Age | Week 5 CRT | 1.022 | 0.950-1.100 | 0.556 | 1.012 | 0.932-1.099 | 0.770 |
| Current micro rCBV | | 1.020 | 0.967-1.075 | 0.471 | 0.991 | 0.946-1.039 | 0.710 |
| Historical micro rCBV | | 0.989 | 0.945-1.035 | 0.627 | 1.014 | 0.969-1.062 | 0.550 |
| Age | Week 6 CRT | 1.019 | 0.950-1.092 | 0.603 | 1.009 | 0.943-1.080 | 0.800 |
| Current micro rCBV | | 1.000 | 0.966-1.035 | 0.999 | 1.003 | 0.968-1.039 | 0.870 |
| Historical micro rCBV | | 1.007 | 0.977-1.037 | 0.659 | 1.009 | 0.978-1.040 | 0.583 |
| Age | Pre-C1 | 1.010 | 0.944-1.082 | 0.763 | 1.039 | 0.957-1.128 | 0.367 |
| Current micro rCBV | | 0.998 | 0.972-1.024 | 0.881 | 0.982 | 0.958-1.006 | 0.135 |
| Historical micro rCBV | | 1.004 | 0.983-1.025 | 0.693 | 1.014 | 0.992-1.036 | 0.222 |
| Age | Pre-C2 | 0.846 | 0.656-1.091 | 0.198 | 0.953 | 0.848-1.072 | 0.423 |
| Current micro rCBV | | 1.123 | 0.972-1.298 | 0.115 | 1.089 | 0.979-1.211 | 0.118 |
| Historical micro rCBV | | 0.959 | 0.905-1.016 | 0.158 | 0.973 | 0.931-1.016 | 0.217 |
| Age | Pre-C3 | 0.994 | 0.909-1.087 | 0.896 | 1.018 | 0.922-1.123 | 0.725 |
| Current micro rCBV | | 1.006 | 0.936-1.081 | 0.873 | 0.978 | 0.885-1.081 | 0.667 |
| Historical micro rCBV | | 1.005 | 0.981-1.031 | 0.681 | 1.012 | 0.975-1.050 | 0.533 |
| Age | Pre-C4 | 1.461 | 0.788-2.708 | 0.229 | 1.148 | 0.786-1.676 | 0.476 |
| Current micro rCBV | | 1.041 | 0.916-1.183 | 0.533 | 0.981 | 0.862-1.117 | 0.775 |
| Historical micro rCBV | | 1.113 | 0.952-1.302 | 0.180 | 0.980 | 0.832-1.154 | 0.811 |
| Age | Pre-C5 | Inf | 0-Inf | N/A | 0.955 | 0.735-1.240 | 0.730 |
| Current micro rCBV | | Inf | 0-Inf | N/A | 1.015 | 0.970-1.063 | 0.517 |
| Historical micro rCBV | | Inf | 0-Inf | N/A | 0.970 | 0.792-1.187 | 0.766 |
| Age | Pre-C6 | Inf | 0-Inf | N/A | Inf | 0-Inf | N/A |
| Current micro rCBV | | Inf | 0-Inf | N/A | Inf | 0-Inf | N/A |
| Historical micro rCBV | | Inf | 0-Inf | N/A | Inf | 0-Inf | N/A |
| Age | Post-C6 | Inf | 0-Inf | N/A | Inf | 0-Inf | N/A |
| Current micro rCBV | | | | | | | |
| Historical micro rCBV | | | | | | | |

| | | PFS | | | OS | | |
|---|---|---|---|---|---|---|---|
| Co-variate | Timepoint | HR | 95% CI | P value | HR | 95% CI | P value |
| Age | Week 2 CRT | 1.001 | 0.934-1.073 | 0.980 | 1.006 | 0.927-1.093 | 0.881 |
| Current micro rCBF | | 0.985 | 0.933-1.041 | 0.601 | 0.984 | 0.949-1.020 | 0.379 |
| Historical micro rCBF | | 1.023 | 0.955-1.097 | 0.518 | 1.030 | 0.986-1.076 | 0.188 |
| Age | Week 3 CRT | 1.047 | 0.948-1.156 | 0.365 | 1.051 | 0.934-1.182 | 0.412 |
| Current micro rCBF | | 0.953 | 0.908-1.000 | 0.050 | 0.981 | 0.949-1.014 | 0.255 |
| Historical micro rCBF | | 1.078 | 1.001-1.161 | 0.046 | 1.039 | 0.987-1.094 | 0.146 |
| Age | Week 4 CRT | 0.988 | 0.918-1.064 | 0.746 | 0.981 | 0.896-1.074 | 0.676 |
| Current micro rCBF | | 1.015 | 0.968-1.063 | 0.541 | 0.993 | 0.950-1.039 | 0.775 |
| Historical micro rCBF | | 0.998 | 0.961-1.037 | 0.926 | 1.018 | 0.980-1.059 | 0.358 |
| Age | Week 5 CRT | 1.014 | 0.945-1.089 | 0.698 | 1.002 | 0.925-1.085 | 0.969 |
| Current micro rCBF | | 1.013 | 0.960-1.069 | 0.629 | 0.990 | 0.953-1.029 | 0.623 |
| Historical micro rCBF | | 0.995 | 0.955-1.036 | 0.796 | 1.016 | 0.984-1.050 | 0.333 |
| Age | Week 6 CRT | 1.018 | 0.947-1.094 | 0.630 | 1.008 | 0.934-1.087 | 0.840 |
| Current micro rCBF | | 0.994 | 0.950-1.040 | 0.804 | 0.990 | 0.953-1.029 | 0.620 |
| Historical micro rCBF | | 1.009 | 0.977-1.043 | 0.584 | 1.014 | 0.985-1.044 | 0.333 |
| Age | Pre-C1 | 1.023 | 0.949-1.104 | 0.550 | 1.053 | 0.951-1.165 | 0.322 |
| Current micro rCBF | | 0.979 | 0.943-1.017 | 0.281 | 0.965 | 0.926-1.005 | 0.086 |
| Historical micro rCBF | | 1.015 | 0.992-1.038 | 0.212 | 1.024 | 0.999-1.051 | 0.063 |
| Age | Pre-C2 | 0.889 | 0.727-1.087 | 0.251 | 0.779 | 0.559-1.086 | 0.141 |
| Current micro rCBF | | 1.074 | 0.930-1.241 | 0.330 | 1.442 | 0.855-2.432 | 0.170 |
| Historical micro rCBF | | 0.969 | 0.901-1.043 | 0.400 | 0.841 | 0.654-1.083 | 0.180 |
| Age | Pre-C3 | 1.038 | 0.943-1.143 | 0.448 | 0.984 | 0.876-1.105 | 0.788 |
| Current micro rCBF | | 0.944 | 0.843-1.058 | 0.322 | 1.012 | 0.894-1.145 | 0.851 |
| Historical micro rCBF | | 1.025 | 0.980-1.073 | 0.277 | 1.006 | 0.962-1.052 | 0.779 |
| Age | Pre-C4 | Inf | 0-Inf | N/A | Inf | 0-Inf | N/A |
| Current micro rCBF | | Inf | 0-Inf | N/A | Inf | 0-Inf | N/A |
| Historical micro rCBF | | Inf | 0-Inf | N/A | Inf | 0-Inf | N/A |
| Age | Pre-C5 | Inf | 0-Inf | N/A | 1.080 | 0.803-1.454 | 0.610 |
| Current micro rCBF | | Inf | 0-Inf | N/A | 1.049 | 0.957-1.150 | 0.308 |
| Historical micro rCBF | | Inf | 0-Inf | N/A | 1.130 | 0.805-1.586 | 0.479 |
| Age | Pre-C6 | Inf | 0-Inf | N/A | Inf | 0-Inf | N/A |
| Current micro rCBF | | Inf | 0-Inf | N/A | Inf | 0-Inf | N/A |
| Historical micro rCBF | | | | | | | |
| Age | Post-C6 | Inf | 0-Inf | N/A | Inf | 0-Inf | N/A |
| Current micro rCBF | | | | | | | |
| Historical micro rCBF | | | | | | | |

| | | PFS | | | OS | | |
|---|---|---|---|---|---|---|---|
| Co-variate | Timepoint | HR | 95% CI | P value | HR | 95% CI | P value |
| Age | Week 2 CRT | 1.015 | 0.951-1.083 | 0.661 | 0.994 | 0.924-1.070 | 0.874 |
| Current pan rCBV | | 1.007 | 0.952-1.065 | 0.799 | 0.945 | 0.886-1.007 | 0.082 |
| Historical pan rCBV | | 1.009 | 0.938-1.084 | 0.816 | 1.115 | 1.006-1.235 | 0.038 |
| Age | Week 3 CRT | 1.046 | 0.962-1.137 | 0.290 | 1.068 | 0.955-1.194 | 0.249 |
| Current pan rCBV | | 0.983 | 0.941-1.028 | 0.457 | 0.986 | 0.943-1.031 | 0.530 |
| Historical pan rCBV | | 1.034 | 0.963-1.109 | 0.361 | 1.026 | 0.967-1.088 | 0.403 |
| Age | Week 4 CRT | 1.027 | 0.960-1.099 | 0.441 | 1.028 | 0.952-1.109 | 0.484 |
| Current pan rCBV | | 1.007 | 0.960-1.056 | 0.778 | 0.978 | 0.938-1.020 | 0.297 |
| Historical pan rCBV | | 1.010 | 0.948-1.075 | 0.761 | 1.044 | 0.979-1.113 | 0.189 |
| Age | Week 5 CRT | 1.041 | 0.970-1.117 | 0.270 | 1.045 | 0.974-1.121 | 0.222 |
| Current pan rCBV | | 1.012 | 0.984-1.040 | 0.401 | 0.984 | 0.959-1.011 | 0.247 |
| Historical pan rCBV | | 0.995 | 0.954-1.037 | 0.808 | 1.031 | 0.987-1.078 | 0.173 |
| Age | Week 6 CRT | 1.025 | 0.961-1.093 | 0.461 | 1.016 | 0.953-1.084 | 0.624 |
| Current pan rCBV | | 1.003 | 0.967-1.040 | 0.874 | 0.998 | 0.965-1.032 | 0.914 |
| Historical pan rCBV | | 1.011 | 0.971-1.051 | 0.601 | 1.023 | 0.984-1.065 | 0.251 |
| Age | Pre-C1 | 1.049 | 0.983-1.120 | 0.149 | 1.087 | 0.997-1.186 | 0.059 |
| Current pan rCBV | | 0.976 | 0.934-1.020 | 0.276 | 0.956 | 0.917-0.997 | 0.037 |
| Historical pan rCBV | | 1.038 | 0.990-1.089 | 0.127 | 1.048 | 0.999-1.098 | 0.055 |
| Age | Pre-C2 | 0.990 | 0.899-1.090 | 0.843 | 1.016 | 0.921-1.120 | 0.756 |
| Current pan rCBV | | 1.003 | 0.959-1.049 | 0.882 | 1.032 | 0.978-1.089 | 0.252 |
| Historical pan rCBV | | 1.012 | 0.981-1.049 | 0.454 | 0.982 | 0.952-1.014 | 0.269 |
| Age | Pre-C3 | 1.000 | 0.935-1.071 | 0.992 | 0.987 | 0.907-1.074 | 0.763 |
| Current pan rCBV | | 1.007 | 0.956-1.060 | 0.796 | 1.025 | 0.975-1.078 | 0.334 |
| Historical pan rCBV | | 1.022 | 0.990-1.055 | 0.172 | 0.989 | 0.964-1.015 | 0.396 |
| Age | Pre-C4 | Inf | 0-Inf | N/A | 1.232 | 0.905-1.679 | 0.185 |
| Current pan rCBV | | Inf | 0-Inf | N/A | 0.989 | 0.909-1.077 | 0.800 |
| Historical pan rCBV | | Inf | 0-Inf | N/A | 0.977 | 0.911-1.048 | 0.520 |
| Age | Pre-C5 | 1.172 | 0.905-1.517 | 0.229 | Inf | 0-Inf | N/A |
| Current pan rCBV | | 0.979 | 0.913-1.049 | 0.539 | Inf | 0-Inf | N/A |
| Historical pan rCBV | | 1.152 | 0.913-1.455 | 0.233 | Inf | 0-Inf | N/A |
| Age | Pre-C6 | Inf | 0-Inf | N/A | Inf | 0-Inf | N/A |
| Current pan rCBV | | Inf | 0-Inf | N/A | Inf | 0-Inf | N/A |
| Historical pan rCBV | | Inf | 0-Inf | N/A | Inf | 0-Inf | N/A |
| Age | Post-C6 | Inf | 0-Inf | N/A | Inf | 0-Inf | N/A |
| Current pan rCBV | | | | | | | |
| Historical pan rCBV | | | | | | | |

| | | PFS | | | OS | | |
|---|---|---|---|---|---|---|---|
| Co-variate | Timepoint | HR | 95% CI | P value | HR | 95% CI | P value |
| Age | Week 2 CRT | 1.011 | 0.941-1.086 | 0.760 | 0.979 | 0.904-1.059 | 0.592 |
| Current pan rCBF | | 1.006 | 0.957-1.058 | 0.818 | 0.957 | 0.909-1.007 | 0.089 |
| Historical pan rCBF | | 1.004 | 0.947-1.065 | 0.884 | 1.076 | 1.003-1.156 | 0.042 |
| Age | Week 3 CRT | 1.051 | 0.967-1.143 | 0.240 | 1.067 | 0.952-1.196 | 0.264 |
| Current pan rCBF | | 0.981 | 0.939-1.024 | 0.378 | 0.991 | 0.949-1.035 | 0.681 |
| Historical pan rCBF | | 1.037 | 0.964-1.116 | 0.327 | 1.019 | 0.956-1.086 | 0.560 |
| Age | Week 4 CRT | 1.037 | 0.954-1.129 | 0.393 | 1.030 | 0.940-1.129 | 0.524 |
| Current pan rCBF | | 1.027 | 0.975-1.081 | 0.317 | 0.989 | 0.943-1.037 | 0.643 |
| Historical pan rCBF | | 0.981 | 0.925-1.040 | 0.517 | 1.020 | 0.963-1.080 | 0.507 |
| Age | Week 5 CRT | 1.042 | 0.965-1.125 | 0.293 | 1.039 | 0.966-1.119 | 0.302 |
| Current pan rCBF | | 1.016 | 0.990-1.042 | 0.239 | 0.99 | 0.967-1.014 | 0.413 |
| Historical pan rCBF | | 0.989 | 0.959-1.020 | 0.477 | 1.017 | 0.986-1.050 | 0.288 |
| Age | Week 6 CRT | 1.025 | 0.957-1.097 | 0.489 | 1.010 | 0.946-1.079 | 0.762 |
| Current pan rCBF | | 1.011 | 0.970-1.054 | 0.609 | 0.999 | 0.963-1.037 | 0.968 |
| Historical pan rCBF | | 0.998 | 0.962-1.037 | 0.934 | 1.013 | 0.978-1.049 | 0.480 |
| Age | Pre-C1 | 1.040 | 0.971-1.114 | 0.261 | 1.079 | 0.988-1.178 | 0.089 |
| Current pan rCBF | | 0.987 | 0.950-1.026 | 0.509 | 0.962 | 0.930-0.996 | 0.028 |
| Historical pan rCBF | | 1.018 | 0.985-1.052 | 0.292 | 1.037 | 1.002-1.072 | 0.035 |
| Age | Pre-C2 | 0.986 | 0.895-1.088 | 0.785 | 1.016 | 0.922-1.119 | 0.750 |
| Current pan rCBF | | 1.004 | 0.953-1.057 | 0.892 | 1.030 | 0.978-1.084 | 0.259 |
| Historical pan rCBF | | 1.005 | 0.971-1.040 | 0.778 | 0.983 | 0.954-1.014 | 0.285 |
| Age | Pre-C3 | 0.994 | 0.925-1.069 | 0.874 | 0.988 | 0.910-1.072 | 0.771 |
| Current pan rCBF | | 1.003 | 0.944-1.065 | 0.932 | 1.034 | 0.976-1.095 | 0.260 |
| Historical pan rCBF | | 1.012 | 0.980-1.046 | 0.457 | 0.988 | 0.962-1.015 | 0.395 |
| Age | Pre-C4 | Inf | 0-Inf | N/A | 1.264 | 0.898-1.779 | 0.179 |
| Current pan rCBF | | Inf | 0-Inf | N/A | 0.970 | 0.871-1.081 | 0.583 |
| Historical pan rCBF | | Inf | 0-Inf | N/A | 0.978 | 0.890-1.075 | 0.649 |
| Age | Pre-C5 | 1.351 | 0.709-2.576 | 0.361 | Inf | 0-Inf | N/A |
| Current pan rCBF | | 0.990 | 0.911-1.075 | 0.813 | Inf | 0-Inf | N/A |
| Historical pan rCBF | | 1.268 | 0.763-2.108 | 0.359 | Inf | 0-Inf | N/A |
| Age | Pre-C6 | Inf | 0-Inf | N/A | Inf | 0-Inf | N/A |
| Current pan rCBF | | Inf | 0-Inf | N/A | Inf | 0-Inf | N/A |
| Historical pan rCBF | | Inf | 0-Inf | N/A | Inf | 0-Inf | N/A |
| Age | Post-C6 | Inf | 0-Inf | N/A | Inf | 0-Inf | N/A |
| Current pan rCBF | | | | | | | |
| Historical pan rCBF | | | | | | | |

**Supplementary Table 4**. Cox regression analysis using age, current percent change in VAI-derived metrics “vortex area” (a composite parameter of vessel type and oxygen saturation) and “vessel size index”, and historical percent change in “vortex area” and “vessel size index” as co-variates and their correlation with progression-free (PFS) and overall survival (OS) at each time point during and after treatment. Lack of values and “Inf” (infinity) reflect a lack of convergence of the “coxph” function, which can occur when there are a small number of observations with similar values for a given co-variate. In these cases, p-values cannot be reported (“N/A”).

| | | PFS | | | OS | | |
|---|---|---|---|---|---|---|---|
| Co-variate | Timepoint | HR | 95% CI | P value | HR | 95% CI | P value |
| Age | Week 2 CRT | 1.106 | 0.995-1.231 | 0.063 | 1.085 | 0.979-1.202 | 0.120 |
| Current vortex area* | | 1.001 | 0.974-1.030 | 0.933 | 1.012 | 0.980-1.045 | 0.466 |
| Historical vortex area* | | 1.052 | 1.003-1.104 | 0.038 | 1.038 | 1.000-1.077 | 0.050 |
| Age | Week 3 CRT | 1.194 | 0.981-1.452 | 0.077 | 1.157 | 0.985-1.360 | 0.076 |
| Current vortex area | | 0.999 | 0.967-1.032 | 0.961 | 1.003 | 0.972-1.035 | 0.844 |
| Historical vortex area | | 0.998 | 0.970-1.026 | 0.861 | 1.018 | 0.988-1.049 | 0.239 |
| Age | Week 4 CRT | 1.038 | 0.964-1.119 | 0.325 | 1.017 | 0.940-1.100 | 0.682 |
| Current vortex area | | 1.018 | 0.998-1.038 | 0.073 | 1.002 | 0.988-1.017 | 0.753 |
| Historical vortex area | | 1.005 | 0.974-1.037 | 0.769 | 1.028 | 0.993-1.064 | 0.114 |
| Age | Week 5 CRT | 1.057 | 0.981-1.138 | 0.145 | 1.046 | 0.959-1.142 | 0.312 |
| Current vortex area | | 0.996 | 0.974-1.017 | 0.690 | 0.986 | 0.966-1.006 | 0.176 |
| Historical vortex area | | 1.030 | 0.995-1.066 | 0.099 | 1.030 | 0.996-1.066 | 0.086 |
| Age | Week 6 CRT | 1.041 | 0.966-1.122 | 0.289 | 1.062 | 0.970-1.162 | 0.193 |
| Current vortex area | | 0.994 | 0.954-1.037 | 0.789 | 0.985 | 0.949-1.022 | 0.420 |
| Historical vortex area | | 1.022 | 0.962-1.087 | 0.477 | 1.041 | 0.982-1.103 | 0.175 |
| Age | Pre-C1 | 1.042 | 0.968-1.121 | 0.274 | 1.090 | 0.972-1.222 | 0.140 |
| Current vortex area | | 0.997 | 0.971-1.023 | 0.798 | 0.978 | 0.954-1.003 | 0.087 |
| Historical vortex area | | 1.021 | 0.975-1.069 | 0.373 | 1.035 | 0.989-1.083 | 0.143 |
| Age | Pre-C2 | Inf | 0-Inf | N/A | 0.995 | 0.856-1.157 | 0.953 |
| Current vortex area | | Inf | 0-Inf | N/A | 1.031 | 0.981-1.083 | 0.232 |
| Historical vortex area | | Inf | 0-Inf | N/A | 0.981 | 0.947-1.016 | 0.283 |

* Refers to tumor-to-reference tissue ratio. Reference tissue is defined as contralateral normal brain.

| | | PFS | | | OS | | |
|---|---|---|---|---|---|---|---|
| Co-variate | Timepoint | HR | 95% CI | P value | HR | 95% CI | P value |
| Age | Week 2 CRT | 1.106 | 0.995-1.231 | 0.063 | 1.085 | 0.979-1.202 | 0.120 |
| Current vortex area* | | 1.001 | 0.974-1.030 | 0.933 | 1.012 | 0.980-1.045 | 0.466 |
| Historical vortex area* | | 1.052 | 1.003-1.104 | 0.038 | 1.038 | 1.000-1.077 | 0.050 |
| Age | Week 3 CRT | 1.194 | 0.981-1.452 | 0.077 | 1.157 | 0.985-1.360 | 0.076 |
| Current vortex area | | 0.999 | 0.967-1.032 | 0.961 | 1.003 | 0.972-1.035 | 0.844 |
| Historical vortex area | | 0.998 | 0.970-1.026 | 0.861 | 1.018 | 0.988-1.049 | 0.239 |
| Age | Week 4 CRT | 1.038 | 0.964-1.119 | 0.325 | 1.017 | 0.940-1.100 | 0.682 |
| Current vortex area | | 1.018 | 0.998-1.038 | 0.073 | 1.002 | 0.988-1.017 | 0.753 |
| Historical vortex area | | 1.005 | 0.974-1.037 | 0.769 | 1.028 | 0.993-1.064 | 0.114 |
| Age | Week 5 CRT | 1.057 | 0.981-1.138 | 0.145 | 1.046 | 0.959-1.142 | 0.312 |
| Current vortex area | | 0.996 | 0.974-1.017 | 0.690 | 0.986 | 0.966-1.006 | 0.176 |
| Historical vortex area | | 1.030 | 0.995-1.066 | 0.099 | 1.030 | 0.996-1.066 | 0.086 |
| Age | Week 6 CRT | 1.041 | 0.966-1.122 | 0.289 | 1.062 | 0.970-1.162 | 0.193 |
| Current vortex area | | 0.994 | 0.954-1.037 | 0.789 | 0.985 | 0.949-1.022 | 0.420 |
| Historical vortex area | | 1.022 | 0.962-1.087 | 0.477 | 1.041 | 0.982-1.103 | 0.175 |
| Age | Pre-C1 | 1.042 | 0.968-1.121 | 0.274 | 1.090 | 0.972-1.222 | 0.140 |
| Current vortex area | | 0.997 | 0.971-1.023 | 0.798 | 0.978 | 0.954-1.003 | 0.087 |
| Historical vortex area | | 1.021 | 0.975-1.069 | 0.373 | 1.035 | 0.989-1.083 | 0.143 |
| Age | Pre-C2 | Inf | 0-Inf | N/A | 0.995 | 0.856-1.157 | 0.953 |
| Current vortex area | | Inf | 0-Inf | N/A | 1.031 | 0.981-1.083 | 0.232 |
| Historical vortex area | | Inf | 0-Inf | N/A | 0.981 | 0.947-1.016 | 0.283 |

* Refers to *normalized* tumor-to-reference tissue ratio.

| | | PFS | | | OS | | |
|---|---|---|---|---|---|---|---|
| Co-variate | Timepoint | HR | 95% CI | P value | HR | 95% CI | P value |
| Age | Week 2 CRT | 1.060 | 0.960-1.171 | 0.247 | 1.095 | 0.983-1.221 | 0.100 |
| Current vortex area (venous)* | | 0.994 | 0.964-1.026 | 0.723 | 1.014 | 0.982-1.046 | 0.393 |
| Historical vortex area (venous)* | | 1.035 | 0.984-1.089 | 0.182 | 1.047 | 0.993-1.105 | 0.092 |
| Age | Week 3 CRT | 1.185 | 0.981-1.432 | 0.078 | 1.175 | 1.004-1.375 | 0.044 |
| Current vortex area (venous) | | 1.009 | 0.955-1.067 | 0.740 | 1.030 | 0.978-1.084 | 0.263 |
| Historical vortex area (venous) | | 0.998 | 0.958-1.040 | 0.932 | 1.028 | 0.984-1.075 | 0.218 |
| Age | Week 4 CRT | 1.019 | 0.956-1.085 | 0.569 | 1.031 | 0.962-1.104 | 0.394 |
| Current vortex area (venous) | | 1.030 | 0.995-1.066 | 0.098 | 1.011 | 0.981-1.041 | 0.488 |
| Historical vortex area (venous) | | 0.995 | 0.961-1.029 | 0.760 | 1.029 | 0.994-1.066 | 0.104 |
| Age | Week 5 CRT | 1.053 | 0.981-1.131 | 0.154 | 1.070 | 0.989-1.159 | 0.093 |
| Current vortex area (venous) | | 1.005 | 0.983-1.027 | 0.680 | 0.987 | 0.963-1.011 | 0.276 |
| Historical vortex area (venous) | | 1.036 | 0.988-1.088 | 0.146 | 1.062 | 1.001-1.127 | 0.047 |
| Age | Week 6 CRT | 1.031 | 0.957-1.110 | 0.425 | 1.049 | 0.969-1.137 | 0.238 |
| Current vortex area (venous) | | 1.006 | 0.977-1.036 | 0.689 | 0.995 | 0.967-1.024 | 0.755 |
| Historical vortex area (venous) | | 1.011 | 0.962-1.064 | 0.662 | 1.040 | 0.987-1.096 | 0.142 |
| Age | Pre-C1 | 1.047 | 0.969-1.133 | 0.246 | 1.107 | 0.991-1.238 | 0.073 |
| Current vortex area (venous) | | 1.001 | 0.978-1.024 | 0.935 | 0.976 | 0.950-1.002 | 0.071 |
| Historical vortex area (venous) | | 1.020 | 0.977-1.065 | 0.362 | 1.050 | 0.993-1.109 | 0.086 |
| Age | Pre-C2 | Inf | 0-Inf | N/A | 0.994 | 0.864-1.144 | 0.936 |
| Current vortex area (venous) | | Inf | 0-Inf | N/A | 1.049 | 0.986-1.116 | 0.134 |
| Historical vortex area (venous) | | Inf | 0-Inf | N/A | 0.959 | 0.905-1.016 | 0.157 |

* Refers to tumor-to-reference tissue ratio. Reference tissue is defined as contralateral normal brain.

| | | PFS | | | OS | | |
|---|---|---|---|---|---|---|---|
| Co-variate | Timepoint | HR | 95% CI | P value | HR | 95% CI | P value |
| Age | Week 2 CRT | 1.060 | 0.960-1.171 | 0.247 | 1.095 | 0.983-1.221 | 0.100 |
| Current vortex area (venous)* | | 0.994 | 0.964-1.026 | 0.723 | 1.014 | 0.982-1.046 | 0.393 |
| Historical vortex area (venous)* | | 1.035 | 0.984-1.089 | 0.182 | 1.047 | 0.993-1.105 | 0.092 |
| Age | Week 3 CRT | 1.185 | 0.981-1.432 | 0.078 | 1.175 | 1.004-1.375 | 0.044 |
| Current vortex area (venous) | | 1.009 | 0.955-1.067 | 0.740 | 1.030 | 0.978-1.084 | 0.263 |
| Historical vortex area (venous) | | 0.998 | 0.958-1.040 | 0.932 | 1.028 | 0.984-1.075 | 0.218 |
| Age | Week 4 CRT | 1.019 | 0.956-1.085 | 0.569 | 1.031 | 0.962-1.104 | 0.394 |
| Current vortex area (venous) | | 1.030 | 0.995-1.066 | 0.098 | 1.011 | 0.981-1.041 | 0.488 |
| Historical vortex area (venous) | | 0.995 | 0.961-1.029 | 0.760 | 1.029 | 0.994-1.066 | 0.104 |
| Age | Week 5 CRT | 1.053 | 0.981-1.131 | 0.154 | 1.070 | 0.989-1.159 | 0.093 |
| Current vortex area (venous) | | 1.005 | 0.983-1.027 | 0.680 | 0.987 | 0.963-1.011 | 0.276 |
| Historical vortex area (venous) | | 1.036 | 0.988-1.088 | 0.146 | 1.062 | 1.001-1.127 | 0.047 |
| Age | Week 6 CRT | 1.031 | 0.957-1.110 | 0.425 | 1.049 | 0.969-1.137 | 0.238 |
| Current vortex area (venous) | | 1.006 | 0.977-1.036 | 0.689 | 0.995 | 0.967-1.024 | 0.755 |
| Historical vortex area (venous) | | 1.011 | 0.962-1.064 | 0.662 | 1.040 | 0.987-1.096 | 0.142 |
| Age | Pre-C1 | 1.047 | 0.969-1.133 | 0.246 | 1.107 | 0.991-1.238 | 0.073 |
| Current vortex area (venous) | | 1.001 | 0.978-1.024 | 0.935 | 0.976 | 0.950-1.002 | 0.071 |
| Historical vortex area (venous) | | 1.020 | 0.977-1.065 | 0.362 | 1.050 | 0.993-1.109 | 0.086 |
| Age | Pre-C2 | Inf | 0-Inf | N/A | 0.994 | 0.864-1.144 | 0.936 |
| Current vortex area (venous) | | Inf | 0-Inf | N/A | 1.049 | 0.986-1.116 | 0.134 |
| Historical vortex area (venous) | | Inf | 0-Inf | N/A | 0.959 | 0.905-1.016 | 0.157 |

* Refers to *normalized* tumor-to-reference tissue ratio. Reference tissue is defined as contralateral normal brain.

| | | | PFS | | | OS | |
|---|---|---|---|---|---|---|---|
| Co-variate | Timepoint | HR | 95% CI | P value | HR | 95% CI | P value |
| Age | Week 2 CRT | 1.174 | 1.004-1.372 | 0.044 | 1.029 | 0.934-1.132 | 0.564 |
| Current vortex area (arterial)* | | 1.009 | 0.984-1.034 | 0.490 | 1.006 | 0.983-1.031 | 0.601 |
| Historical vortex area (arterial)* | | 1.058 | 1.007-1.112 | 0.026 | 1.005 | 0.997-1.013 | 0.235 |
| Age | Week 3 CRT | 1.242 | 0.983-1.570 | 0.069 | 1.272 | 1.001-1.616 | 0.049 |
| Current vortex area (arterial) | | 0.993 | 0.983-1.003 | 0.167 | 0.994 | 0.983-1.004 | 0.241 |
| Historical vortex area (arterial) | | 1.011 | 0.998-1.025 | 0.088 | 1.012 | 1.000-1.023 | 0.048 |
| Age | Week 4 CRT | 1.008 | 0.923-1.101 | 0.861 | 1.004 | 0.912-1.105 | 0.935 |
| Current vortex area (arterial) | | 1.005 | 0.995-1.015 | 0.303 | 1.002 | 0.994-1.011 | 0.593 |
| Historical vortex area (arterial) | | 1.004 | 0.994-1.014 | 0.491 | 1.005 | 0.996-1.015 | 0.262 |
| Age | Week 5 CRT | 1.011 | 0.941-1.086 | 0.770 | 1.008 | 0.930-1.093 | 0.845 |
| Current vortex area (arterial) | | 1.000 | 0.987-1.012 | 0.979 | 0.996 | 0.987-1.006 | 0.434 |
| Historical vortex area (arterial) | | 1.005 | 0.995-1.016 | 0.310 | 1.008 | 0.999-1.017 | 0.075 |
| Age | Week 6 CRT | 1.020 | 0.943-1.104 | 0.620 | 1.024 | 0.944-1.110 | 0.573 |
| Current vortex area (arterial) | | 1.001 | 0.988-1.014 | 0.895 | 1.001 | 0.990-1.011 | 0.914 |
| Historical vortex area (arterial) | | 1.005 | 0.994-1.016 | 0.354 | 1.006 | 0.996-1.015 | 0.224 |
| Age | Pre-C1 | 1.020 | 0.941-1.105 | 0.631 | 1.049 | 0.943-1.166 | 0.383 |
| Current vortex area (arterial) | | 1.000 | 0.984-1.017 | 0.993 | 0.994 | 0.983-1.005 | 0.287 |
| Historical vortex area (arterial) | | 1.003 | 0.993-1.014 | 0.568 | 1.005 | 0.997-1.013 | 0.255 |
| Age | Pre-C2 | Inf | 0-Inf | N/A | 1.041 | 0.925-1.172 | 0.507 |
| Current vortex area (arterial) | | Inf | 0-Inf | N/A | 1.005 | 0.982-1.029 | 0.669 |
| Historical vortex area (arterial) | | Inf | 0-Inf | N/A | 1.000 | 0.990-1.011 | 0.979 |

* Refers to tumor-to-reference tissue ratio. Reference tissue is defined as contralateral normal brain.

| | | | PFS | | | OS | |
|---|---|---|---|---|---|---|---|
| Co-variate | Timepoint | HR | 95% CI | P value | HR | 95% CI | P value |
| Age | Week 2 CRT | 1.174 | 1.004-1.372 | 0.044 | 1.029 | 0.934-1.132 | 0.564 |
| Current vortex area (arterial)* | | 1.009 | 0.984-1.034 | 0.490 | 1.006 | 0.983-1.031 | 0.601 |
| Historical vortex area (arterial)* | | 1.058 | 1.007-1.112 | 0.026 | 1.005 | 0.997-1.013 | 0.235 |
| Age | Week 3 CRT | 1.242 | 0.983-1.570 | 0.069 | 1.272 | 1.001-1.616 | 0.049 |
| Current vortex area (arterial) | | 0.993 | 0.983-1.003 | 0.167 | 0.994 | 0.983-1.004 | 0.241 |
| Historical vortex area (arterial) | | 1.011 | 0.998-1.025 | 0.088 | 1.012 | 1.000-1.023 | 0.048 |
| Age | Week 4 CRT | 1.008 | 0.923-1.101 | 0.861 | 1.004 | 0.912-1.105 | 0.935 |
| Current vortex area (arterial) | | 1.005 | 0.995-1.015 | 0.303 | 1.002 | 0.994-1.011 | 0.593 |
| Historical vortex area (arterial) | | 1.004 | 0.994-1.014 | 0.491 | 1.005 | 0.996-1.015 | 0.262 |
| Age | Week 5 CRT | 1.011 | 0.941-1.086 | 0.770 | 1.008 | 0.930-1.093 | 0.845 |
| Current vortex area (arterial) | | 1.000 | 0.987-1.012 | 0.979 | 0.996 | 0.987-1.006 | 0.434 |
| Historical vortex area (arterial) | | 1.005 | 0.995-1.016 | 0.310 | 1.008 | 0.999-1.017 | 0.075 |
| Age | Week 6 CRT | 1.020 | 0.943-1.104 | 0.620 | 1.024 | 0.944-1.110 | 0.573 |
| Current vortex area (arterial) | | 1.001 | 0.988-1.014 | 0.895 | 1.001 | 0.990-1.011 | 0.914 |
| Historical vortex area (arterial) | | 1.005 | 0.994-1.016 | 0.354 | 1.006 | 0.996-1.015 | 0.224 |
| Age | Pre-C1 | 1.020 | 0.941-1.105 | 0.631 | 1.049 | 0.943-1.166 | 0.383 |
| Current vortex area (arterial) | | 1.000 | 0.984-1.017 | 0.993 | 0.994 | 0.983-1.005 | 0.287 |
| Historical vortex area (arterial) | | 1.003 | 0.993-1.014 | 0.568 | 1.005 | 0.997-1.013 | 0.255 |
| Age | Pre-C2 | Inf | 0-Inf | N/A | 1.041 | 0.925-1.172 | 0.507 |
| Current vortex area (arterial) | | Inf | 0-Inf | N/A | 1.005 | 0.982-1.029 | 0.669 |
| Historical vortex area (arterial) | | Inf | 0-Inf | N/A | 1.000 | 0.990-1.011 | 0.979 |

* Refers to *normalized* tumor-to-reference tissue ratio. Reference tissue is defined as contralateral normal brain.

| | | PFS | | | OS | | |
|---|---|---|---|---|---|---|---|
| Co-variate | Timepoint | HR | 95% CI | P value | HR | 95% CI | P value |
| Age | Week 2 CRT | 1.005 | 0.924-1.094 | 0.907 | 1.022 | 0.934-1.118 | 0.639 |
| Current vessel size* | | 0.982 | 0.940-1.027 | 0.433 | 1.015 | 0.975-1.057 | 0.459 |
| Historical vessel size* | | 0.989 | 0.934-1.048 | 0.715 | 0.985 | 0.928-1.047 | 0.631 |
| Age | Week 3 CRT | 1.233 | 1.016-1.495 | 0.034 | 1.214 | 1.014-1.454 | 0.035 |
| Current vessel size | | 0.979 | 0.933-1.027 | 0.375 | 0.972 | 0.926-1.022 | 0.269 |
| Historical vessel size | | 0.986 | 0.938-1.036 | 0.568 | 1.025 | 0.973-1.080 | 0.357 |
| Age | Week 4 CRT | 1.029 | 0.949-1.116 | 0.487 | 1.027 | 0.938-1.123 | 0.569 |
| Current vessel size | | 1.025 | 0.972-1.082 | 0.366 | 1.016 | 0.957-1.079 | 0.596 |
| Historical vessel size | | 0.970 | 0.900-1.045 | 0.422 | 0.992 | 0.909-1.083 | 0.859 |
| Age | Week 5 CRT | 1.023 | 0.950-1.101 | 0.549 | 1.017 | 0.947-1.092 | 0.648 |
| Current vessel size | | 1.003 | 0.960-1.048 | 0.894 | 0.981 | 0.937-1.027 | 0.405 |
| Historical vessel size | | 0.990 | 0.917-1.069 | 0.795 | 1.050 | 0.968-1.138 | 0.238 |
| Age | Week 6 CRT | 1.055 | 0.958-1.160 | 0.275 | 1.037 | 0.945-1.137 | 0.443 |
| Current vessel size | | 1.025 | 0.986-1.066 | 0.209 | 1.022 | 0.983-1.062 | 0.277 |
| Historical vessel size | | 0.985 | 0.936-1.038 | 0.578 | 0.992 | 0.942-1.044 | 0.746 |
| Age | Pre-C1 | 1.041 | 0.956-1.133 | 0.356 | 1.092 | 0.995-1.198 | 0.062 |
| Current vessel size | | 1.006 | 0.981-1.032 | 0.632 | 1.022 | 0.991-1.054 | 0.167 |
| Historical vessel size | | 0.986 | 0.929-1.048 | 0.658 | 1.026 | 0.955-1.102 | 0.489 |

* Refers to tumor-to-reference tissue ratio. Reference tissue is defined as contralateral normal brain.

| | | PFS | | | OS | | |
|---|---|---|---|---|---|---|---|
| Co-variate | Timepoint | HR | 95% CI | P value | HR | 95% CI | P value |
| Age | Week 2 CRT | 1.005 | 0.924-1.094 | 0.907 | 1.022 | 0.934-1.118 | 0.639 |
| Current vessel size* | | 0.982 | 0.940-1.027 | 0.433 | 1.015 | 0.975-1.057 | 0.459 |
| Historical vessel size* | | 0.989 | 0.934-1.048 | 0.715 | 0.985 | 0.928-1.047 | 0.631 |
| Age | Week 3 CRT | 1.233 | 1.016-1.495 | 0.034 | 1.214 | 1.014-1.454 | 0.035 |
| Current vessel size | | 0.979 | 0.933-1.027 | 0.375 | 0.972 | 0.926-1.022 | 0.269 |
| Historical vessel size | | 0.986 | 0.938-1.036 | 0.568 | 1.025 | 0.973-1.080 | 0.357 |
| Age | Week 4 CRT | 1.029 | 0.949-1.116 | 0.487 | 1.027 | 0.938-1.123 | 0.569 |
| Current vessel size | | 1.025 | 0.972-1.082 | 0.366 | 1.016 | 0.957-1.079 | 0.596 |
| Historical vessel size | | 0.97 | 0.900-1.045 | 0.422 | 0.992 | 0.909-1.083 | 0.859 |
| Age | Week 5 CRT | 1.023 | 0.950-1.101 | 0.549 | 1.017 | 0.947-1.092 | 0.648 |
| Current vessel size | | 1.003 | 0.960-1.048 | 0.894 | 0.981 | 0.937-1.027 | 0.405 |
| Historical vessel size | | 0.990 | 0.917-1.069 | 0.795 | 1.050 | 0.968-1.138 | 0.238 |
| Age | Week 6 CRT | 1.055 | 0.958-1.160 | 0.275 | 1.037 | 0.945-1.137 | 0.443 |
| Current vessel size | | 1.025 | 0.986-1.066 | 0.209 | 1.022 | 0.983-1.062 | 0.277 |
| Historical vessel size | | 0.985 | 0.936-1.038 | 0.578 | 0.992 | 0.942-1.044 | 0.746 |
| Age | Pre-C1 | 1.041 | 0.956-1.133 | 0.356 | 1.092 | 0.995-1.198 | 0.062 |
| Current vessel size | | 1.006 | 0.981-1.032 | 0.632 | 1.022 | 0.991-1.054 | 0.167 |
| Historical vessel size | | 0.986 | 0.929-1.048 | 0.658 | 1.026 | 0.955-1.102 | 0.489 |

* Refers to *normalized* tumor-to-reference tissue ratio. Reference tissue is defined as contralateral normal brain.

**Supplementary Table 5**. Cox regression analysis using age, current percent change in blood marker, and historical percent change in blood marker as co-variates and their correlation with progression-free (PFS) and overall survival (OS) at each time point during and after treatment. Lack of values and "Inf" (infinity) reflect a lack of convergence of the "coxph" function, which can occur when there are a small number of observations with similar values for a given co-variate. In these cases, p-values cannot be reported ("N/A").

| | | PFS | | | OS | | |
|---|---|---|---|---|---|---|---|
| Co-variate | Timepoint | HR | 95% CI | P value | HR | 95% CI | P value |
| Age | Week 2 CRT | 1.042 | 0.927-1.171 | 0.490 | 1.007 | 0.909-1.114 | 0.898 |
| Current bFGF | | 1.004 | 0.990-1.017 | 0.579 | 0.995 | 0.983-1.006 | 0.363 |
| Historical bFGF | | 0.991 | 0.958-1.025 | 0.600 | 1.001 | 0.970-1.032 | 0.970 |
| Age | Week 3 CRT | 1.035 | 0.960-1.116 | 0.370 | 1.045 | 0.968-1.129 | 0.259 |
| Current bFGF | | 1.002 | 0.998-1.007 | 0.325 | 1.002 | 0.998-1.007 | 0.378 |
| Historical bFGF | | 0.999 | 0.987-1.011 | 0.811 | 0.992 | 0.982-1.003 | 0.169 |
| Age | Week 4 CRT | 1.082 | 1.000-1.170 | 0.050 | 1.029 | 0.961-1.102 | 0.409 |
| Current bFGF | | 0.990 | 0.974-1.006 | 0.216 | 0.999 | 0.986-1.011 | 0.832 |
| Historical bFGF | | 0.978 | 0.956-1.001 | 0.063 | 0.985 | 0.966-1.004 | 0.121 |
| Age | Week 5 CRT | 1.038 | 0.962-1.120 | 0.340 | 1.050 | 0.966-1.142 | 0.254 |
| Current bFGF | | 0.997 | 0.986-1.008 | 0.590 | 1.001 | 0.989-1.013 | 0.890 |
| Historical bFGF | | 1.003 | 0.998-1.007 | 0.234 | 1.000 | 0.995-1.004 | 0.899 |
| Age | Week 6 CRT | 1.029 | 0.955-1.107 | 0.455 | 1.025 | 0.948-1.109 | 0.537 |
| Current bFGF | | 0.996 | 0.987-1.005 | 0.349 | 0.993 | 0.985-1.002 | 0.139 |
| Historical bFGF | | 1.003 | 0.998-1.008 | 0.185 | 1.002 | 0.998-1.007 | 0.323 |
| Age | Pre-C1 | 1.064 | 0.978-1.157 | 0.152 | 1.051 | 0.965-1.143 | 0.254 |
| Current bFGF | | 0.983 | 0.961-1.006 | 0.147 | 1.000 | 0.981-1.018 | 0.973 |
| Historical bFGF | | 1.008 | 0.999-1.017 | 0.078 | 1.000 | 0.993-1.008 | 0.933 |
| Age | Pre-C2 | 1.019 | 0.956-1.086 | 0.560 | 1.040 | 0.960-1.127 | 0.340 |
| Current bFGF | | 0.988 | 0.966-1.010 | 0.281 | 1.051 | 0.983-1.123 | 0.144 |
| Historical bFGF | | 1.006 | 0.994-1.018 | 0.316 | 0.947 | 0.883-1.016 | 0.131 |
| Age | Pre-C3 | 1.021 | 0.946-1.102 | 0.586 | 1.001 | 0.932-1.074 | 0.983 |
| Current bFGF | | 0.986 | 0.963-1.010 | 0.254 | 1.007 | 0.989-1.025 | 0.466 |
| Historical bFGF | | 1.006 | 0.994-1.019 | 0.295 | 0.989 | 0.974-1.004 | 0.165 |
| Age | Pre-C4 | 1.003 | 0.902-1.116 | 0.953 | 1.000 | 0.910-1.098 | 0.996 |
| Current bFGF | | 1.003 | 0.993-1.012 | 0.577 | 1.003 | 0.991-1.016 | 0.582 |
| Historical bFGF | | 1.003 | 0.990-1.015 | 0.683 | 0.988 | 0.969-1.007 | 0.225 |
| Age | Pre-C5 | Inf | 0-Inf | N/A | 1.009 | 0.910-1.118 | 0.870 |
| Current bFGF | | Inf | 0-Inf | N/A | 1.013 | 0.947-1.083 | 0.714 |
| Historical bFGF | | Inf | 0-Inf | N/A | 0.988 | 0.927-1.052 | 0.700 |
| Age | Pre-C6 | Inf | 0-Inf | N/A | Inf | 0-Inf | N/A |
| Current bFGF | | Inf | 0-Inf | N/A | Inf | 0-Inf | N/A |
| Historical bFGF | | Inf | 0-Inf | N/A | Inf | 0-Inf | N/A |
| Age | Post-C6 | Inf | 0-Inf | N/A | Inf | 0-Inf | N/A |
| Current bFGF | | Inf | 0-Inf | N/A | Inf | 0-Inf | N/A |
| Historical bFGF | | | | | | | |

| | | PFS | | | OS | | |
|---|---|---|---|---|---|---|---|
| Co-variate | Timepoint | HR | 95% CI | P value | HR | 95% CI | P value |
| Age | Week 2 CRT | 1.017 | 0.950-1.089 | 0.628 | 1.011 | 0.938-1.089 | 0.778 |
| Current PlGF | | 0.968 | 0.917-1.022 | 0.237 | 0.962 | 0.911-1.017 | 0.173 |
| Historical PlGF | | 1.020 | 0.971-1.072 | 0.432 | 0.980 | 0.937-1.024 | 0.362 |
| Age | Week 3 CRT | 1.042 | 0.968-1.121 | 0.275 | 1.051 | 0.974-1.133 | 0.201 |
| Current PlGF | | 0.997 | 0.961-1.036 | 0.892 | 0.983 | 0.947-1.019 | 0.348 |
| Historical PlGF | | 1.004 | 0.971-1.038 | 0.814 | 0.998 | 0.966-1.031 | 0.907 |
| Age | Week 4 CRT | 1.009 | 0.931-1.094 | 0.827 | 1.005 | 0.920-1.098 | 0.915 |
| Current PlGF | | 1.034 | 0.982-1.088 | 0.207 | 1.068 | 0.993-1.148 | 0.078 |
| Historical PlGF | | 0.970 | 0.919-1.088 | 0.269 | 0.957 | 0.908-1.010 | 0.108 |
| Age | Week 5 CRT | 1.048 | 0.968-1.134 | 0.245 | 1.048 | 0.969-1.134 | 0.240 |
| Current PlGF | | 1.009 | 0.980-1.038 | 0.548 | 1.003 | 0.975-1.032 | 0.850 |
| Historical PlGF | | 1.000 | 0.970-1.032 | 0.975 | 0.985 | 0.954-1.018 | 0.382 |
| Age | Week 6 CRT | 1.030 | 0.946-1.121 | 0.495 | 1.022 | 0.932-1.121 | 0.641 |
| Current PlGF | | 1.017 | 0.986-1.048 | 0.292 | 1.027 | 0.993-1.061 | 0.117 |
| Historical PlGF | | 1.003 | 0.976-1.031 | 0.817 | 0.975 | 0.949-1.003 | 0.077 |
| Age | Pre-C1 | 1.042 | 0.969-1.122 | 0.266 | 1.058 | 0.975-1.148 | 0.176 |
| Current PlGF | | 1.018 | 0.982-1.055 | 0.323 | 1.038 | 0.998-1.078 | 0.061 |
| Historical PlGF | | 1.005 | 0.976-1.036 | 0.731 | 0.985 | 0.961-1.010 | 0.244 |
| Age | Pre-C2 | 1.020 | 0.959-1.085 | 0.532 | 1.034 | 0.965-1.109 | 0.343 |
| Current PlGF | | 0.972 | 0.916-1.030 | 0.336 | 0.998 | 0.928-1.074 | 0.966 |
| Historical PlGF | | 1.008 | 0.974-1.043 | 0.658 | 0.959 | 0.913-1.006 | 0.088 |
| Age | Pre-C3 | 0.986 | 0.910-1.069 | 0.738 | 1.063 | 0.972-1.162 | 0.182 |
| Current PlGF | | 0.973 | 0.908-1.043 | 0.442 | 1.059 | 0.964-1.162 | 0.232 |
| Historical PlGF | | 1.004 | 0.961-1.049 | 0.861 | 0.898 | 0.801-1.007 | 0.065 |
| Age | Pre-C4 | 0.992 | 0.880-1.119 | 0.898 | 1.151 | 0.905-1.464 | 0.253 |
| Current PlGF | | 0.961 | 0.860-1.073 | 0.478 | 0.962 | 0.857-1.079 | 0.506 |
| Historical PlGF | | 1.100 | 0.937-1.290 | 0.244 | 0.717 | 0.443-1.161 | 0.176 |
| Age | Pre-C5 | 1.054 | 0.941-1.180 | 0.367 | Inf | 0-Inf | N/A |
| Current PlGF | | 0.976 | 0.918-1.038 | 0.441 | Inf | 0-Inf | N/A |
| Historical PlGF | | 0.929 | 0.774-1.114 | 0.427 | Inf | 0-Inf | N/A |
| Age | Pre-C6 | Inf | 0-Inf | N/A | Inf | 0-Inf | N/A |
| Current PlGF | | Inf | 0-Inf | N/A | Inf | 0-Inf | N/A |
| Historical PlGF | | Inf | 0-Inf | N/A | Inf | 0-Inf | N/A |
| Age | Post-C6 | Inf | 0-Inf | N/A | Inf | 0-Inf | N/A |
| Current PlGF | | Inf | 0-Inf | N/A | Inf | 0-Inf | N/A |
| Historical PlGF | | | | | | | |

| | | PFS | | | OS | | |
|---|---|---|---|---|---|---|---|
| Co-variate | Timepoint | HR | 95% CI | P value | HR | 95% CI | P value |
| Age | Week 2 CRT | 1.037 | 0.962-1.117 | 0.341 | 1.010 | 0.929-1.098 | 0.812 |
| Current sVEGFR1 | | 0.992 | 0.956-1.029 | 0.652 | 1.027 | 0.986-1.071 | 0.203 |
| Historical sVEGR1 | | 1.021 | 0.983-1.060 | 0.282 | 0.992 | 0.960-1.025 | 0.625 |
| Age | Week 3 CRT | 1.040 | 0.972-1.112 | 0.253 | 1.029 | 0.961-1.102 | 0.416 |
| Current sVEGFR1 | | 0.986 | 0.950-1.023 | 0.452 | 0.968 | 0.934-1.003 | 0.070 |
| Historical sVEGR1 | | 1.020 | 0.980-1.062 | 0.324 | 1.041 | 0.998-1.086 | 0.061 |
| Age | Week 4 CRT | 1.027 | 0.957-1.103 | 0.460 | 1.026 | 0.955-1.103 | 0.479 |
| Current sVEGFR1 | | 1.002 | 0.952-1.054 | 0.946 | 0.979 | 0.923-1.037 | 0.470 |
| Historical sVEGR1 | | 1.008 | 0.957-1.060 | 0.772 | 1.037 | 0.976-1.101 | 0.244 |
| Age | Week 5 CRT | 1.022 | 0.955-1.094 | 0.525 | 1.024 | 0.945-1.109 | 0.568 |
| Current sVEGFR1 | | 1.041 | 0.995-1.088 | 0.082 | 1.019 | 0.972-1.067 | 0.443 |
| Historical sVEGR1 | | 0.972 | 0.923-1.024 | 0.286 | 0.987 | 0.932-1.044 | 0.639 |
| Age | Week 6 CRT | 1.029 | 0.958-1.104 | 0.432 | 1.033 | 0.948-1.125 | 0.455 |
| Current sVEGFR1 | | 1.010 | 0.998-1.022 | 0.091 | 1.011 | 0.997-1.025 | 0.132 |
| Historical sVEGR1 | | 1.002 | 0.976-1.028 | 0.885 | 0.994 | 0.971-1.019 | 0.656 |
| Age | Pre-C1 | 1.04 | 0.967-1.119 | 0.293 | 1.043 | 0.958-1.135 | 0.332 |
| Current sVEGFR1 | | 1.005 | 0.997-1.012 | 0.229 | 1.004 | 0.997-1.012 | 0.274 |
| Historical sVEGR1 | | 1.005 | 0.982-1.029 | 0.669 | 0.999 | 0.978-1.020 | 0.920 |
| Age | Pre-C2 | 1.006 | 0.911-1.110 | 0.913 | 1.020 | 0.910-1.142 | 0.737 |
| Current sVEGFR1 | | 1.016 | 0.993-1.039 | 0.175 | 1.062 | 0.959-1.176 | 0.249 |
| Historical sVEGR1 | | 0.980 | 0.915-1.049 | 0.559 | 0.929 | 0.834-1.036 | 0.186 |
| Age | Pre-C3 | 0.989 | 0.888-1.101 | 0.843 | 0.966 | 0.874-1.067 | 0.494 |
| Current sVEGFR1 | | 1.009 | 0.966-1.053 | 0.700 | 1.025 | 0.983-1.069 | 0.248 |
| Historical sVEGR1 | | 0.977 | 0.885-1.080 | 0.653 | 0.950 | 0.881-1.024 | 0.182 |
| Age | Pre-C4 | 0.997 | 0.788-1.260 | 0.977 | 1.629 | 0.791-3.356 | 0.185 |
| Current sVEGFR1 | | 1.134 | 0.932-1.381 | 0.210 | 0.767 | 0.544-1.081 | 0.130 |
| Historical sVEGR1 | | 0.856 | 0.606-1.209 | 0.378 | 1.893 | 0.792-4.525 | 0.151 |
| Age | Pre-C5 | 1.224 | 0.907-1.651 | 0.186 | Inf | 0-Inf | N/A |
| Current sVEGFR1 | | 1.155 | 0.861-1.549 | 0.336 | Inf | 0-Inf | N/A |
| Historical sVEGR1 | | 0.924 | 0.711-1.200 | 0.552 | Inf | 0-Inf | N/A |
| Age | Pre-C6 | Inf | 0-Inf | N/A | Inf | 0-Inf | N/A |
| Current sVEGFR1 | | Inf | 0-Inf | N/A | Inf | 0-Inf | N/A |
| Historical sVEGR1 | | Inf | 0-Inf | N/A | Inf | 0-Inf | N/A |
| Age | Post-C6 | Inf | 0-Inf | N/A | Inf | 0-Inf | N/A |
| Current sVEGFR1 | | Inf | 0-Inf | N/A | Inf | 0-Inf | N/A |
| Historical sVEGR1 | | | | | | | |

| | | PFS | | | OS | | |
|---|---|---|---|---|---|---|---|
| Co-variate | Timepoint | HR | 95% CI | P value | HR | 95% CI | P value |
| Age | Week 2 CRT | 1.005 | 0.931-1.085 | 0.892 | 1.019 | 0.937-1.110 | 0.656 |
| Current VEGF | | 0.995 | 0.978-1.012 | 0.529 | 1.001 | 0.987-1.016 | 0.868 |
| Historical VEGF | | 1.020 | 0.986-1.056 | 0.254 | 0.994 | 0.970-1.017 | 0.593 |
| Age | Week 3 CRT | 1.052 | 0.980-1.129 | 0.164 | 1.030 | 0.961-1.103 | 0.401 |
| Current VEGF | | 0.989 | 0.976-1.002 | 0.104 | 0.986 | 0.972-1.000 | 0.043 |
| Historical VEGF | | 0.998 | 0.983-1.013 | 0.781 | 1.004 | 0.988-1.021 | 0.599 |
| Age | Week 4 CRT | 1.050 | 0.970-1.136 | 0.225 | 1.025 | 0.950-1.107 | 0.519 |
| Current VEGF | | 0.998 | 0.971-1.026 | 0.899 | 1.010 | 0.985-1.036 | 0.434 |
| Historical VEGF | | 0.986 | 0.971-1.002 | 0.093 | 0.987 | 0.970-1.004 | 0.143 |
| Age | Week 5 CRT | 1.046 | 0.971-1.127 | 0.233 | 1.038 | 0.962-1.120 | 0.336 |
| Current VEGF | | 1.005 | 0.994-1.017 | 0.369 | 1.009 | 0.995-1.022 | 0.216 |
| Historical VEGF | | 0.989 | 0.976-1.002 | 0.088 | 0.985 | 0.971-1.000 | 0.044 |
| Age | Week 6 CRT | 1.049 | 0.978-1.127 | 0.183 | 1.070 | 0.987-1.160 | 0.099 |
| Current VEGF | | 1.018 | 1.002-1.035 | 0.030 | 1.034 | 1.011-1.058 | 0.004 |
| Historical VEGF | | 0.978 | 0.958-0.998 | 0.030 | 0.961 | 0.935-0.989 | 0.006 |
| Age | Pre-C1 | 1.058 | 0.979-1.144 | 0.156 | 1.049 | 0.971-1.132 | 0.226 |
| Current VEGF | | 0.988 | 0.960-1.017 | 0.413 | 0.996 | 0.961-1.032 | 0.820 |
| Historical VEGF | | 1.000 | 0.988-1.011 | 0.965 | 0.997 | 0.980-1.014 | 0.724 |
| Age | Pre-C2 | 0.999 | 0.925-1.079 | 0.983 | 1.006 | 0.937-1.080 | 0.872 |
| Current VEGF | | 1.001 | 0.969-1.033 | 0.967 | 0.981 | 0.945-1.018 | 0.312 |
| Historical VEGF | | 1.005 | 0.991-1.019 | 0.519 | 1.006 | 0.991-1.021 | 0.443 |
| Age | Pre-C3 | 1.017 | 0.942-1.098 | 0.673 | 1.008 | 0.940-1.081 | 0.823 |
| Current VEGF | | 1.048 | 0.968-1.134 | 0.245 | 0.976 | 0.903-1.054 | 0.534 |
| Historical VEGF | | 0.971 | 0.927-1.016 | 0.202 | 1.001 | 0.961-1.043 | 0.954 |
| Age | Pre-C4 | Inf | 0-Inf | N/A | 1.004 | 0.912-1.106 | 0.928 |
| Current VEGF | | Inf | 0-Inf | N/A | 0.991 | 0.926-1.060 | 0.794 |
| Historical VEGF | | Inf | 0-Inf | N/A | 0.995 | 0.939-1.054 | 0.864 |
| Age | Pre-C5 | Inf | 0-Inf | N/A | Inf | 0-Inf | N/A |
| Current VEGF | | Inf | 0-Inf | N/A | Inf | 0-Inf | N/A |
| Historical VEGF | | Inf | 0-Inf | N/A | Inf | 0-Inf | N/A |
| Age | Pre-C6 | Inf | 0-Inf | N/A | Inf | 0-Inf | N/A |
| Current VEGF | | Inf | 0-Inf | N/A | Inf | 0-Inf | N/A |
| Historical VEGF | | Inf | 0-Inf | N/A | Inf | 0-Inf | N/A |
| Age | Post-C6 | Inf | 0-Inf | N/A | Inf | 0-Inf | N/A |
| Current VEGF | | Inf | 0-Inf | N/A | Inf | 0-Inf | N/A |
| Historical VEGF | | Inf | 0-Inf | N/A | Inf | 0-Inf | N/A |

| | | PFS | | | OS | | |
|---|---|---|---|---|---|---|---|
| Co-variate | Timepoint | HR | 95% CI | P value | HR | 95% CI | P value |
| Age | Week 2 CRT | 0.995 | 0.931-1.064 | 0.886 | 0.970 | 0.905-1.040 | 0.394 |
| Current SDF-1α | | 0.931 | 0.865-1.002 | 0.057 | 0.882 | 0.791-0.984 | 0.025 |
| Historical SDF-1α | | 1.079 | 0.986-1.181 | 0.098 | 1.034 | 0.952-1.122 | 0.427 |
| Age | Week 3 CRT | 1.030 | 0.957-1.109 | 0.432 | 1.015 | 0.941-1.095 | 0.697 |
| Current SDF-1α | | 1.000 | 0.967-1.035 | 0.983 | 0.988 | 0.957-1.020 | 0.456 |
| Historical SDF-1α | | 0.976 | 0.927-1.026 | 0.337 | 0.960 | 0.902-1.022 | 0.198 |
| Age | Week 4 CRT | 0.980 | 0.912-1.053 | 0.577 | 0.975 | 0.905-1.051 | 0.515 |
| Current SDF-1α | | 0.969 | 0.905-1.037 | 0.363 | 0.975 | 0.910-1.045 | 0.477 |
| Historical SDF-1α | | 0.943 | 0.863-1.031 | 0.198 | 0.949 | 0.868-1.037 | 0.244 |
| Age | Week 5 CRT | 1.022 | 0.954-1.094 | 0.536 | 1.007 | 0.939-1.079 | 0.849 |
| Current SDF-1α | | 1.017 | 0.991-1.044 | 0.195 | 1.050 | 1.005-1.098 | 0.030 |
| Historical SDF-1α | | 0.965 | 0.919-1.013 | 0.155 | 0.896 | 0.813-0.987 | 0.026 |
| Age | Week 6 CRT | 1.052 | 0.982-1.126 | 0.152 | 1.049 | 0.979-1.125 | 0.177 |
| Current SDF-1α | | 1.078 | 1.014-1.147 | 0.017 | 1.074 | 1.014-1.137 | 0.014 |
| Historical SDF-1α | | 0.908 | 0.838-0.983 | 0.017 | 0.890 | 0.818-0.968 | 0.006 |
| Age | Pre-C1 | 1.062 | 0.982-1.148 | 0.132 | 1.073 | 0.983-1.171 | 0.114 |
| Current SDF-1α | | 1.031 | 0.987-1.077 | 0.166 | 1.040 | 0.991-1.092 | 0.113 |
| Historical SDF-1α | | 0.981 | 0.951-1.012 | 0.232 | 0.965 | 0.932-0.999 | 0.046 |
| Age | Pre-C2 | 1.025 | 0.960-1.094 | 0.455 | 0.995 | 0.935-1.059 | 0.873 |
| Current SDF-1α | | 0.958 | 0.916-1.003 | 0.066 | 0.981 | 0.948-1.015 | 0.273 |
| Historical SDF-1α | | 1.030 | 1.003-1.058 | 0.029 | 0.993 | 0.970-1.016 | 0.550 |
| Age | Pre-C3 | 0.888 | 0.759-1.038 | 0.135 | 0.971 | 0.884-1.066 | 0.531 |
| Current SDF-1α | | 0.907 | 0.823-1.000 | 0.051 | 0.993 | 0.963-1.024 | 0.658 |
| Historical SDF-1α | | 1.039 | 1.001-1.079 | 0.044 | 0.978 | 0.936-1.021 | 0.315 |
| Age | Pre-C4 | 1.044 | 0.879-1.239 | 0.624 | 0.983 | 0.887-1.089 | 0.744 |
| Current SDF-1α | | 0.889 | 0.746-1.059 | 0.187 | 0.990 | 0.937-1.046 | 0.722 |
| Historical SDF-1α | | 1.122 | 0.963-1.308 | 0.14 | 0.984 | 0.922-1.049 | 0.619 |
| Age | Pre-C5 | Inf | 0-Inf | N/A | 1.041 | 0.929-1.167 | 0.487 |
| Current SDF-1α | | Inf | 0-Inf | N/A | 0.954 | 0.875-1.041 | 0.288 |
| Historical SDF-1α | | Inf | 0-Inf | N/A | 1.085 | 0.908-1.296 | 0.370 |
| Age | Pre-C6 | Inf | 0-Inf | N/A | Inf | 0-Inf | N/A |
| Current SDF-1α | | Inf | 0-Inf | N/A | Inf | 0-Inf | N/A |
| Historical SDF-1α | | Inf | 0-Inf | N/A | Inf | 0-Inf | N/A |
| Age | Post-C6 | Inf | 0-Inf | N/A | Inf | 0-Inf | N/A |
| Current SDF-1α | | Inf | 0-Inf | N/A | Inf | 0-Inf | N/A |
| Historical SDF-1α | | | | | | | |

| | | PFS | | | OS | | |
|---|---|---|---|---|---|---|---|
| Co-variate | Timepoint | HR | 95% CI | P value | HR | 95% CI | P value |
| Age | Week 2 CRT | 1.016 | 0.942-1.094 | 0.687 | 1.025 | 0.945-1.110 | 0.555 |
| Current Ang-2 | | 1.047 | 0.997-1.099 | 0.064 | 1.047 | 0.999-1.097 | 0.055 |
| Historical Ang-2 | | 0.930 | 0.853-1.013 | 0.096 | 0.907 | 0.830-0.990 | 0.029 |
| Age | Week 3 CRT | 1.053 | 0.967-1.148 | 0.232 | 1.075 | 0.973-1.186 | 0.154 |
| Current Ang-2 | | 1.006 | 0.979-1.034 | 0.659 | 1.018 | 0.988-1.050 | 0.244 |
| Historical Ang-2 | | 0.998 | 0.966-1.030 | 0.896 | 0.974 | 0.939-1.010 | 0.162 |
| Age | Week 4 CRT | 1.027 | 0.948-1.111 | 0.516 | 1.016 | 0.938-1.101 | 0.691 |
| Current Ang-2 | | 1.005 | 0.971-1.040 | 0.779 | 1.009 | 0.969-1.050 | 0.666 |
| Historical Ang-2 | | 0.979 | 0.920-1.041 | 0.495 | 0.968 | 0.898-1.042 | 0.387 |
| Age | Week 5 CRT | 1.037 | 0.960-1.120 | 0.360 | 1.045 | 0.959-1.140 | 0.314 |
| Current Ang-2 | | 1.025 | 0.989-1.062 | 0.176 | 1.066 | 1.016-1.117 | 0.009 |
| Historical Ang-2 | | 0.983 | 0.952-1.015 | 0.291 | 0.941 | 0.900-0.983 | 0.007 |
| Age | Week 6 CRT | 1.048 | 0.964-1.139 | 0.274 | 1.058 | 0.964-1.162 | 0.232 |
| Current Ang-2 | | 1.006 | 0.974-1.038 | 0.727 | 1.026 | 0.995-1.059 | 0.098 |
| Historical Ang-2 | | 1.001 | 0.973-1.029 | 0.953 | 0.977 | 0.952-1.003 | 0.085 |
| Age | Pre-C1 | 1.051 | 0.968-1.141 | 0.238 | 1.057 | 0.968-1.154 | 0.219 |
| Current Ang-2 | | 1.002 | 0.981-1.023 | 0.851 | 1.008 | 0.990-1.027 | 0.393 |
| Historical Ang-2 | | 1.005 | 0.995-1.016 | 0.332 | 0.996 | 0.987-1.005 | 0.415 |
| Age | Pre-C2 | 1.013 | 0.944-1.086 | 0.720 | 1.033 | 0.960-1.112 | 0.384 |
| Current Ang-2 | | 0.998 | 0.970-1.026 | 0.867 | 1.029 | 0.996-1.064 | 0.087 |
| Historical Ang-2 | | 1.007 | 0.997-1.017 | 0.184 | 0.987 | 0.971-1.004 | 0.123 |
| Age | Pre-C3 | 0.997 | 0.921-1.078 | 0.934 | 0.998 | 0.925-1.077 | 0.967 |
| Current Ang-2 | | 0.986 | 0.957-1.015 | 0.338 | 1.001 | 0.976-1.025 | 0.968 |
| Historical Ang-2 | | 1.014 | 0.998-1.031 | 0.091 | 0.992 | 0.976-1.009 | 0.358 |
| Age | Pre-C4 | 0.967 | 0.821-1.139 | 0.690 | 1.071 | 0.919-1.248 | 0.382 |
| Current Ang-2 | | 0.941 | 0.799-1.108 | 0.469 | 1.070 | 0.941-1.217 | 0.302 |
| Historical Ang-2 | | 1.092 | 0.901-1.322 | 0.369 | 0.920 | 0.797-1.062 | 0.256 |
| Age | Pre-C5 | Inf | 0-Inf | N/A | Inf | 0-Inf | N/A |
| Current Ang-2 | | Inf | 0-Inf | N/A | Inf | 0-Inf | N/A |
| Historical Ang-2 | | Inf | 0-Inf | N/A | Inf | 0-Inf | N/A |
| Age | Pre-C6 | Inf | 0-Inf | N/A | Inf | 0-Inf | N/A |
| Current Ang-2 | | | | | Inf | 0-Inf | N/A |
| Historical Ang-2 | | | | | Inf | 0-Inf | N/A |
| Age | Post-C6 | | | | Inf | 0-Inf | N/A |
| Current Ang-2 | | | | | | | |
| Historical Ang-2 | | | | | | | |

| | | PFS | | | OS | | |
|---|---|---|---|---|---|---|---|
| Co-variate | Timepoint | HR | 95% CI | P value | HR | 95% CI | P value |
| Age | Week 2 CRT | 1.007 | 0.923-1.098 | 0.872 | 0.992 | 0.907-1.084 | 0.855 |
| Current IL-1β | | 1.060 | 0.989-1.137 | 0.098 | 1.053 | 0.988-1.121 | 0.112 |
| Historical IL-1β | | 1.083 | 0.979-1.197 | 0.121 | 0.955 | 0.882-1.034 | 0.260 |
| Age | Week 3 CRT | 1.044 | 0.963-1.132 | 0.295 | 1.061 | 0.968-1.163 | 0.203 |
| Current IL-1β | | 1.015 | 0.987-1.043 | 0.297 | 1.022 | 0.993-1.052 | 0.143 |
| Historical IL-1β | | 0.993 | 0.945-1.044 | 0.797 | 0.984 | 0.938-1.033 | 0.525 |
| Age | Week 4 CRT | 1.014 | 0.942-1.091 | 0.714 | 1.000 | 0.926-1.080 | 0.996 |
| Current IL-1β | | 1.032 | 0.982-1.085 | 0.217 | 1.056 | 0.965-1.155 | 0.236 |
| Historical IL-1β | | 0.986 | 0.941-1.033 | 0.549 | 0.994 | 0.952-1.038 | 0.775 |
| Age | Week 5 CRT | 1.037 | 0.959-1.122 | 0.363 | 1.044 | 0.951-1.147 | 0.361 |
| Current IL-1β | | 1.007 | 0.972-1.044 | 0.693 | 1.013 | 0.974-1.053 | 0.528 |
| Historical IL-1β | | 1.003 | 0.963-1.046 | 0.876 | 1.001 | 0.958-1.046 | 0.956 |
| Age | Week 6 CRT | 1.041 | 0.960-1.128 | 0.332 | 1.047 | 0.955-1.148 | 0.325 |
| Current IL-1β | | 1.001 | 0.966-1.036 | 0.975 | 1.010 | 0.972-1.050 | 0.605 |
| Historical IL-1β | | 1.009 | 0.970-1.050 | 0.657 | 1.002 | 0.959-1.047 | 0.935 |
| Age | Pre-C1 | 1.037 | 0.958-1.122 | 0.370 | 1.063 | 0.959-1.177 | 0.245 |
| Current IL-1β | | 1.003 | 0.993-1.014 | 0.554 | 0.998 | 0.989-1.008 | 0.762 |
| Historical IL-1β | | 1.008 | 0.996-1.020 | 0.207 | 1.012 | 0.997-1.026 | 0.115 |
| Age | Pre-C2 | 1.010 | 0.934-1.093 | 0.796 | 1.037 | 0.939-1.145 | 0.474 |
| Current IL-1β | | 0.996 | 0.990-1.002 | 0.199 | 0.995 | 0.989-1.001 | 0.126 |
| Historical IL-1β | | 1.024 | 0.998-1.050 | 0.070 | 1.019 | 0.994-1.045 | 0.129 |
| Age | Pre-C3 | 0.988 | 0.917-1.064 | 0.746 | 0.982 | 0.897-1.075 | 0.695 |
| Current IL-1β | | 1.012 | 0.973-1.052 | 0.556 | 1.050 | 0.985-1.120 | 0.132 |
| Historical IL-1β | | 0.996 | 0.978-1.014 | 0.657 | 0.976 | 0.946-1.007 | 0.128 |
| Age | Pre-C4 | 0.982 | 0.870-1.110 | 0.776 | 0.620 | 0.221-1.738 | 0.363 |
| Current IL-1β | | 1.026 | 0.937-1.124 | 0.580 | 1.840 | 0.522-6.478 | 0.343 |
| Historical IL-1β | | 0.997 | 0.982-1.013 | 0.737 | 0.900 | 0.733-1.104 | 0.312 |
| Age | Pre-C5 | 1.009 | 0.867-1.173 | 0.909 | 1.014 | 0.861-1.194 | 0.869 |
| Current IL-1β | | 0.993 | 0.935-1.055 | 0.827 | 1.005 | 0.943-1.071 | 0.882 |
| Historical IL-1β | | 1.008 | 0.984-1.032 | 0.536 | 0.999 | 0.995-1.003 | 0.659 |
| Age | Pre-C6 | Inf | 0-Inf | N/A | Inf | 0-Inf | N/A |
| Current IL-1β | | Inf | 0-Inf | N/A | Inf | 0-Inf | N/A |
| Historical IL-1β | | Inf | 0-Inf | N/A | Inf | 0-Inf | N/A |
| Age | Post-C6 | Inf | 0-Inf | N/A | Inf | 0-Inf | N/A |
| Current IL-1β | | | | | | | |
| Historical IL-1β | | | | | | | |

| | | PFS | | | OS | | |
|---|---|---|---|---|---|---|---|
| Co-variate | Timepoint | HR | 95% CI | P value | HR | 95% CI | P value |
| Age | Week 2 CRT | 1.018 | 0.944-1.098 | 0.636 | 1.013 | 0.934-1.099 | 0.748 |
| Current IL-6 | | 0.999 | 0.987-1.011 | 0.862 | 1.003 | 0.993-1.013 | 0.571 |
| Historical IL-6 | | 1.004 | 0.997-1.011 | 0.236 | 0.998 | 0.992-1.003 | 0.441 |
| Age | Week 3 CRT | 1.038 | 0.957-1.126 | 0.364 | 1.041 | 0.957-1.133 | 0.349 |
| Current IL-6 | | 1.002 | 0.988-1.017 | 0.745 | 1.010 | 0.994-1.026 | 0.210 |
| Historical IL-6 | | 1.001 | 0.992-1.011 | 0.791 | 0.993 | 0.984-1.003 | 0.160 |
| Age | Week 4 CRT | 1.018 | 0.940-1.102 | 0.667 | 1.010 | 0.929-1.098 | 0.808 |
| Current IL-6 | | 1.004 | 0.998-1.011 | 0.212 | 1.010 | 0.999-1.021 | 0.083 |
| Historical IL-6 | | 0.998 | 0.985-1.012 | 0.829 | 0.996 | 0.983-1.010 | 0.60 |
| Age | Week 5 CRT | 1.034 | 0.956-1.118 | 0.404 | 1.030 | 0.948-1.121 | 0.484 |
| Current IL-6 | | 1.001 | 0.997-1.004 | 0.632 | 1.003 | 1.000-1.006 | 0.076 |
| Historical IL-6 | | 1.001 | 0.993-1.009 | 0.752 | 0.997 | 0.991-1.003 | 0.323 |
| Age | Week 6 CRT | 1.046 | 0.962-1.137 | 0.296 | 1.044 | 0.952-1.144 | 0.361 |
| Current IL-6 | | 0.997 | 0.990-1.003 | 0.343 | 1.004 | 0.998-1.010 | 0.168 |
| Historical IL-6 | | 1.004 | 0.998-1.010 | 0.162 | 0.999 | 0.994-1.003 | 0.507 |
| Age | Pre-C1 | 1.031 | 0.954-1.115 | 0.435 | 1.047 | 0.959-1.143 | 0.305 |
| Current IL-6 | | 0.995 | 0.981-1.010 | 0.536 | 0.998 | 0.985-1.012 | 0.790 |
| Historical IL-6 | | 1.002 | 0.999-1.004 | 0.140 | 1.001 | 0.999-1.004 | 0.290 |
| Age | Pre-C2 | 0.970 | 0.878-1.072 | 0.553 | 1.071 | 0.960-1.194 | 0.218 |
| Current IL-6 | | 0.990 | 0.966-1.014 | 0.402 | 1.021 | 0.995-1.049 | 0.119 |
| Historical IL-6 | | 1.005 | 1.000-1.010 | 0.075 | 0.999 | 0.996-1.003 | 0.711 |
| Age | Pre-C3 | 1.014 | 0.927-1.109 | 0.762 | 1.044 | 0.954-1.142 | 0.353 |
| Current IL-6 | | 1.007 | 0.9671.049 | 0.732 | 1.018 | 0.982-1.055 | 0.330 |
| Historical IL-6 | | 0.997 | 0.974-1.020 | 0.787 | 0.982 | 0.959-1.006 | 0.135 |
| Age | Pre-C4 | 1.047 | 0.903-1.214 | 0.542 | 0.990 | 0.870-1.127 | 0.881 |
| Current IL-6 | | 1.016 | 0.992-1.040 | 0.204 | 0.993 | 0.974-1.013 | 0.508 |
| Historical IL-6 | | 0.988 | 0.951-1.027 | 0.538 | 1.006 | 0.974-1.038 | 0.736 |
| Age | Pre-C5 | 1.133 | 0.821-1.563 | 0.449 | Inf | 0-Inf | N/A |
| Current IL-6 | | 1.055 | 0.964-1.154 | 0.246 | Inf | 0-Inf | N/A |
| Historical IL-6 | | 0.977 | 0.929-1.026 | 0.348 | Inf | 0-Inf | N/A |
| Age | Pre-C6 | Inf | 0-Inf | N/A | Inf | 0-Inf | N/A |
| Current IL-6 | | Inf | 0-Inf | N/A | Inf | 0-Inf | N/A |
| Historical IL-6 | | Inf | 0-Inf | N/A | Inf | 0-Inf | N/A |
| Age | Post-C6 | Inf | 0-Inf | N/A | Inf | 0-Inf | N/A |
| Current IL-6 | | | | | | | |
| Historical IL-6 | | | | | | | |

| | | PFS | | | OS | | |
|---|---|---|---|---|---|---|---|
| Co-variate | Timepoint | HR | 95% CI | P value | HR | 95% CI | P value |
| Age | Week 2 CRT | 1.034 | 0.957-1.117 | 0.401 | 1.029 | 0.951-1.113 | 0.479 |
| Current IL-8 | | 0.982 | 0.957-1.008 | 0.180 | 0.991 | 0.964-1.018 | 0.511 |
| Historical IL-8 | | 1.021 | 0.989-1.055 | 0.202 | 0.987 | 0.961-1.013 | 0.333 |
| Age | Week 3 CRT | 1.023 | 0.950-1.102 | 0.550 | 1.019 | 0.952-1.090 | 0.592 |
| Current IL-8 | | 0.984 | 0.962-1.007 | 0.168 | 0.965 | 0.935-0.995 | 0.025 |
| Historical IL-8 | | 1.012 | 0.993-1.032 | 0.221 | 1.019 | 0.998-1.039 | 0.073 |
| Age | Week 4 CRT | 1.064 | 0.974-1.163 | 0.170 | 1.030 | 0.949-1.117 | 0.482 |
| Current IL-8 | | 1.002 | 0.975-1.030 | 0.899 | 1.007 | 0.980-1.034 | 0.623 |
| Historical IL-8 | | 0.975 | 0.946-1.004 | 0.091 | 0.980 | 0.953-1.007 | 0.151 |
| Age | Week 5 CRT | 1.037 | 0.958-1.121 | 0.371 | 1.050 | 0.967-1.141 | 0.243 |
| Current IL-8 | | 0.999 | 0.989-1.010 | 0.883 | 1.003 | 0.993-1.012 | 0.581 |
| Historical IL-8 | | 1.003 | 0.986-1.021 | 0.702 | 0.996 | 0.979-1.012 | 0.602 |
| Age | Week 6 CRT | 1.028 | 0.947-1.116 | 0.511 | 1.048 | 0.962-1.142 | 0.284 |
| Current IL-8 | | 0.999 | 0.992-1.005 | 0.704 | 1.003 | 0.997-1.010 | 0.329 |
| Historical IL-8 | | 1.009 | 0.988-1.030 | 0.421 | 0.996 | 0.978-1.015 | 0.676 |
| Age | Pre-C1 | 1.082 | 0.978-1.197 | 0.125 | 1.064 | 0.960-1.179 | 0.238 |
| Current IL-8 | | 0.984 | 0.966-1.003 | 0.094 | 0.995 | 0.983-1.008 | 0.469 |
| Historical IL-8 | | 1.002 | 0.999-1.005 | 0.143 | 1.002 | 0.999-1.006 | 0.181 |
| Age | Pre-C2 | 1.024 | 0.852-1.231 | 0.801 | 0.881 | 0.740-1.050 | 0.158 |
| Current IL-8 | | 0.978 | 0.912-1.050 | 0.546 | 1.063 | 0.983-1.150 | 0.125 |
| Historical IL-8 | | 1.013 | 0.989-1.037 | 0.294 | 0.997 | 0.983-1.011 | 0.685 |
| Age | Pre-C3 | 0.987 | 0.874-1.115 | 0.835 | 1.049 | 0.934-1.178 | 0.423 |
| Current IL-8 | | 0.989 | 0.939-1.041 | 0.662 | 1.007 | 0.970-1.045 | 0.715 |
| Historical IL-8 | | 1.016 | 0.983-1.049 | 0.346 | 0.973 | 0.938-1.010 | 0.148 |
| Age | Pre-C4 | 1.399 | 0.740-2.648 | 0.302 | | | |
| Current IL-8 | | 0.925 | 0.803-1.067 | 0.285 | | | |
| Historical IL-8 | | 0.947 | 0.840-1.069 | 0.380 | | | |
| Age | Pre-C5 | Inf | 0-Inf | N/A | 0.907 | 0.512-1.606 | 0.738 |
| Current IL-8 | | Inf | 0-Inf | N/A | 1.045 | 0.938-1.165 | 0.423 |
| Historical IL-8 | | Inf | 0-Inf | N/A | 0.987 | 0.865-1.126 | 0.842 |
| Age | Pre-C6 | Inf | 0-Inf | N/A | Inf | 0-Inf | N/A |
| Current IL-8 | | Inf | 0-Inf | N/A | Inf | 0-Inf | N/A |
| Historical IL-8 | | Inf | 0-Inf | N/A | Inf | 0-Inf | N/A |
| Age | Post-C6 | Inf | 0-Inf | N/A | Inf | 0-Inf | N/A |
| Current IL-8 | | | | | | | |
| Historical IL-8 | | | | | | | |

| | | PFS | | | OS | | |
|---|---|---|---|---|---|---|---|
| Co-variate | Timepoint | HR | 95% CI | P value | HR | 95% CI | P value |
| Age | Week 2 CRT | 1.023 | 0.951-1.101 | 0.540 | 1.005 | 0.933-1.083 | 0.893 |
| Current TNF-α | | 0.987 | 0.948-1.027 | 0.524 | 0.994 | 0.957-1.032 | 0.748 |
| Historical TNF-α | | 1.010 | 0.992-1.028 | 0.297 | 0.993 | 0.978-1.009 | 0.374 |
| Age | Week 3 CRT | 1.046 | 0.963-1.135 | 0.286 | 1.046 | 0.962-1.137 | 0.289 |
| Current TNF-α | | 1.009 | 0.988-1.030 | 0.413 | 1.009 | 0.987-1.032 | 0.418 |
| Historical TNF-α | | 0.999 | 0.972-1.026 | 0.915 | 0.984 | 0.954-1.016 | 0.321 |
| Age | Week 4 CRT | 1.025 | 0.947-1.110 | 0.538 | 1.015 | 0.934-1.103 | 0.728 |
| Current TNF-α | | 1.007 | 0.999-1.017 | 0.102 | 1.013 | 1.000-1.027 | 0.056 |
| Historical TNF-α | | 0.982 | 0.945-1.022 | 0.377 | 0.987 | 0.953-1.022 | 0.470 |
| Age | Week 5 CRT | 1.036 | 0.958-1.120 | 0.378 | 1.044 | 0.955-1.140 | 0.345 |
| Current TNF-α | | 0.999 | 0.995-1.004 | 0.779 | 1.003 | 0.999-1.008 | 0.143 |
| Historical TNF-α | | 1.006 | 0.993-1.018 | 0.362 | 0.997 | 0.986-1.007 | 0.526 |
| Age | Week 6 CRT | 1.040 | 0.960-1.126 | 0.337 | 1.046 | 0.952-1.149 | 0.352 |
| Current TNF-α | | 0.998 | 0.987-1.009 | 0.676 | 1.005 | 0.995-1.016 | 0.330 |
| Historical TNF-α | | 1.002 | 0.998-1.007 | 0.333 | 1.000 | 0.995-1.005 | 0.951 |
| Age | Pre-C1 | 1.038 | 0.958-1.124 | 0.362 | 1.058 | 0.961-1.165 | 0.247 |
| Current TNF-α | | 1.003 | 0.984-1.023 | 0.733 | 1.009 | 0.991-1.028 | 0.328 |
| Historical TNF-α | | 1.001 | 0.999-1.004 | 0.283 | 1.002 | 0.999-1.004 | 0.187 |
| Age | Pre-C2 | 0.967 | 0.895-1.045 | 0.402 | 1.014 | 0.929-1.106 | 0.761 |
| Current TNF-α | | 0.946 | 0.900-0.994 | 0.029 | 1.010 | 0.988-1.032 | 0.391 |
| Historical TNF-α | | 1.014 | 1.003-1.026 | 0.016 | 1.001 | 0.996-1.006 | 0.610 |
| Age | Pre-C3 | 0.996 | 0.927-1.069 | 0.903 | 1.006 | 0.939-1.078 | 0.861 |
| Current TNF-α | | 0.985 | 0.952-1.018 | 0.368 | 0.981 | 0.951-1.013 | 0.239 |
| Historical TNF-α | | 1.000 | 0.987-1.012 | 0.945 | 0.990 | 0.976-1.004 | 0.174 |
| Age | Pre-C4 | 1.001 | 0.850-1.179 | 0.990 | 0.959 | 0.825-1.115 | 0.585 |
| Current TNF-α | | 1.013 | 0.890-1.152 | 0.850 | 1.120 | 0.934-1.342 | 0.221 |
| Historical TNF-α | | 1.002 | 0.986-1.019 | 0.820 | 0.985 | 0.963-1.007 | 0.185 |
| Age | Pre-C5 | Inf | 0-Inf | N/A | 0.934 | 0.817-1.067 | 0.316 |
| Current TNF-α | | Inf | 0-Inf | N/A | 0.922 | 0.771-1.102 | 0.373 |
| Historical TNF-α | | Inf | 0-Inf | N/A | 1.029 | 0.975-1.086 | 0.298 |
| Age | Pre-C6 | Inf | 0-Inf | N/A | Inf | 0-Inf | N/A |
| Current TNF-α | | Inf | 0-Inf | N/A | Inf | 0-Inf | N/A |
| Historical TNF-α | | Inf | 0-Inf | N/A | Inf | 0-Inf | N/A |
| Age | Post-C6 | Inf | 0-Inf | N/A | Inf | 0-Inf | N/A |
| Current TNF-α | | | | | | | |
| Historical TNF-α | | | | | | | |

| | | PFS | | | OS | | |
|---|---|---|---|---|---|---|---|
| Co-variate | Timepoint | HR | 95% CI | P value | HR | 95% CI | P value |
| Age | Week 2 CRT | 1.019 | 0.954-1.090 | 0.572 | 1.011 | 0.940-1.089 | 0.762 |
| Current CAIX | | 0.986 | 0.967-1.006 | 0.169 | 0.988 | 0.970-1.007 | 0.225 |
| Historical CAIX | | 1.021 | 0.988-1.055 | 0.223 | 0.990 | 0.964-1.017 | 0.463 |
| Age | Week 3 CRT | 1.038 | 0.973-1.107 | 0.262 | 1.047 | 0.979-1.120 | 0.182 |
| Current CAIX | | 0.997 | 0.979-1.015 | 0.714 | 0.983 | 0.961-1.005 | 0.135 |
| Historical CAIX | | 0.991 | 0.962-1.021 | 0.566 | 0.999 | 0.971-1.027 | 0.931 |
| Age | Week 4 CRT | 1.019 | 0.949-1.095 | 0.598 | 0.996 | 0.925-1.072 | 0.914 |
| Current CAIX | | 1.007 | 0.984-1.031 | 0.545 | 1.021 | 0.994-1.049 | 0.131 |
| Historical CAIX | | 0.983 | 0.958-1.009 | 0.190 | 0.969 | 0.941-0.997 | 0.033 |
| Age | Week 5 CRT | 1.032 | 0.969-1.099 | 0.331 | 1.040 | 0.971-1.113 | 0.263 |
| Current CAIX | | 0.973 | 0.953-0.994 | 0.012 | 0.995 | 0.982-1.008 | 0.456 |
| Historical CAIX | | 1.015 | 0.999-1.030 | 0.060 | 0.994 | 0.980-1.007 | 0.346 |
| Age | Week 6 CRT | 1.036 | 0.969-1.109 | 0.300 | 1.042 | 0.971-1.117 | 0.254 |
| Current CAIX | | 0.996 | 0.982-1.011 | 0.597 | 0.993 | 0.977-1.008 | 0.357 |
| Historical CAIX | | 0.996 | 0.983-1.010 | 0.593 | 0.992 | 0.979-1.005 | 0.224 |
| Age | Pre-C1 | 1.035 | 0.966-1.108 | 0.330 | 1.041 | 0.967-1.121 | 0.284 |
| Current CAIX | | 1.001 | 0.989-1.014 | 0.813 | 1.007 | 0.995-1.019 | 0.275 |
| Historical CAIX | | 0.993 | 0.982-1.004 | 0.198 | 0.983 | 0.969-0.997 | 0.016 |
| Age | Pre-C2 | 1.007 | 0.930-1.090 | 0.863 | 1.018 | 0.928-1.117 | 0.706 |
| Current CAIX | | 1.009 | 0.984-1.034 | 0.478 | 1.021 | 1.000-1.043 | 0.050 |
| Historical CAIX | | 1.011 | 0.989-1.033 | 0.340 | 0.984 | 0.963-1.005 | 0.142 |
| Age | Pre-C3 | 0.998 | 0.924-1.078 | 0.962 | 0.985 | 0.891-1.090 | 0.771 |
| Current CAIX | | 1.000 | 0.980-1.020 | 0.979 | 0.984 | 0.956-1.012 | 0.252 |
| Historical CAIX | | 1.010 | 0.987-1.034 | 0.403 | 0.982 | 0.950-1.014 | 0.263 |
| Age | Pre-C4 | 0.938 | 0.782-1.126 | 0.493 | Inf | 0-Inf | N/A |
| Current CAIX | | 0.942 | 0.830-1.068 | 0.350 | Inf | 0-Inf | N/A |
| Historical CAIX | | 1.039 | 0.981-1.102 | 0.191 | Inf | 0-Inf | N/A |
| Age | Pre-C5 | Inf | 0-Inf | N/A | Inf | 0-Inf | N/A |
| Current CAIX | | Inf | 0-Inf | N/A | Inf | 0-Inf | N/A |
| Historical CAIX | | Inf | 0-Inf | N/A | Inf | 0-Inf | N/A |
| Age | Pre-C6 | Inf | 0-Inf | N/A | Inf | 0-Inf | N/A |
| Current CAIX | | Inf | 0-Inf | N/A | Inf | 0-Inf | N/A |
| Historical CAIX | | Inf | 0-Inf | N/A | Inf | 0-Inf | N/A |
| Age | Post-C6 | Inf | 0-Inf | N/A | Inf | 0-Inf | N/A |
| Current CAIX | | | | | | | |
| Historical CAIX | | | | | | | |

| | | PFS | | | OS | | |
|---|---|---|---|---|---|---|---|
| Co-variate | Timepoint | HR | 95% CI | P value | HR | 95% CI | P value |
| Age | Week 2 CRT | 1.077 | 0.986-1.176 | 0.100 | 1.014 | 0.937-1.096 | 0.732 |
| Current sTie-2 | | 1.020 | 0.954-1.089 | 0.566 | 0.987 | 0.933-1.044 | 0.647 |
| Historical sTie-2 | | 1.126 | 0.991-1.279 | 0.068 | 1.019 | 0.936-1.110 | 0.659 |
| Age | Week 3 CRT | 1.059 | 0.972-1.154 | 0.190 | 1.054 | 0.952-1.166 | 0.312 |
| Current sTie-2 | | 0.973 | 0.916-1.034 | 0.375 | 1.008 | 0.951-1.068 | 0.798 |
| Historical sTie-2 | | 1.069 | 0.997-1.147 | 0.061 | 0.989 | 0.940-1.041 | 0.680 |
| Age | Week 4 CRT | 1.033 | 0.964-1.107 | 0.355 | 1.008 | 0.935-1.086 | 0.833 |
| Current sTie-2 | | 0.944 | 0.885-1.007 | 0.080 | 0.975 | 0.927-1.025 | 0.321 |
| Historical sTie-2 | | 1.075 | 0.975-1.185 | 0.146 | 1.033 | 0.948-1.126 | 0.455 |
| Age | Week 5 CRT | 1.029 | 0.957-1.106 | 0.439 | 1.030 | 0.950-1.117 | 0.476 |
| Current sTie-2 | | 1.034 | 0.977-1.093 | 0.248 | 1.068 | 1.000-1.140 | 0.049 |
| Historical sTie-2 | | 0.945 | 0.855-1.044 | 0.263 | 0.897 | 0.803-1.002 | 0.054 |
| Age | Week 6 CRT | 1.042 | 0.963-1.127 | 0.303 | 1.051 | 0.964-1.147 | 0.261 |
| Current sTie-2 | | 1.007 | 0.965-1.051 | 0.752 | 1.031 | 0.985-1.079 | 0.188 |
| Historical sTie-2 | | 0.990 | 0.922-1.062 | 0.769 | 0.956 | 0.891-1.026 | 0.211 |
| Age | Pre-C1 | 1.043 | 0.961-1.132 | 0.314 | 1.065 | 0.960-1.182 | 0.234 |
| Current sTie-2 | | 1.007 | 0.974-1.041 | 0.683 | 1.018 | 0.981-1.057 | 0.334 |
| Historical sTie-2 | | 0.996 | 0.953-1.041 | 0.850 | 0.998 | 0.953-1.046 | 0.943 |
| Age | Pre-C2 | 1.012 | 0.943-1.087 | 0.735 | 1.011 | 0.924-1.107 | 0.807 |
| Current sTie-2 | | 1.003 | 0.961-1.047 | 0.892 | 1.027 | 0.976-1.080 | 0.305 |
| Historical sTie-2 | | 0.999 | 0.937-1.065 | 0.978 | 0.957 | 0.880-1.040 | 0.299 |
| Age | Pre-C3 | 0.989 | 0.918-1.065 | 0.772 | 0.989 | 0.904-1.084 | 0.820 |
| Current sTie-2 | | 0.984 | 0.934-1.036 | 0.538 | 1.021 | 0.965-1.081 | 0.467 |
| Historical sTie-2 | | 0.984 | 0.918-1.055 | 0.653 | 0.940 | 0.856-1.033 | 0.201 |
| Age | Pre-C4 | 1.019 | 0.934-1.113 | 0.668 | 0.979 | 0.874-1.097 | 0.719 |
| Current sTie-2 | | 1.000 | 0.960-1.042 | 0.991 | 1.010 | 0.945-1.080 | 0.762 |
| Historical sTie-2 | | 0.964 | 0.870-1.069 | 0.489 | 0.938 | 0.807-1.091 | 0.405 |
| Age | Pre-C5 | 1.079 | 0.933-1.247 | 0.305 | 1.014 | 0.906-1.135 | 0.807 |
| Current sTie-2 | | 1.013 | 0.940-1.090 | 0.741 | 1.027 | 0.949-1.111 | 0.510 |
| Historical sTie-2 | | 0.898 | 0.749-1.077 | 0.245 | 1.025 | 0.930-1.130 | 0.616 |
| Age | Pre-C6 | Inf | 0-Inf | N/A | Inf | 0-Inf | N/A |
| Current sTie-2 | | Inf | 0-Inf | N/A | Inf | 0-Inf | N/A |
| Historical sTie-2 | | Inf | 0-Inf | N/A | Inf | 0-Inf | N/A |
| Age | Post-C6 | Inf | 0-Inf | N/A | Inf | 0-Inf | N/A |
| Current sTie-2 | | | | | | | |
| Historical sTie-2 | | | | | | | |

**Supplementary Table 6**. Spearman's correlation between imaging and blood markers. Imaging markers that were found to have a significant association with clinical outcome at any given time point were tested for their correlation with blood markers at the same time point (and vice versa for blood markers).

Spearman's rho correlation coefficient (ρ) between baseline microvascular rCBV within FLAIR ROI and

- Ang2: ρ = 0.3406593, p = 0.2334283
- IL-1beta: ρ = -0.01792957, p = 0.9514901
- IL-6: ρ = 0.4702033, p = 0.08975866
- IL-8: ρ = 0.01980199, p = 0.9464304
- TNF_alpha: ρ = 0.5076923, p = 0.06669017
- hCAIX: ρ = -0.3784381, p = 0.1821242
- hTie-2: ρ = 0.1296703, p = 0.6595064
- bFGF: ρ = 0.3186813, p = 0.2665382
- PlGF: ρ = -0.2263736, p = 0.4356143
- sFLT1: ρ = -0.173626, p = 0.5525664
- VEGF: ρ = 0.3098901, p = 0.2805603
- SDF_1a: ρ = 0.5032967, p = 0.06937541

Spearman's rho correlation coefficient (ρ) between baseline microvascular rCBF within FLAIR ROI and

- Ang2: ρ = 0.1692308, p = 0.5629029
- IL-1beta: ρ = -0.05891145, p = 0.8414435
- IL-6: ρ = 0.3289215, p = 0.2508524
- IL-8: ρ = 0.0726073, p = 0.8051658
- TNF_alpha: ρ = 0.578022, p = 0.03337018
- hCAIX: ρ = -0.2354237, p = 0.4178068
- hTie-2: ρ = 0.1648352, p = 0.5733234
- bFGF: ρ = 0.3054945, p = 0.2877382
- PlGF: ρ = -0.4021978, p = 0.1552627
- sFLT1: ρ = -0.367033, p = 0.1973395
- VEGF: ρ = 0.2087912, p = 0.4730802
- SDF_1a: ρ = 0.2791209, p = 0.3331323

Spearman's rho correlation coefficient (ρ) between baseline panvascular rCBV within FLAIR ROI and

- Ang2: ρ = 0.243956, p = 0.3997646
- IL-1beta: ρ = 0.07940239, p = 0.7872968
- IL-6: ρ = 0.5629194, p = 0.0360956
- IL-8: ρ = 0.2860288, p = 0.3215103
- TNF_alpha: ρ = 0.4549451, p = 0.1043803
- hCAIX: ρ = -0.2530255, p = 0.3827738
- hTie-2: ρ = 0.178022, p = 0.5423156
- bFGF: ρ = 0.2043956, p = 0.4826912

- PlGF: ρ = -0.1252747, p = 0.6706044
- sFLT1: ρ = -0.07252747, p = 0.8083139
- VEGF: ρ = 0.2527473, p = 0.3824644
- SDF_1a: ρ = 0.3714286, p = 0.1917066

Spearman's rho correlation coefficient (ρ) between baseline panvascular rCBF within FLAIR ROI and

- Ang2: ρ = 0.3098901, p = 0.2805603
- IL-1beta: ρ = 0.1152615, p = 0.694783
- IL-6: ρ = 0.5585043, p = 0.03790612
- IL-8: ρ = 0.01760177, p = 0.9523761
- TNF_alpha: ρ = 0.5032967, p = 0.06937541
- hCAIX: ρ = -0.407041, p = 0.1486116
- hTie-2: ρ = 0.07692308, p = 0.7965664
- bFGF: ρ = 0.3142857, p = 0.2734936
- PlGF: ρ = -0.2659341, p = 0.3573121
- sFLT1: ρ = -0.1692308, p = 0.5629029
- VEGF: ρ = 0.2395604, p = 0.4085722
- SDF_1a: ρ = 0.3934066, p = 0.1651481

Spearman's rho correlation coefficient (ρ) between week 5 $K^{trans}$ within tumor (T1 post-contrast) ROI and

- Ang2: ρ = -0.04945055, p = 0.877523
- IL-1beta: ρ = 0.4599331, p = 0.1137951
- IL-6: ρ = 0.1098901, p = 0.7232481
- IL-8: ρ = 0.01098901, p = 0.9783115
- TNF_alpha: ρ = -0.03296703, p = 0.9205854
- hCAIX: ρ = -0.1483516, p = 0.6297581
- hTie-2: 5 ρ = -0.5054945, p = 0.08121512
- bFGF: ρ = 0.3406593, p = 0.2549977
- PlGF: ρ = 0.1373626, p = 0.6560004
- sFLT1: ρ = -0.03846154, p = 0.9062021
- VEGF: ρ = 0.08791209, p = 0.7785013
- SDF_1a: ρ = -0.08791209, p = 0.7785013

Spearman's rho correlation coefficient (ρ) between week 6 VEGF level and imaging markers in tumor (T1 post-contrast) ROI:

- ADC: ρ = 0.5363636, p = 0.0936425
- $K^{trans}$: ρ = -0.01818182, p = 0.9675762
- Microvascular rCBV: ρ = -0.2454545, p = 0.4681835
- Microvascular rCBF: ρ = -0.6818182, p = 0.02547558
- Panvascular rCBV: ρ = -0.4363636, p = 0.1825319
- Panvascular rCBF: ρ = -0.5363636, p = 0.0936425

Spearman's rho correlation coefficient (ρ) between week 6 VEGF level and imaging markers in edema (FLAIR) ROI:

- ADC: $\rho = 0.05454545$, $p = 0.8814724$
- Microvascular rCBV: $\rho = 0.4818182$, $p = 0.1372192$
- Microvascular rCBF: $\rho = 0.4454545$, $p = 0.1727865$
- Panvascular rCBV: $\rho = 0.4545455$, $p = 0.163386$
- Panvascular rCBF: $\rho = 0.4$, $p = 0.2250141$
